# Supplementary material for: Locally anchoring enzymes to tissues via extracellular glycan recognition
Source: Nat Commun. 2018 Nov 22;9:4943. doi: 10.1038/s41467-018-07129-6 (PMC6250738; doi:10.1038/s41467-018-07129-6)
Supplement: Supplementary file 1 — Supplementary Information [file 41467_2018_7129_MOESM1_ESM.pdf]

Supplementary information

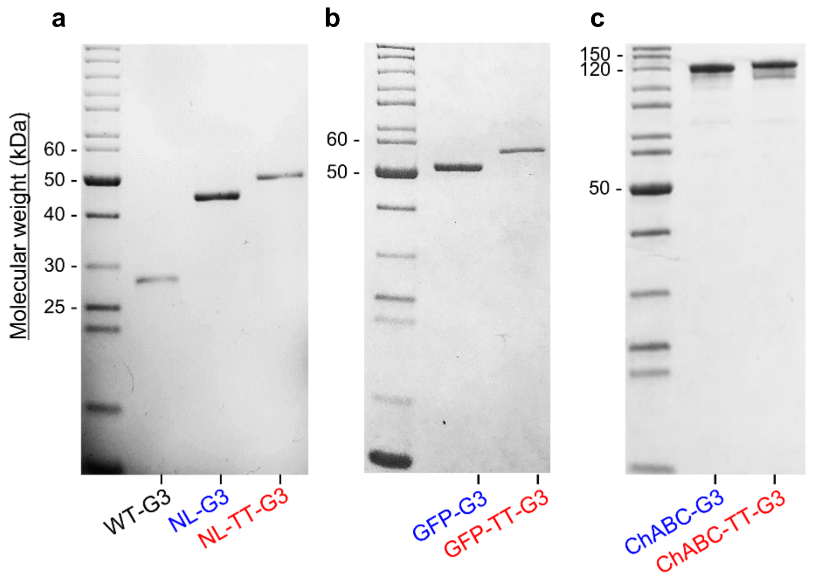

Supplementary Figure 1. SDS-PAGE gels of monomeric G3 fusion proteins and trimeric nanoassemblies compared to wild-type galectin-3 (WT-G3). **a** WT-G3, NL-G3, and NL-TT-G3; **b** GFP-G3 and GFP-TT-G3; **c** ChABC-G3 and ChABC-TT-G3.

Supplementary Table 1. Theoretical molecular weights of G3 fusions

G3 fusion proteins	Theoretical denatured molecular weight (kDa)	Theoretical native molecular weight (kDa)
NL-G3	47.4	47.4
GFP-G3	54.8	54.8
ChABC-G3	140.8	140.8
NL-TT-G3	52.7	158.1
GFP-TT-G3	60.3	180.9
ChABC-TT-G3	146.2	438.6

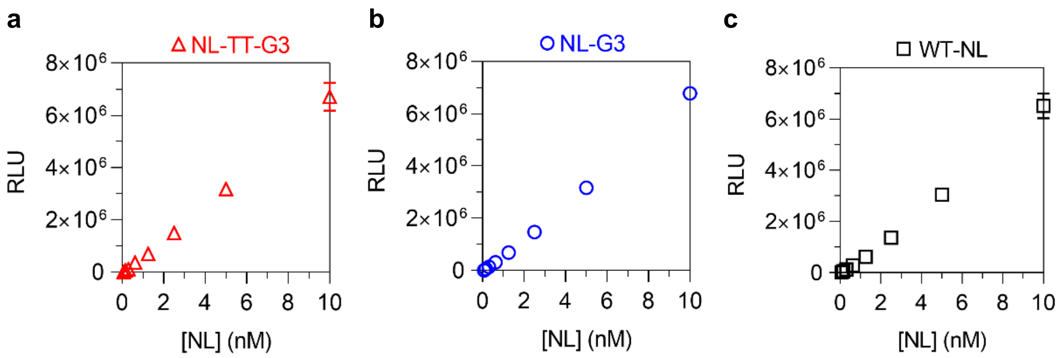

Supplementary Figure 2. Luciferase activity of monomeric G3 fusion proteins and trimeric nanoassemblies compared to wild-type NanoLuc™ (WT-NL). **a** NL-TT-G3 (red triangles), **b** NL-G3 (blue circles), and **c** WT-NL (black squares). N = 3, mean ± s.d.

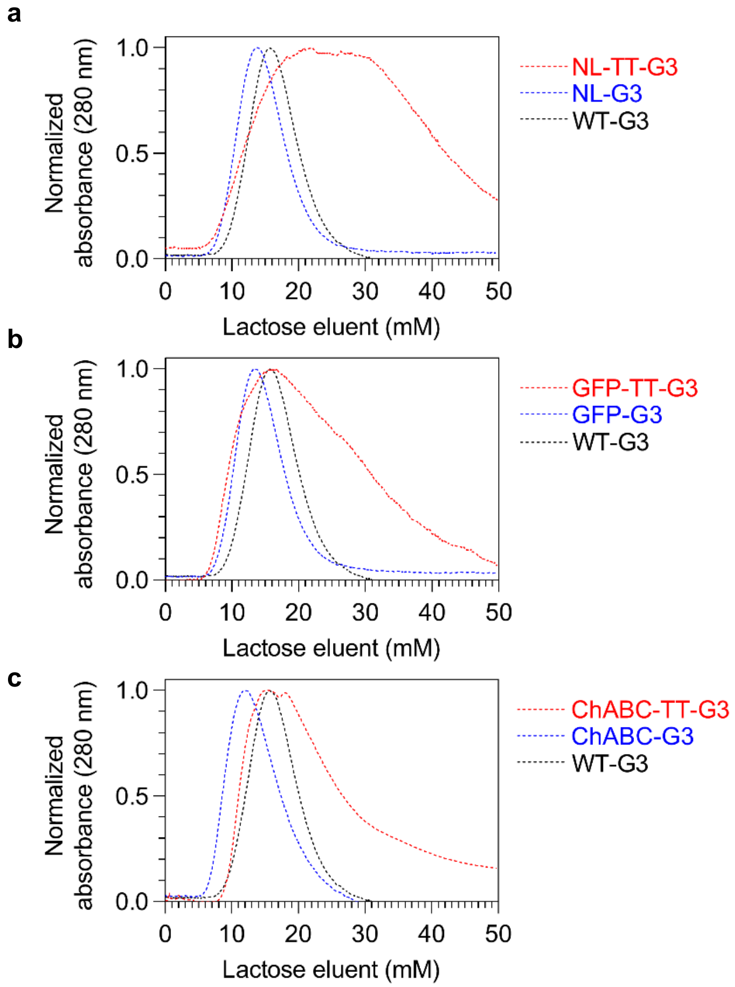

Supplementary Figure 3. Lactose affinity chromatography of monomeric G3 fusion proteins and trimeric nanoassemblies compared to wild-type galectin-3 (WT-G3). **a** NL-TT-G3, NL-G3, and WT-G3; **b** GFP-TT-G3, GFP-G3, and WT-G3; **c** ChABC-TT-G3, ChABC-G3, and WT-G3. Trimeric nanoassemblies are presented as red dashed lines, monomeric G3 fusion proteins are presented as blue dashed lines, and WT-G3 is presented as black dashed lines.

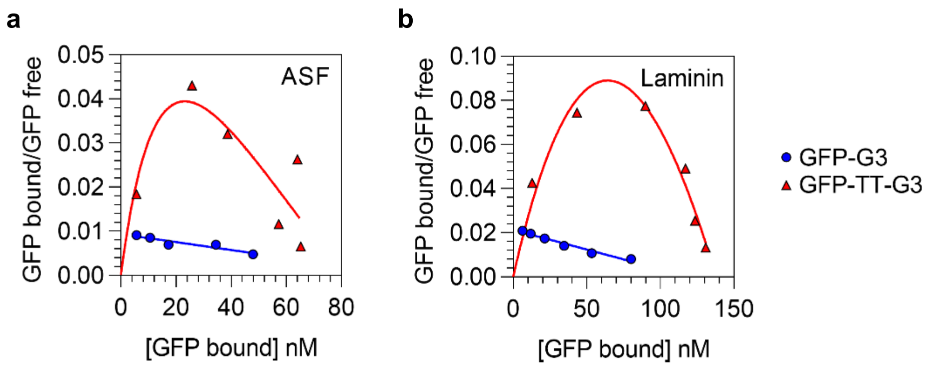

Supplementary Figure 4. Scatchard analysis of saturating binding data from Figure 3e for monomeric G3 fusion proteins and trimeric nanoassemblies binding to adsorbed glycoprotein. **a** GFP-G3 and GFP-TT-G3 binding to asialofetuin (ASF). **b** GFP-G3 and GFP-TT-G3 binding to laminin. GFP-G3 is presented as blue circles and GFP-TT-G3 is presented as red triangles.

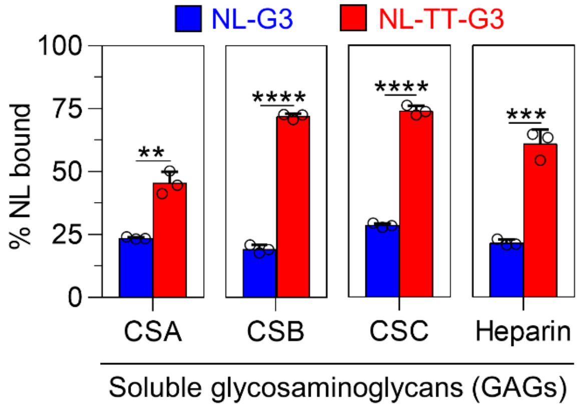

Supplementary Figure 5. GAG binding competition assay. NL-G3 or NL-TT-G3 were mixed with chondroitin sulfate-A (CSA), chondroitin sulfate-B (CSB), chondroitin sulfate-C (CSC), or heparin and then added to laminin-coated plates. % NL bound represents [NL] bound to laminin versus GAG. N = 3, mean ± s.d., **p < 0.01, ***p < 0.001, ****p < 0.0001, Student's t-test between fusions for each GAG group. Data points at or above baseline signal are shown as open circles. NL-G3 is presented as blue bars and NL-TT-G3 is presented as red bars.

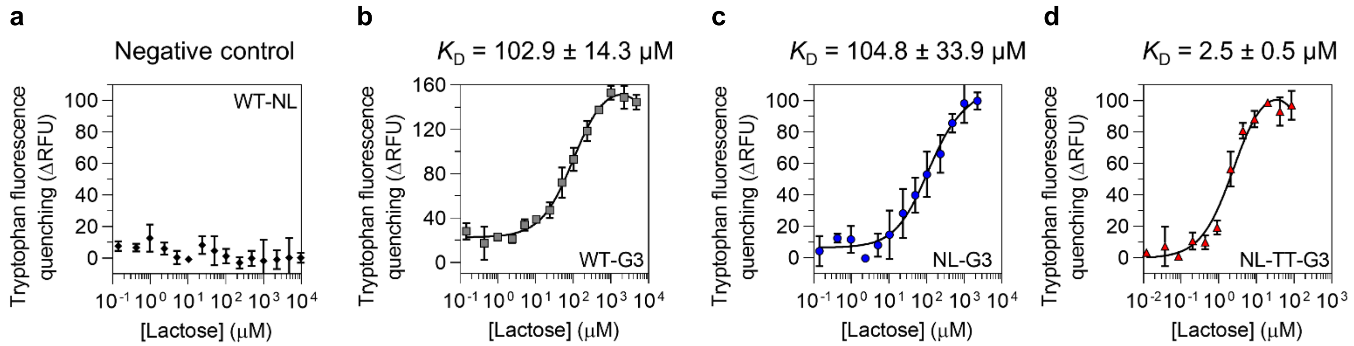

Supplementary Figure 6. Tryptophan fluorescence quenching of monomeric G3 fusion proteins and trimeric nanoassemblies due to binding to soluble lactose. **a** Wild-type NanoLuc™ (WT-NL, black diamonds, negative control), **b** Wild-type galectin-3 (WT-G3, gray squares), **c** NL-G3 (blue circles), and **d** NL-TT-G3 (red triangles). N = 3, mean ± s.d.

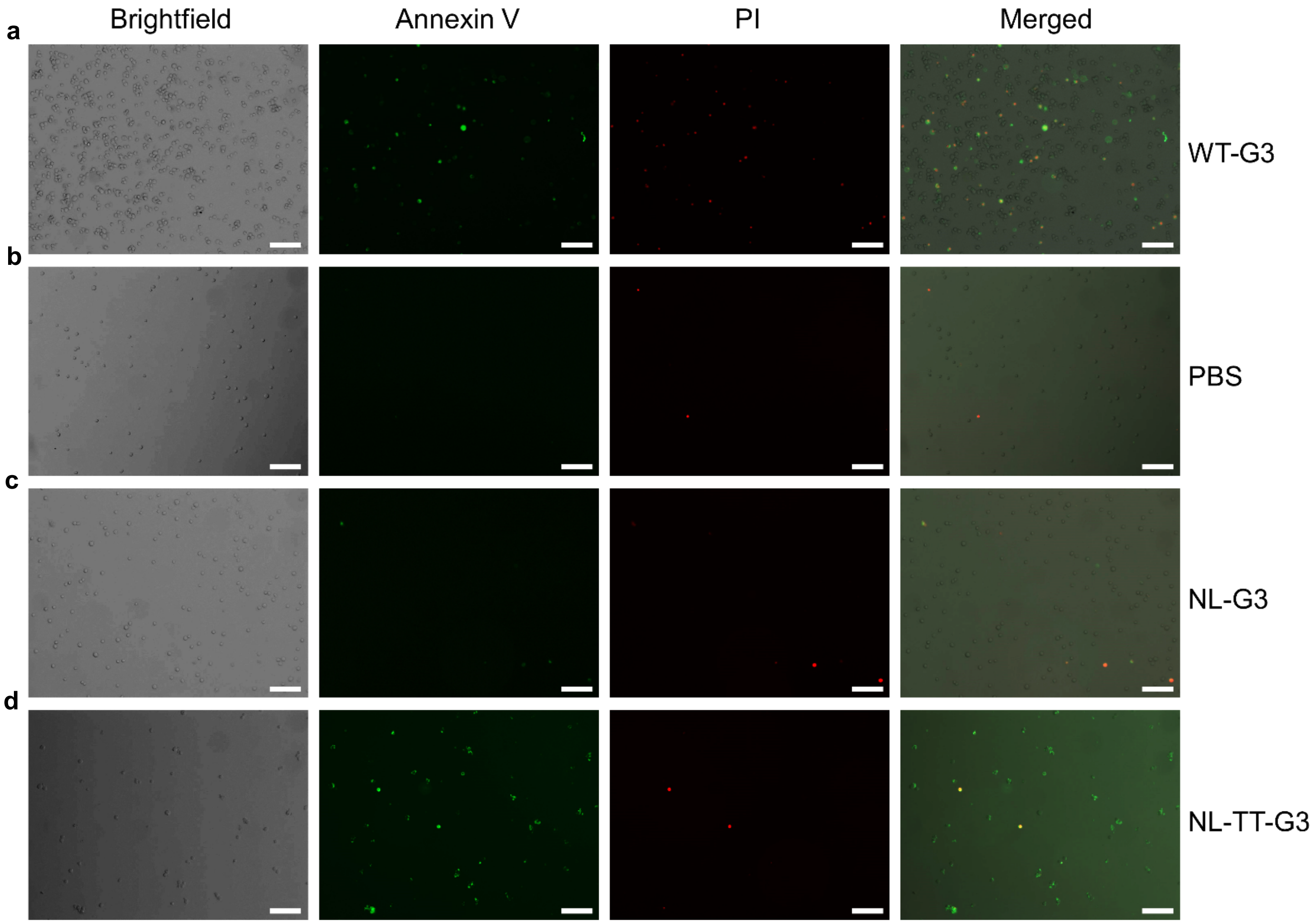

Supplementary Figure 7. Phosphatidylserine exposure and membrane permeability of Jurkat T cells treated with monomeric G3 fusion proteins and trimeric nanoassemblies. Phosphatidylserine exposure was determined via Annexin V staining and membrane permeability was determined via propidium iodide (PI) after Jurkat T cells were treated with **a** wild-type galectin-3 (WT-G3), **b** PBS, **c** NL-G3, or **d** NL-TT-G3. Scale bar = 100 μm.

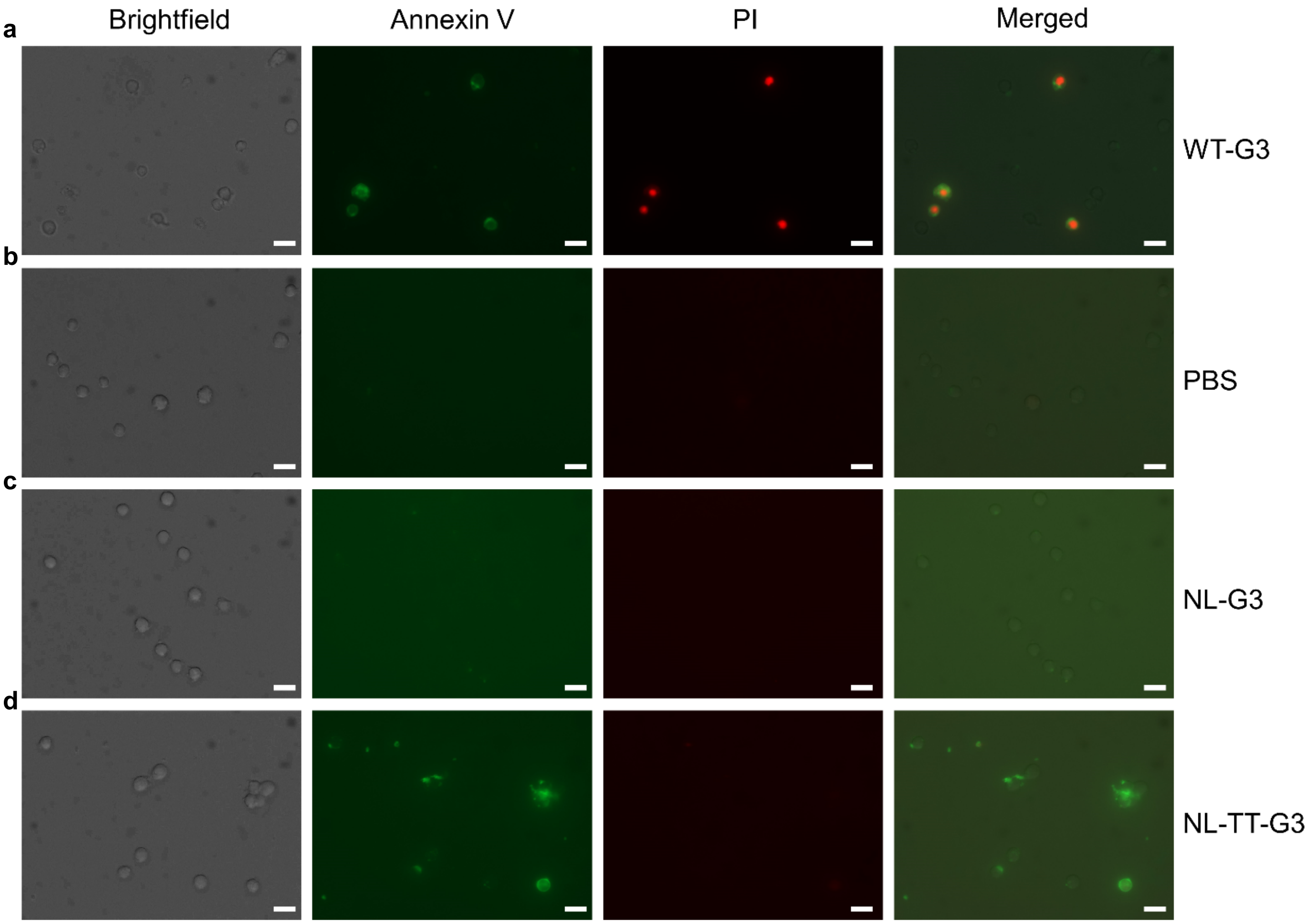

Supplementary Figure 8. Phosphatidylserine exposure and membrane permeability of Jurkat T cells treated with monomeric G3 fusion proteins and trimeric nanoassemblies. Phosphatidylserine exposure was determined via Annexin V staining and membrane permeability was determined via propidium iodide (PI) after Jurkat T cells were treated with **a** wild-type galectin-3 (WT-G3), **b** PBS, **c** NL-G3, or **d** NL-TT-G3. Scale bar = 20 µm.

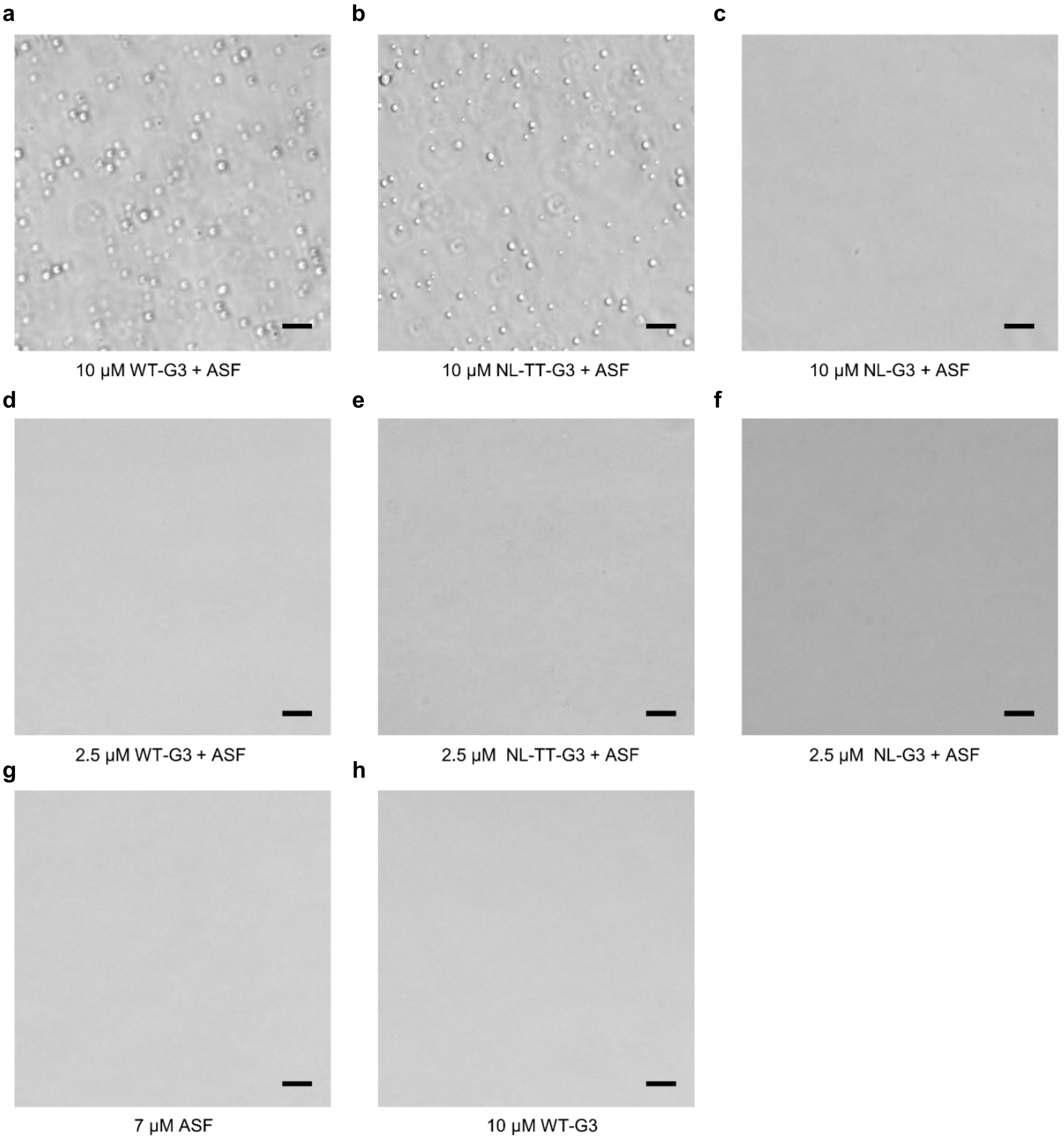

Supplementary Figure 9. Brightfield micrographs of insoluble aggregates formed by asialofetuin (ASF) in the presence of wild-type galectin-3 (WT-G3) and trimeric nanoassemblies in PBS at high concentrations ([G3] = 10 μM). 7 μM ASF in the presence of **a** 10 μM WT-G3, **b** 10 μM NL-TT-G3, **c** 10 μM NL-G3, **d** 2.5 μM WT-G3, **e** 2.5 μM NL-TT-G3, or **f** 2.5 μM NL-G3. **g** 7 μM ASF alone and **h** 10 μM WT-G3 alone. Scale bar = 20 μm.

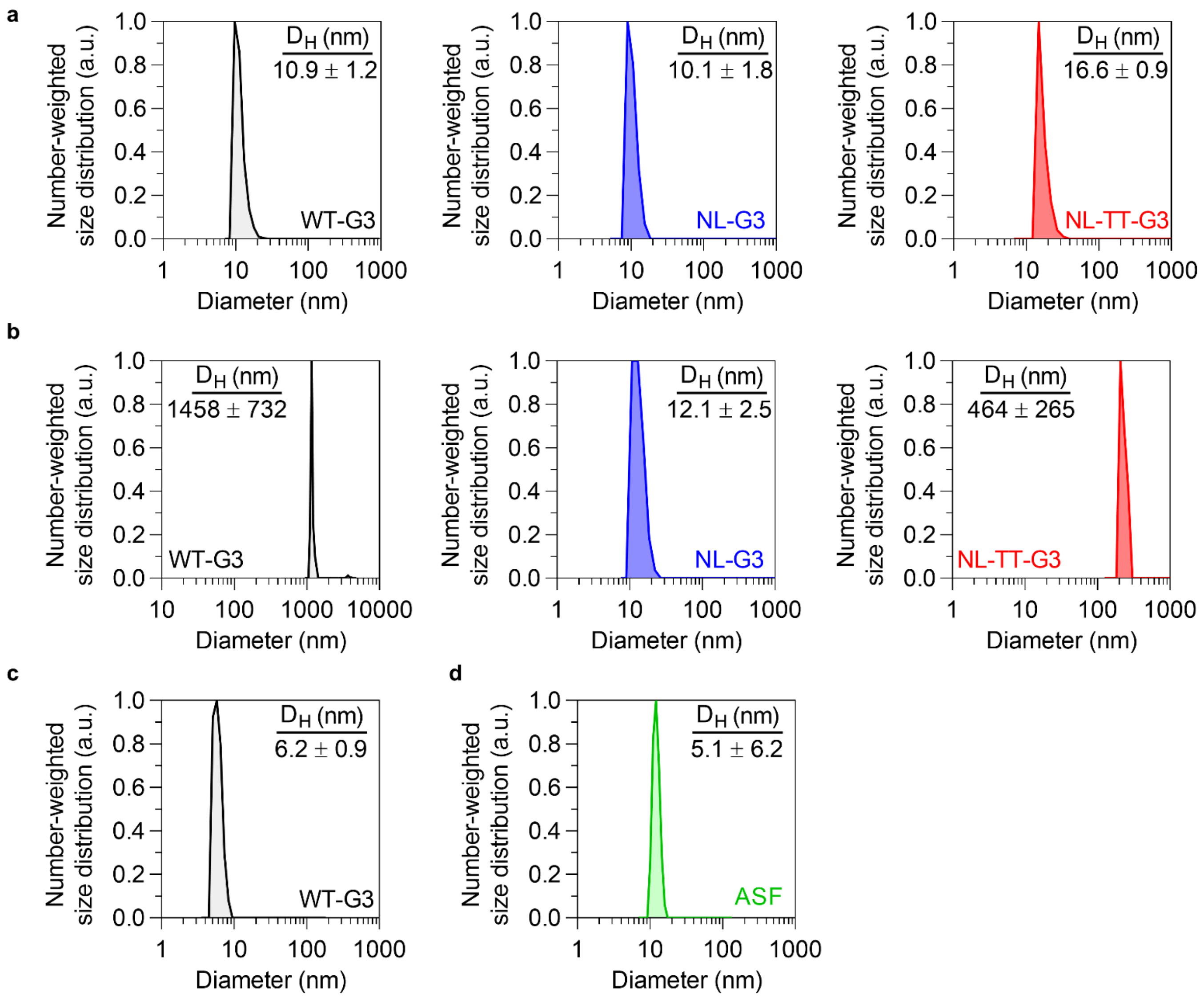

Supplementary Figure 10. Average hydrodynamic diameter of insoluble aggregates formed by asialofetuin (ASF) in the presence of wild-type galectin-3 (WT-G3) and trimeric nanoassemblies in PBS at high concentrations ([G3] = 10 µM), as determined via dynamic light scattering. 7 µM ASF in the presence of **a** 2.5 µM WT-G3, NL-G3, or NL-TT-G3. 7 µM ASF in the presence of **b** 10 µM WT-G3, NL-G3, or NL-TT-G3. **c** 10 µM WT-G3 alone and **d** 7 µM ASF alone. N = 3, mean ± s.d. WT-G3 is presented as a black trace, NL-G3 is presented as a blue trace, NL-TT-G3 is presented as a red trace, and ASF is presented as a green trace.

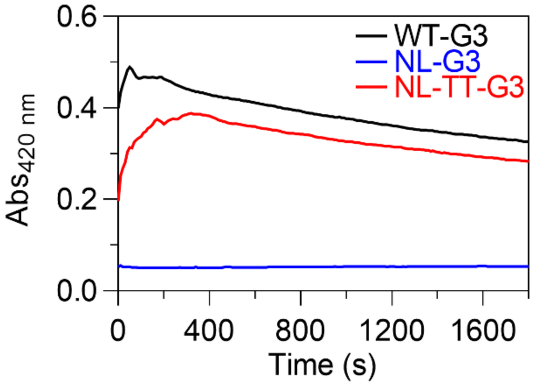

Supplementary Figure 11. Time-course curve for G3-mediated formation of insoluble asialofetuin (ASF) aggregates. 7 μM ASF was added to WT-G3 (black trace), NL-G3 (blue trace), or NL-TT-G3 (red trace) at high concentrations ([G3] = 10 μM) and aggregate formation was determined by light scattering (λ = 420 nm).

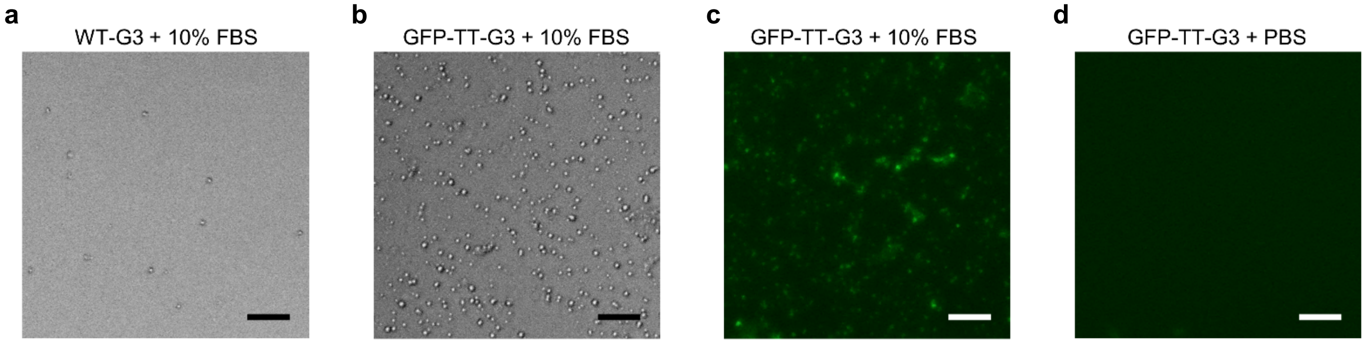

Supplementary Figure 12. Brightfield and fluorescent micrographs of insoluble aggregates formed in 10% fetal bovine serum (FBS) in the presence of WT-G3 or GFP-TT-G3. **a** 10 μM WT-G3 added to FBS. GFP-TT-G3 ([G3] = 10 μM) added to **b-c** FBS or **d** PBS. Scale bar = 20 μm.

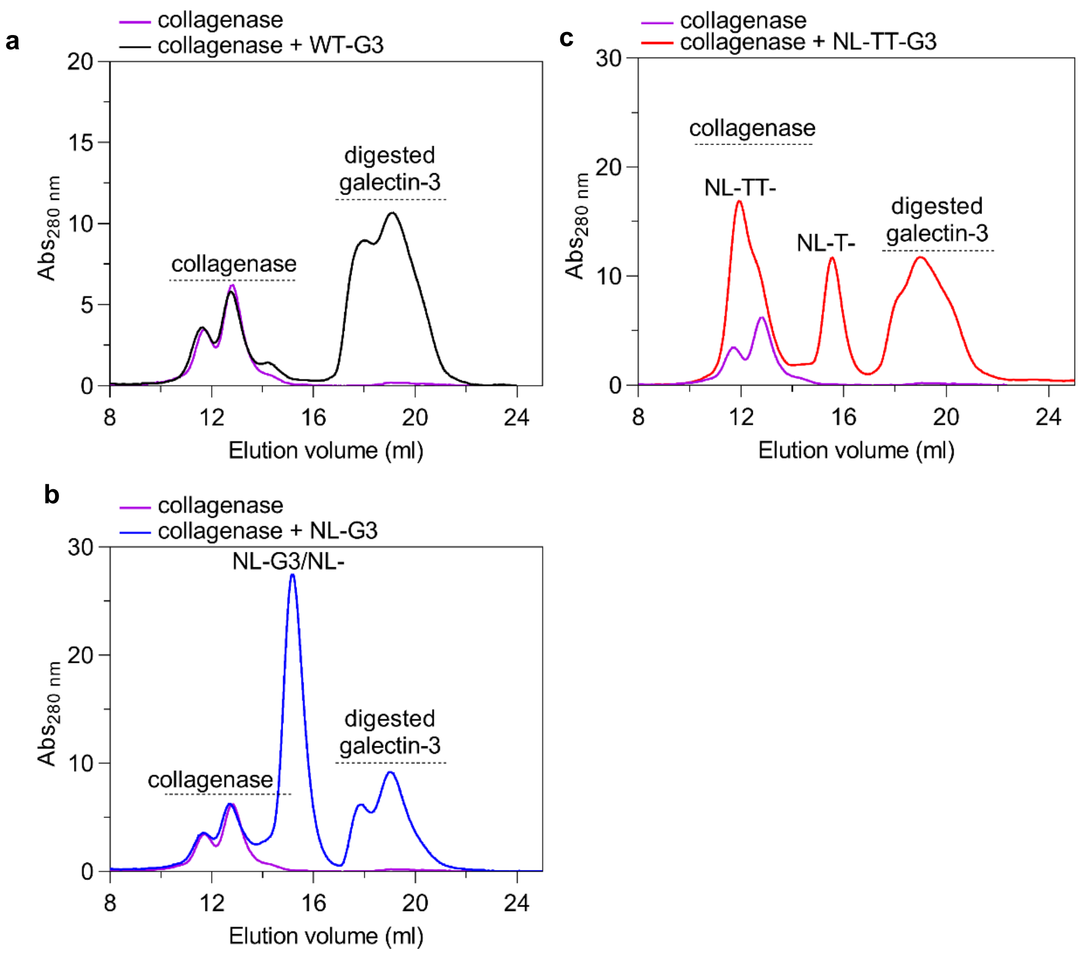

Supplementary Figure 13. Size-exclusion chromatography of monomeric G3 fusion proteins and trimeric nanoassemblies digested with collagenase. **a** Wild-type galectin-3 (WT-G3, black trace), **b** NL-G3 (blue trace), and **c** NL-TT-G3 (red trace) fully or partially digested by collagenase (purple trace in **a**-**c**). In **b,** both undigested NL-G3 and digested NL-G3 (i.e., NL-) is present under a broad peak. In **c**, digested NL-TT-G3 that remained assembled (i.e., NL-TT-) overlaps with the collagenase peaks, while digested and unassembled NL-TT-G3 (i.e., NL-T-) eluted later.

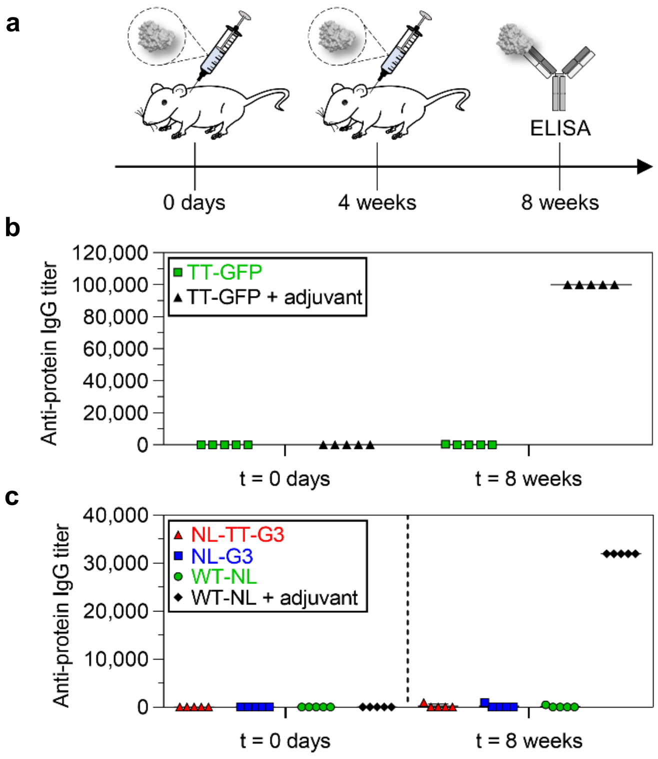

Supplementary Figure 14. Total C57BL/6 serum IgG reactive against monomeric G3 fusion proteins and trimeric nanoassemblies after subcutaneous scruff injection of proteins in PBS or in TiterMax™ adjuvant emulsion. **a** Timeline for initial protein injection (0 days), boost injection (4 weeks), and analysis of anti-protein IgG antibody response via ELISA (8 weeks). **b** Anti-protein IgG titer for mice injected with TT-GFP in PBS (green squares) or TT-GFP in adjuvant emulsion (TT-GFP + adjuvant, black triangles). **c** Anti-protein IgG titer for mice injected with NL-TT-G3 in PBS (red triangles), NL-G3 in PBS (blue squares), wild-type NanoLuc™ in PBS (WT-NL, green circles), and wild-type NanoLuc™ in adjuvant emulsion (WT-NL + adjuvant, black diamonds). In **b** and **c**, 0 days represents serum from naïve, untreated mice, which was collected before the first protein injection. N = 5, mean ± s.d.

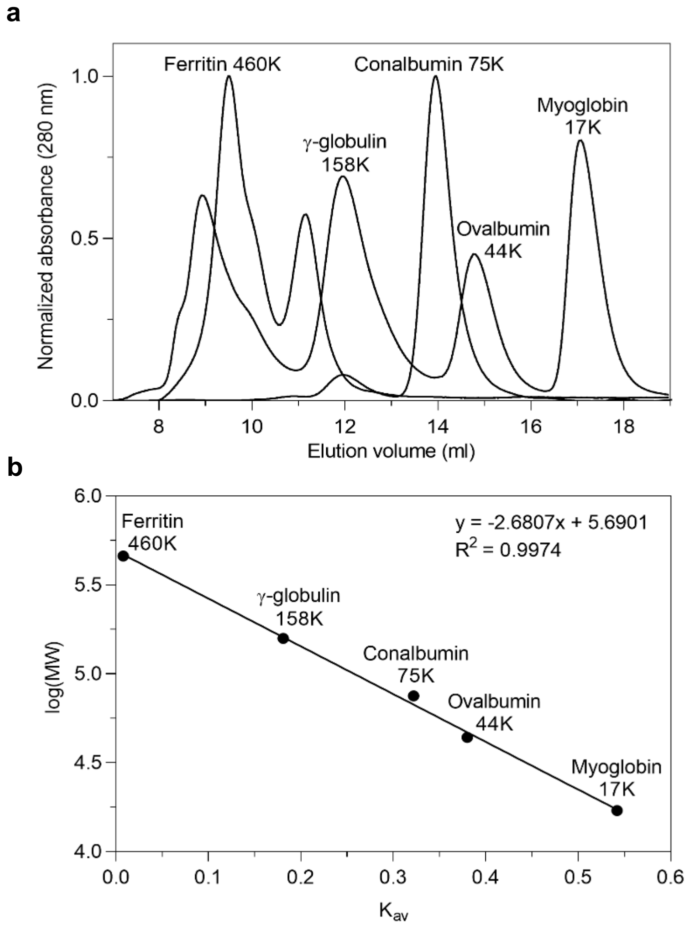

Supplementary Figure 15. Size-exclusion chromatography (SEC) calibration. **a** Overlapped traces of protein standards on a Superdex 200 10/300 GL column. **b** SEC standard curve plotted as log(MW) vs partition coefficient (K_{av}). K_{av} is equal to the protein elution volume (V_e) minus the void volume (V_o, 9.39 mL) and divided by the total column volume (V_t, 23.56 mL) minus the void volume.

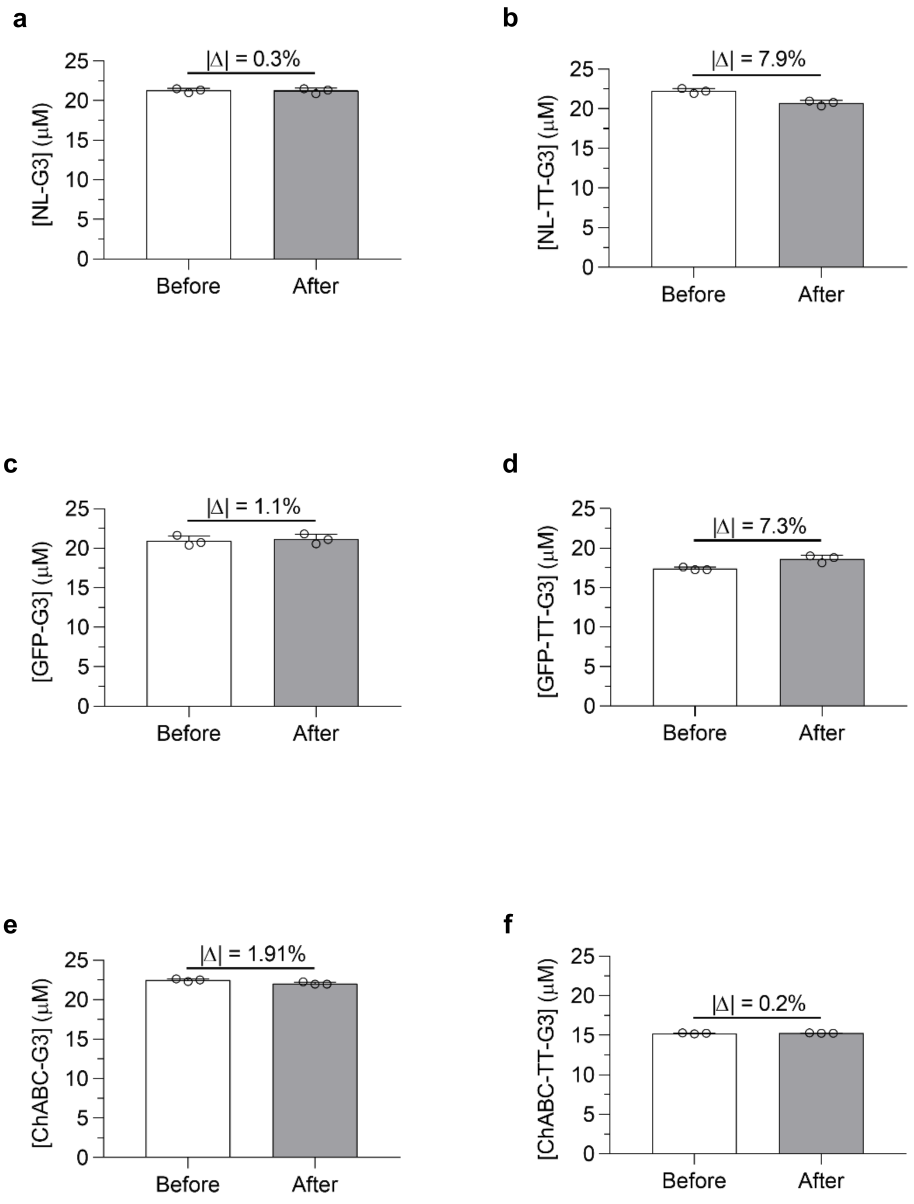

Supplementary Figure 16. Molar concentration of monomeric G3 fusion proteins and trimeric nanoassemblies measured before (white bar) and after (gray bar) filtration with a 0.2 micron syringe filter for dynamic light scattering experiments. **a** NL-G3, **b** NL-TT-G3, **c** GFP-G3, **d** GFP-TT-G3, **e** ChABC-G3, and **f** ChABC-TT-G3. Δ = percent change in molar concentration. N = 3, mean ± s.d. Data points at or above baseline signal are shown as open circles.

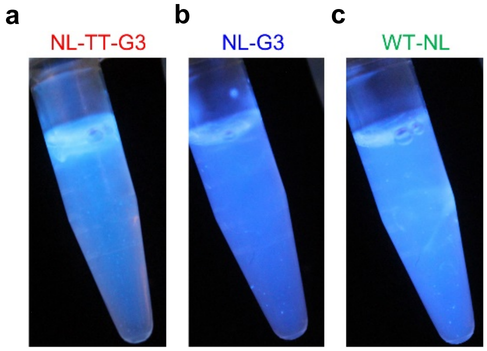

Supplementary Figure 17. Digital photographic images of blue luminescence emitted by monomeric G3 fusion proteins and trimeric nanoassemblies. Luminescence emitted by **a** NL-TT-G3, **b** NL-G3, and **c** wild-type NanoLuc™ (WT-NL).

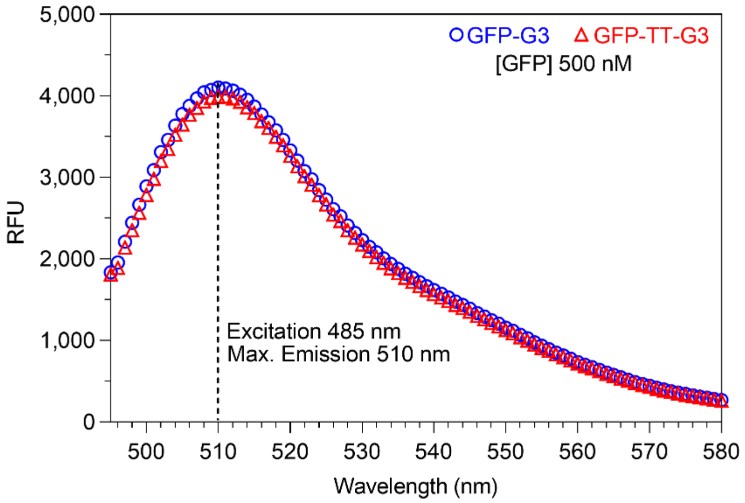

Supplementary Figure 18. Fluorescence spectra of monomeric G3 fusion proteins and trimeric nanoassemblies. Emission spectra of GFP-G3 (blue circles) and GFP-TT-G3 (red triangles) ([GFP] = 500 nM) at excitation = 485 nm (superfolder GFP). N = 3, mean ± s.d.

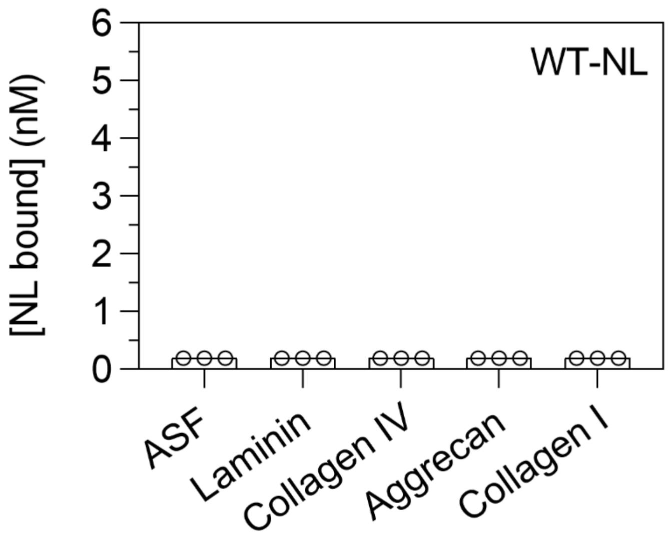

Supplementary Figure 19. Baseline signal produced by non-specific binding of wild-type NanoLuc™ (WT-NL) to various glycoproteins and proteoglycans. N = 3, mean ± s.d. Data points at baseline signal are shown as open circles.

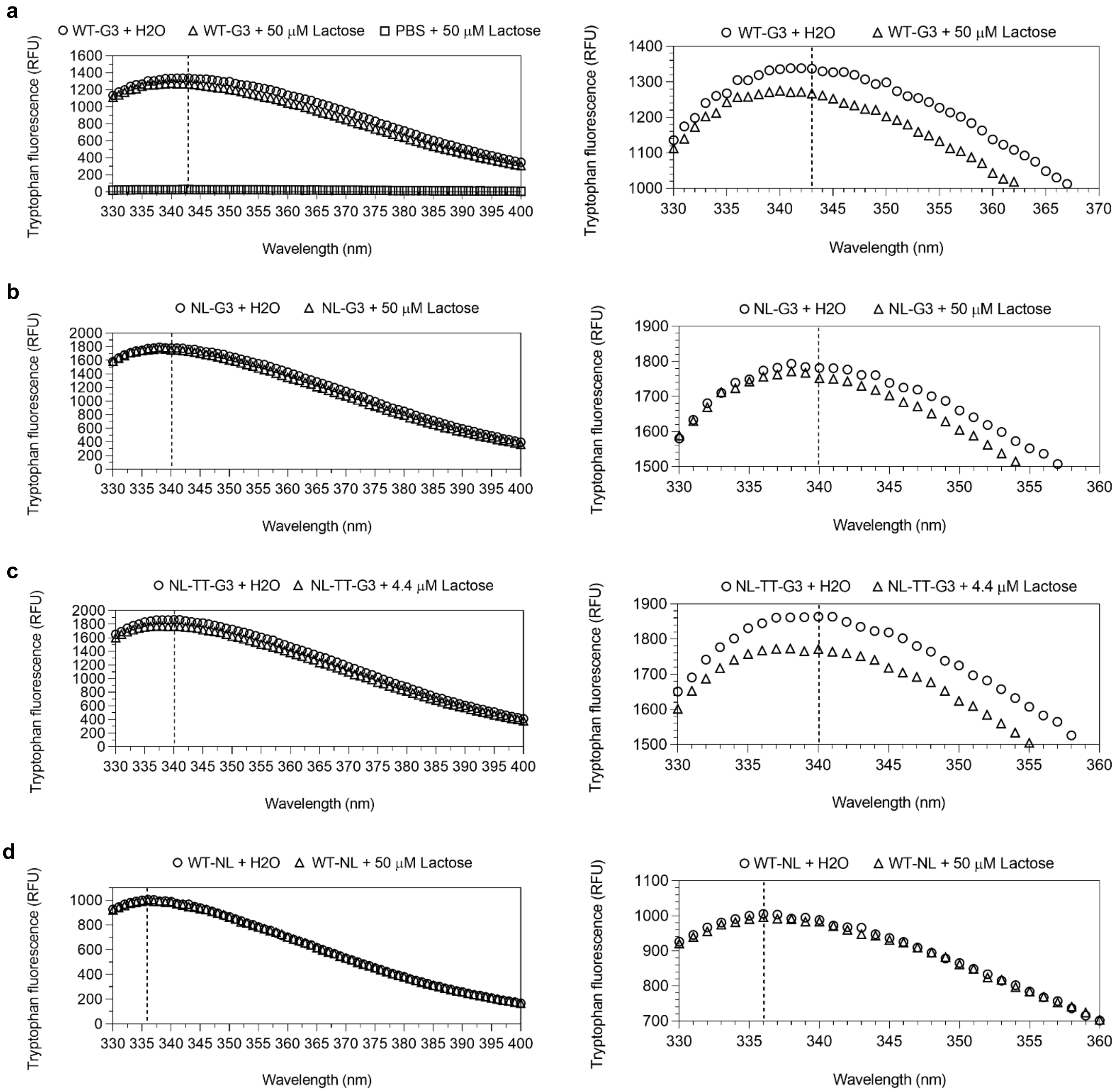

Supplementary Figure 20. Representative tryptophan fluorescence emission spectra of monomeric G3 fusion proteins and trimeric nanoassemblies in PBS spiked with water (negative control, circles) or soluble lactose in water (triangles). **a** Wild-type galectin-3 (WT-G3), **b** NL-G3, **c** NL-TT-G3, and **d** wild-type NanoLuc™ (WT-NL). Excitation = 280 nm. N = 3, mean ± s.d.

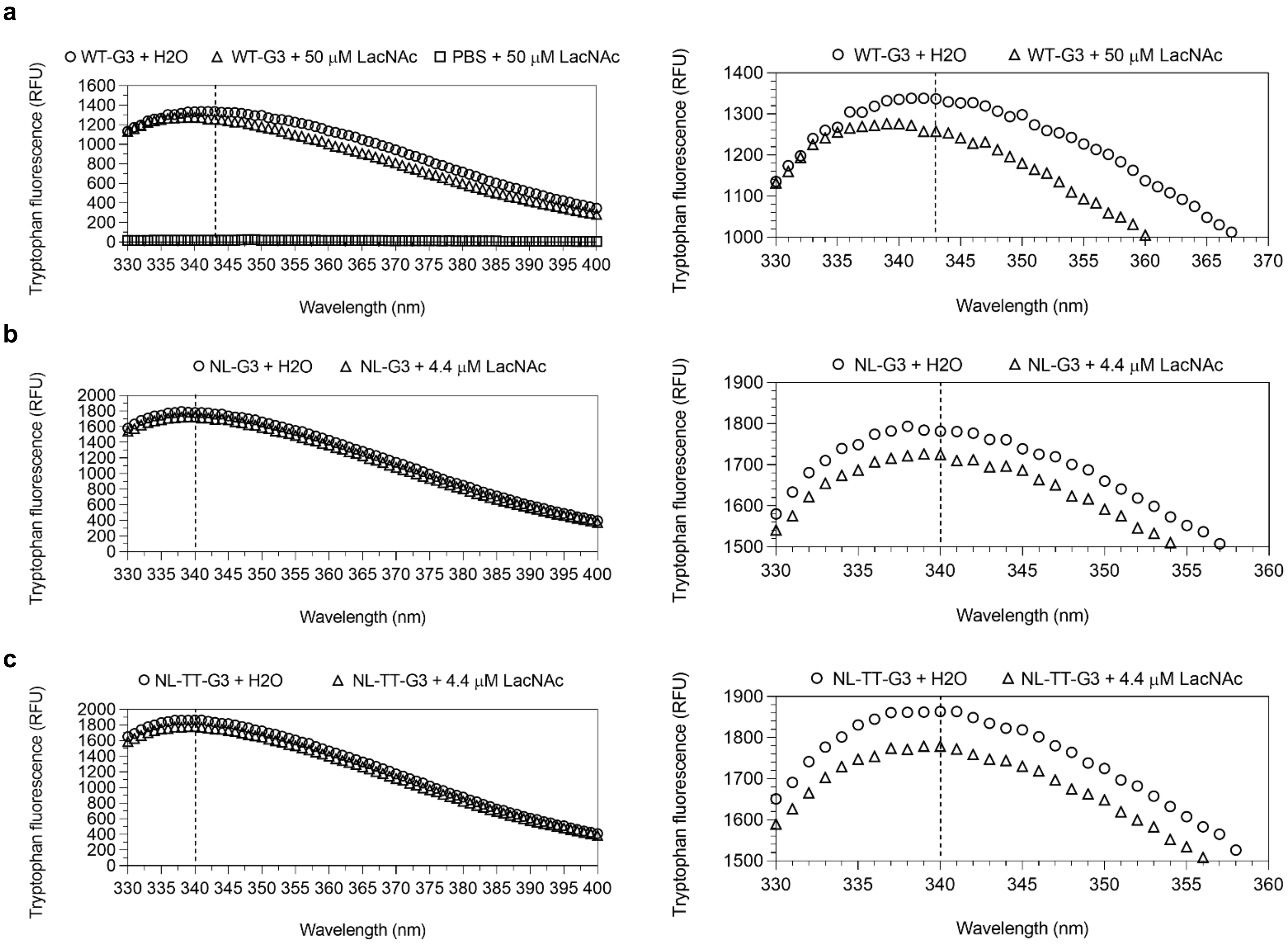

Supplementary Figure 21. Representative tryptophan fluorescence emission spectra of monomeric G3 fusion proteins and trimeric nanoassemblies in PBS spiked with water (negative control, circles) or soluble LacNAc in water (triangles). **a** Wild-type galectin-3 (WT-G3), **b** NL-G3, and **c** NL-TT-G3. Excitation = 280 nm. N = 3, mean ± s.d.

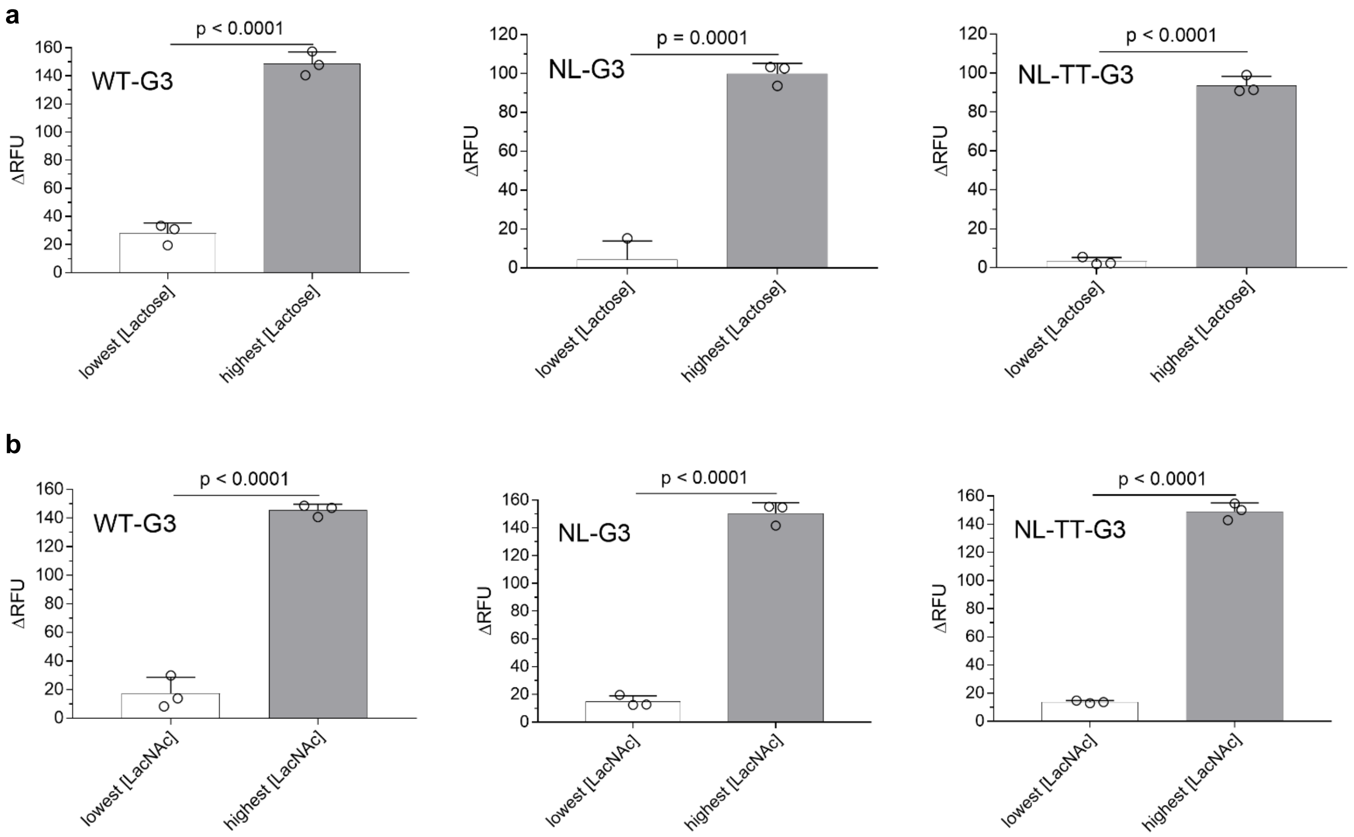

Supplementary Figure 22. Change in fluorescence signal (ΔRFU) for wild-type galectin-3 (WT-G3), NL-G3, and NL-TT-G3 in solution with the lowest (white bar) and highest (gray bar) concentration of soluble carbohydrate used in tryptophan fluorescence quenching experiments. **a** ΔRFU for lowest and highest [Lactose] extracted from supplementary figure 7. **b** ΔRFU for lowest and highest [LacNAc] extracted from figure 3f. P < 0.0001 demonstrates that ΔRFU at the highest carbohydrate concentration tested was significantly greater than ΔRFU at the lowest carbohydrate concentration tested. N = 3, mean ± s.d. Data points at or above baseline signal are shown as open circles.

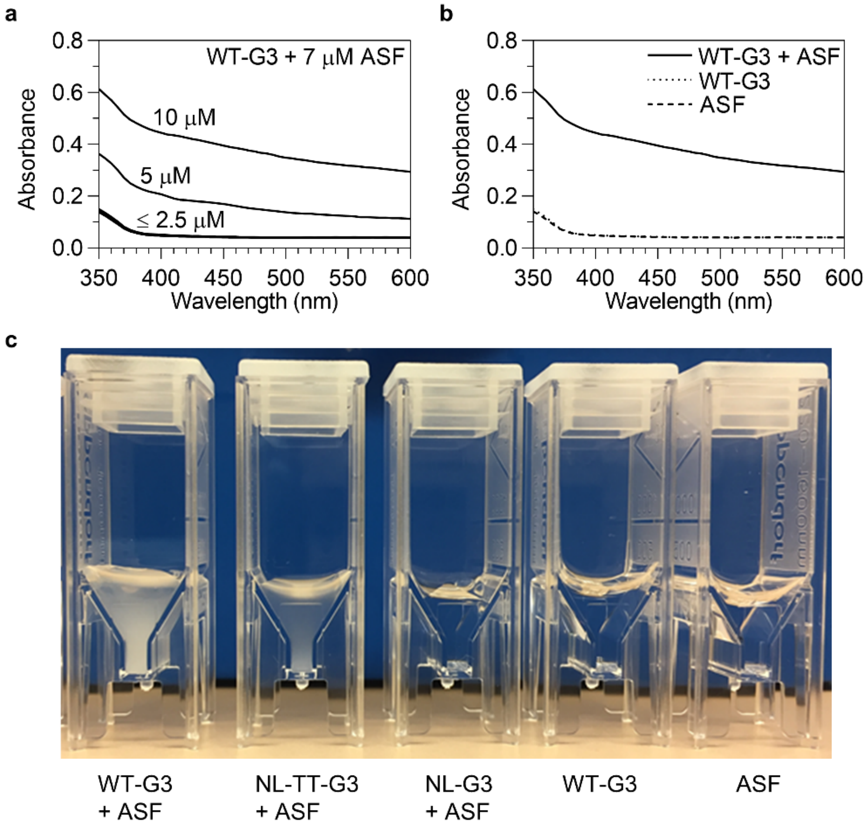

Supplementary Figure 23. Analysis of insoluble aggregates formed by asialofetuin (ASF) in the presence of WT-G3, NL-G3, and NL-TT-G3. **a** Absorbance of insoluble aggregates at different concentrations of WT-G3 added to ASF. **b** Absorbance spectra of 10 μM WT-G3, 7 μM ASF, and insoluble aggregates formed by combining both proteins in solution. **c** Qualitative digital photographic images of proteins mixed with ASF or proteins alone in PBS. N = 3, mean ± s.d. for **a** and **b**.

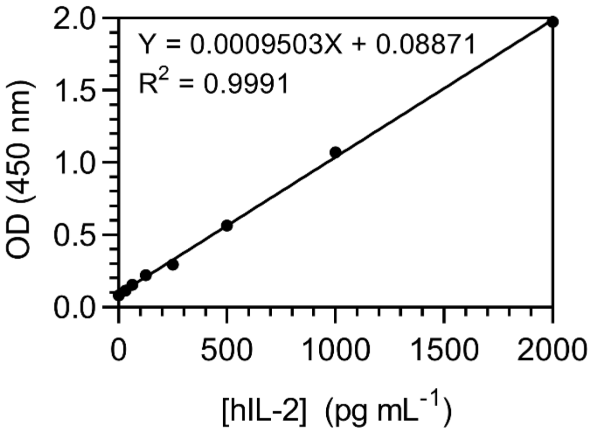

Supplementary Figure 24. Standard curve of absorbance (450 nm) vs human IL-2 (hIL-2) concentration for the hIL-2 ELISA used to determine the concentration of hIL-2 secreted by Jurkat T cells treated with WT-G3, NL-G3, or NL-TT-G3.

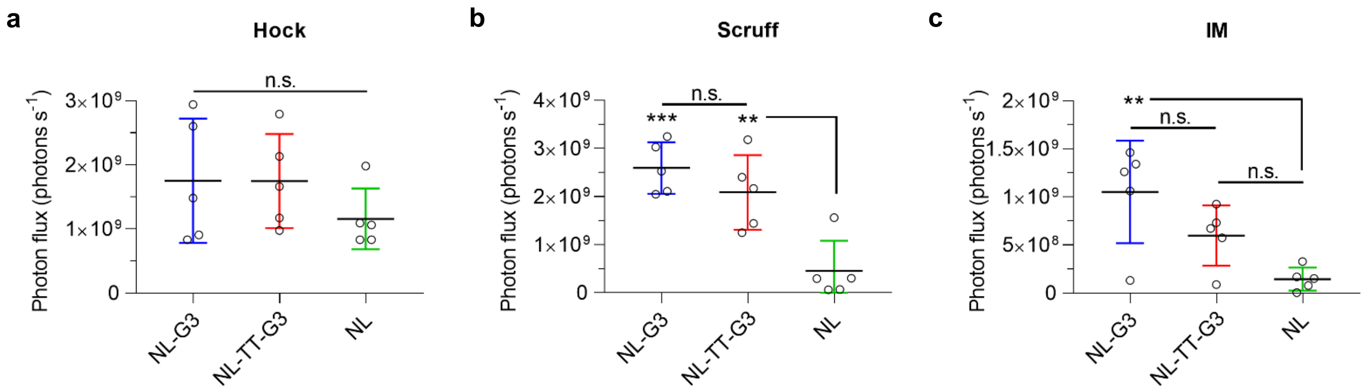

Supplementary Figure 25. Bioluminescence output in terms of photon flux (photon s^{-1}) for NL, NL-G3, and NL-TT-G3 at each injection site in female C57BL/6 mice at day 0. **a** hock, **b** scruff, and **c** caudal thigh muscle (intramuscular, IM). N = 5, mean ± s.d., n.s. is no significant difference, **p < 0.01, ***p < 0.001, ANOVA with Tukey's post-hoc. Data points at or above baseline signal are shown as open circles. NL-G3 is presented as blue error bars, NL-TT-G3 is presented as red error bars, and WT-NL is presented as green error bars.

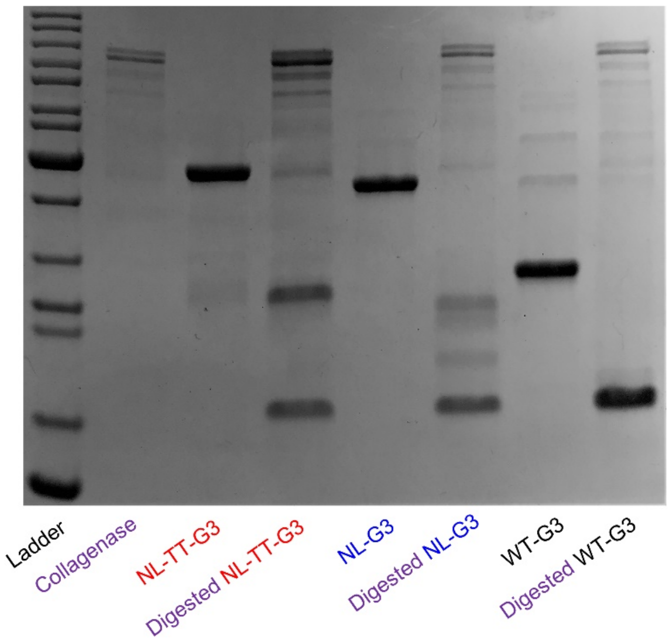

Supplementary Figure 26. Original SDS-PAGE gel of WT-G3, NL-G3, and NL-TT-G3 before and after treatment with collagenase. This is the same gel as is shown in figure 6d, uncropped and without adjusting brightness/contrast to improve printing quality.

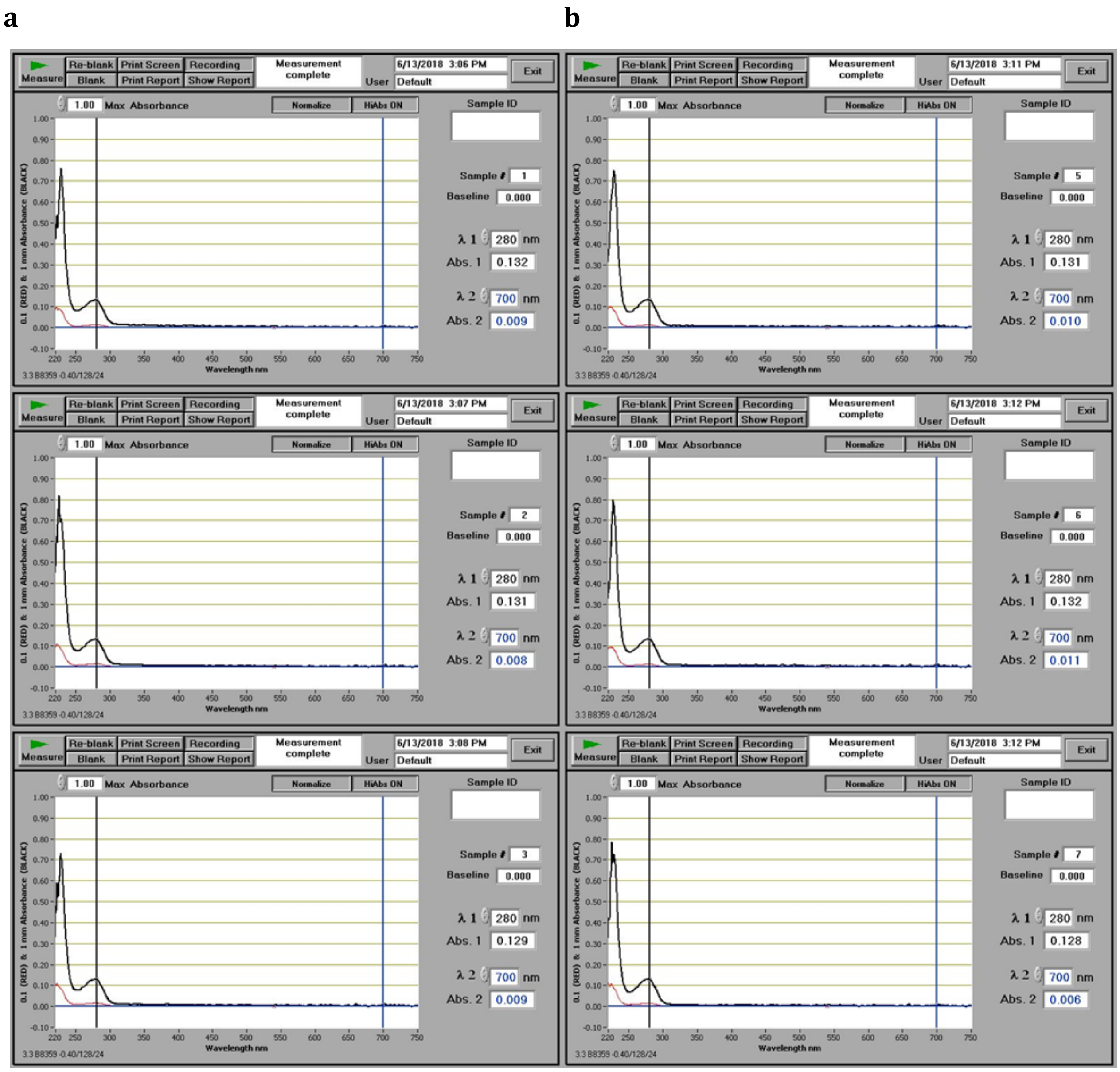

Supplementary Figure 27. NanoDrop Spectra of 0.2 micron filtered NL-G3 in PBS. **a** before filtration and **b** after. Data in columns are technical replicates of **a** or **b**.

a b

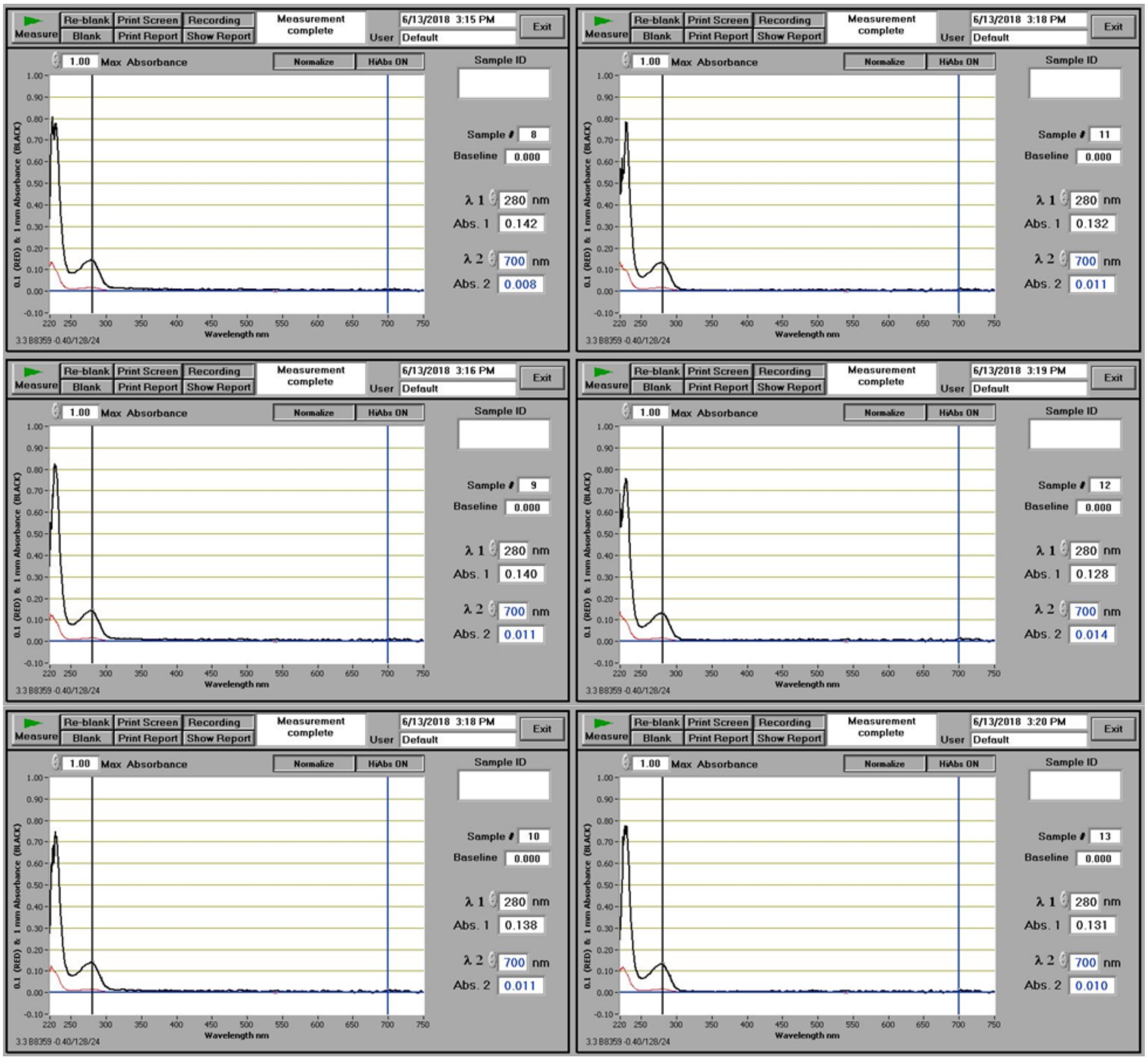

Supplementary Figure 28. NanoDrop Spectra of 0.2 micron filtered NL-TT-G3 in PBS. **a** before filtration and **b** after. Data in columns are technical replicates of **a** or **b**.

a **b**

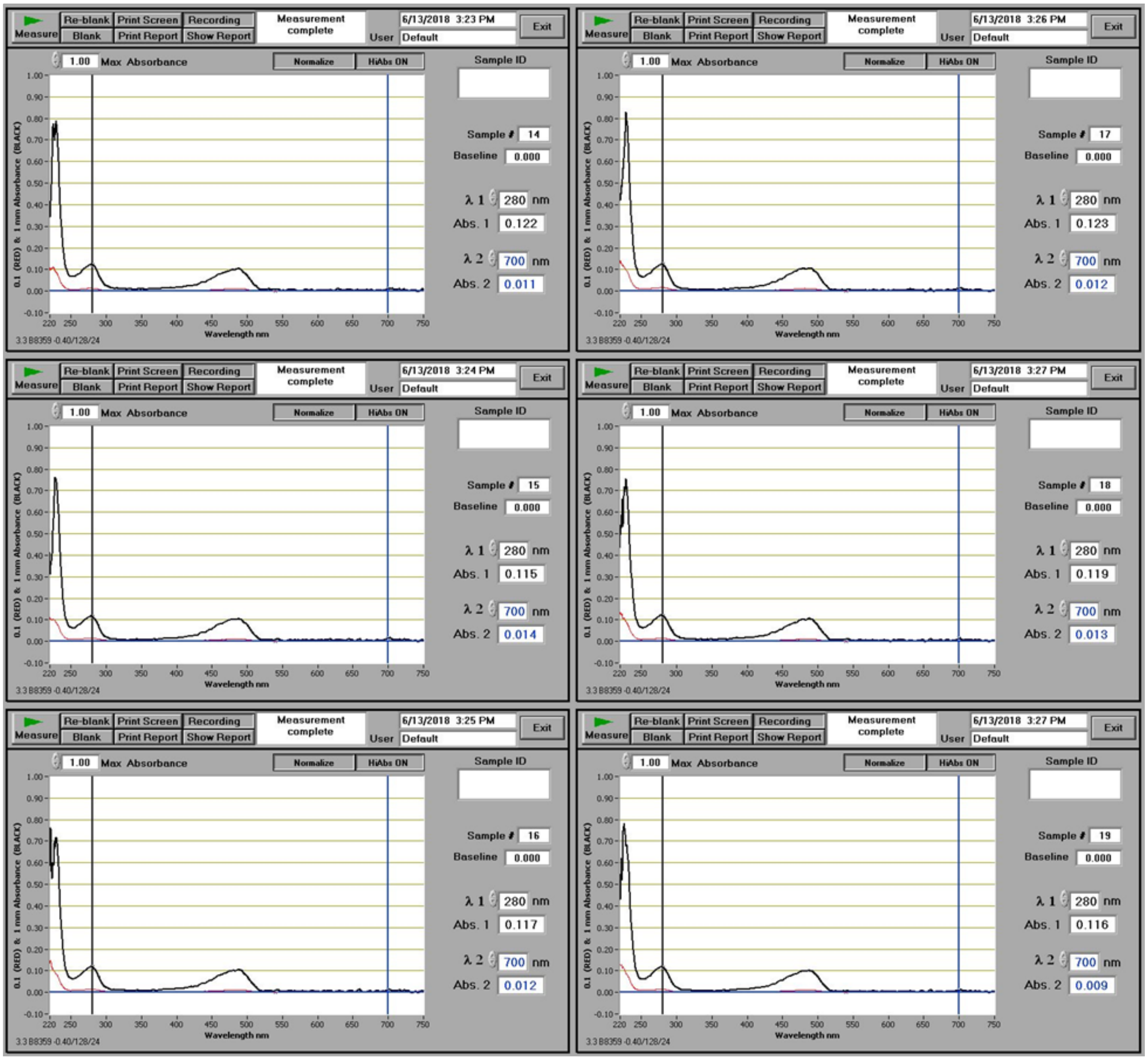

Supplementary Figure 29. NanoDrop Spectra of 0.2 micron filtered GFP-G3 in PBS. **a** before filtration and **b** after. Data in columns are technical replicates of **a** or **b**.

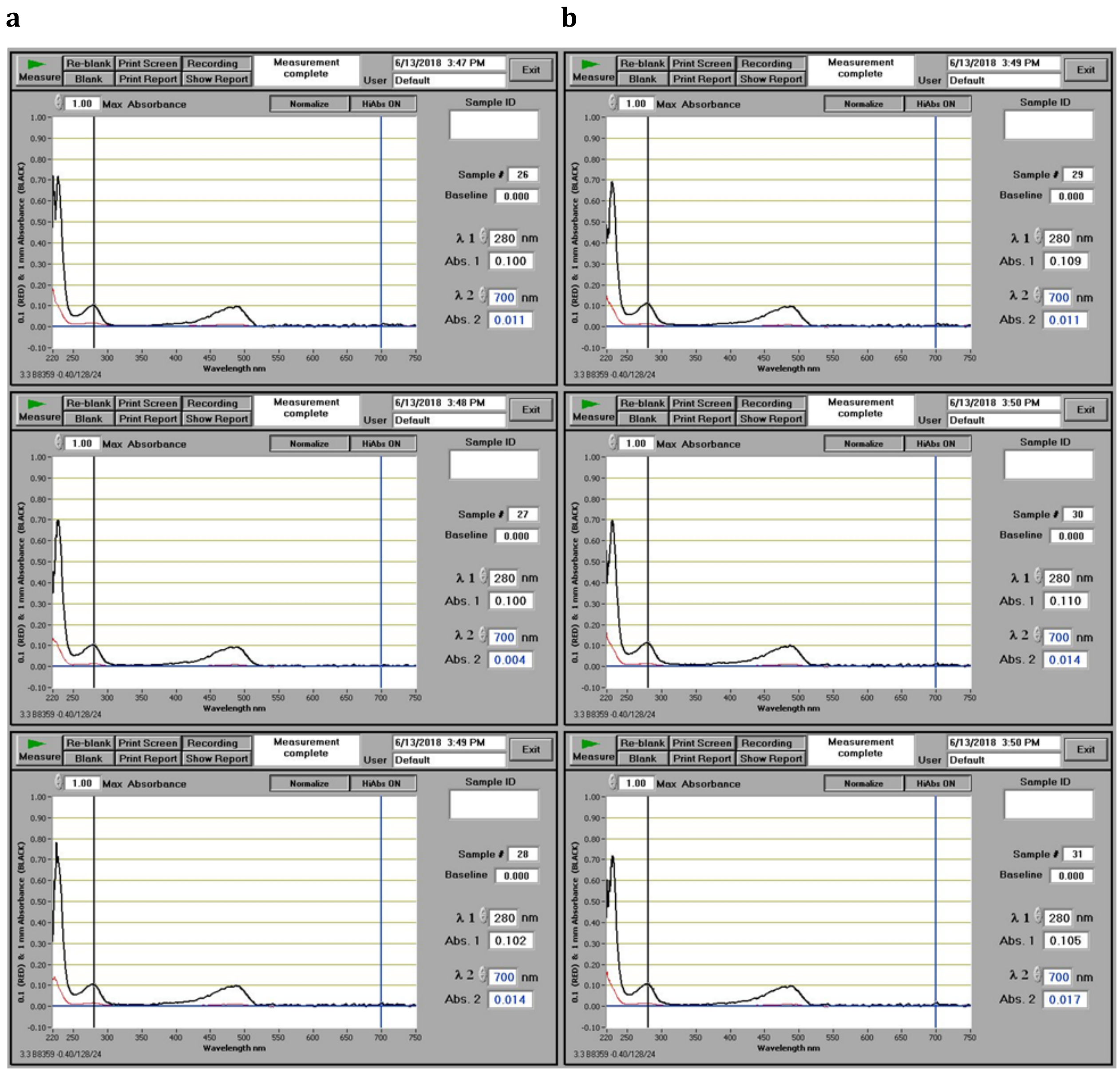

Supplementary Figure 30. NanoDrop Spectra of 0.2 micron filtered GFP-TT-G3 in PBS. **a** before filtration and **b** after. Data in columns are technical replicates of **a** or **b**.

a **b**

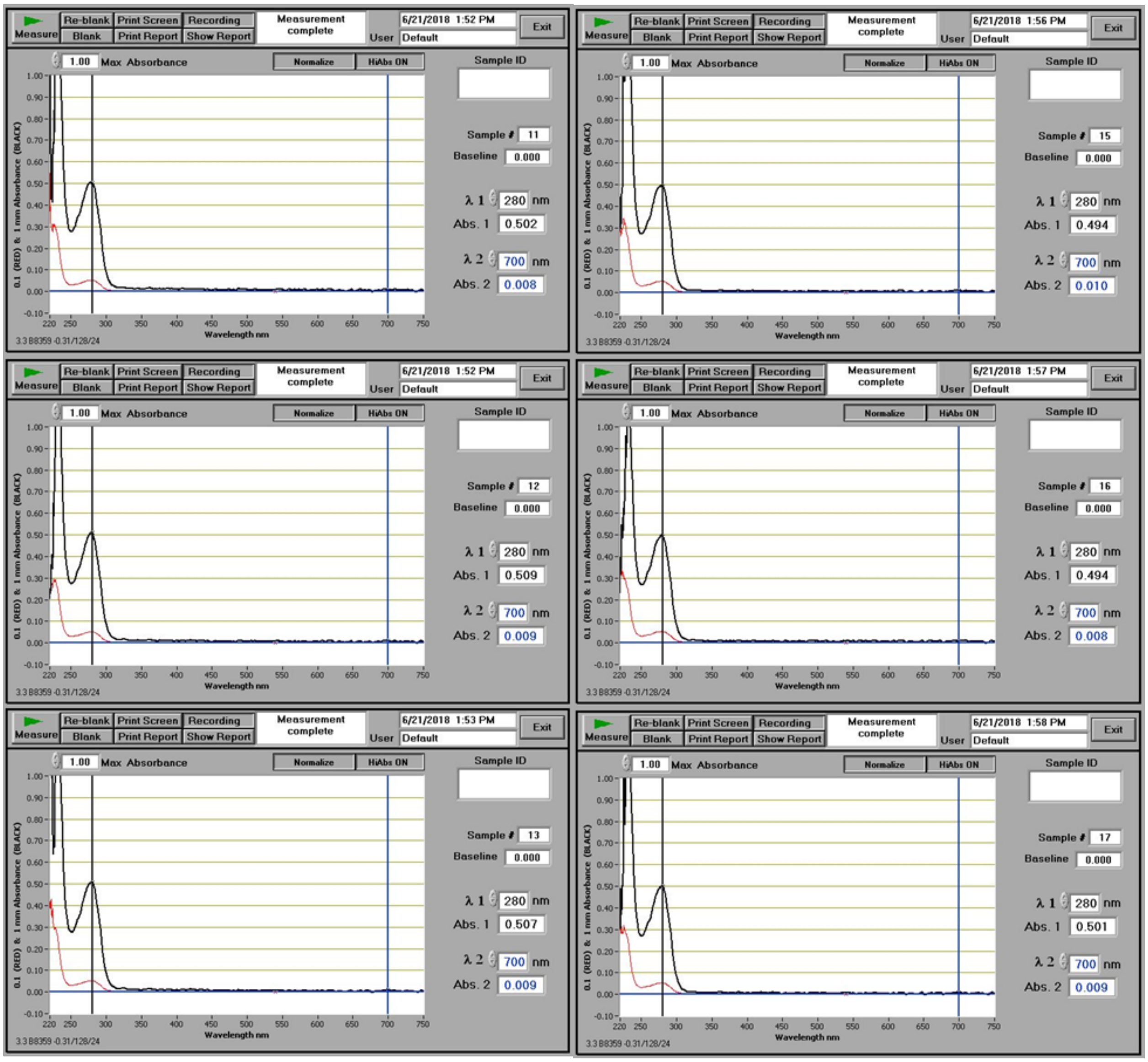

Supplementary Figure 31. NanoDrop Spectra of 0.2 micron filtered ChABC-G3 in PBS. **a** before filtration and **b** after. Data in columns are technical replicates of **a** or **b**.

a **b**

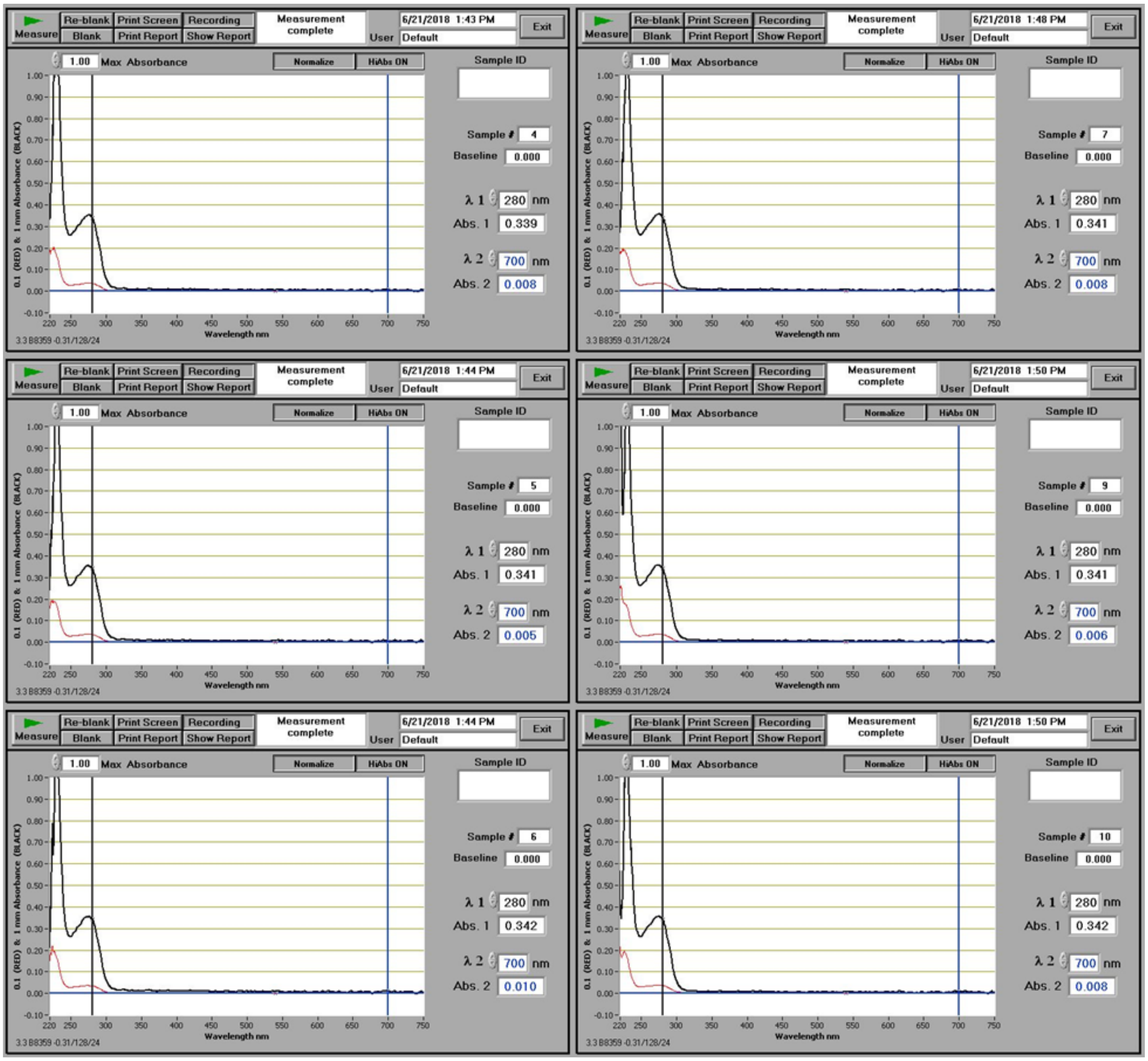

Supplementary Figure 32. NanoDrop Spectra of 0.2 micron filtered ChABC-TT-G3 in PBS. **a** before filtration and **b** after. Data in columns are technical replicates of **a** or **b**.

a **b**

Sample ID NL-G3 (Combined)
Date - Time May 14, 2018 12:07:15
Operator ID SF
Elapsed Time 00:05:00
Mean Diam. 3.1 nm
Rel. Var. 0.032
Skew 6.041

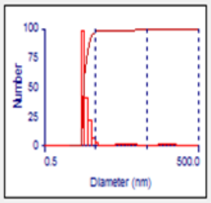

d(nm)	G(d)	C(d)	d(nm)	G(d)	C(d)	d(nm)	G(d)	C(d)
1.7	0	0	9.6	0	100	53.5	0	100
2.0	0	0	11.2	0	100	62.6	0	100
2.4	0	0	13.1	0	100	73.1	0	100
2.8	100	59	15.4	0	100	85.5	0	100
3.2	41	83	17.9	0	100	100.0	0	100
3.8	21	95	21.0	0	100	116.8	0	100
4.4	6	99	24.5	0	100	136.6	0	100
5.1	2	100	28.7	0	100	159.7	0	100
6.0	0	100	33.5	0	100	186.7	0	100
7.0	0	100	39.2	0	100	218.2	0	100
8.2	0	100	45.8	0	100	255.1	0	100

Number

Sample ID NL-G3 (Combined)
Date - Time May 14, 2018 12:07:15
Operator ID SF
Elapsed Time 00:05:00
Mean Diam. 4.1 nm
Rel. Var. 1.239
Skew 15.643

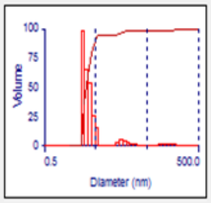

d(nm)	G(d)	C(d)	d(nm)	G(d)	C(d)	d(nm)	G(d)	C(d)
1.7	0	0	9.6	0	96	53.5	0	100
2.0	0	0	11.2	0	96	62.6	0	100
2.4	0	0	13.1	2	96	73.1	0	100
2.8	100	37	15.4	4	98	85.5	0	100
3.2	65	61	17.9	3	99	100.0	0	100
3.8	53	81	21.0	2	100	116.8	0	100
4.4	26	90	24.5	0	100	136.6	0	100
5.1	15	96	28.7	0	100	159.7	0	100
6.0	0	96	33.5	0	100	186.7	0	100
7.0	0	96	39.2	0	100	218.2	0	100
8.2	0	96	45.8	0	100	255.1	0	100

Volume

Sample ID NL-G3 run 2 (Combined)
Date - Time May 14, 2018 12:27:25
Operator ID SF
Elapsed Time 00:05:00
Mean Diam. 5.1 nm
Rel. Var. 0.022
Skew 4.753

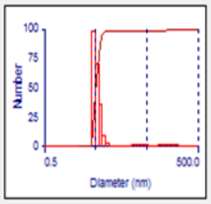

d(nm)	G(d)	C(d)	d(nm)	G(d)	C(d)	d(nm)	G(d)	C(d)
2.5	0	0	12.7	0	100	64.7	0	100
2.9	0	0	14.8	0	100	75.0	0	100
3.4	0	0	17.1	0	100	87.0	0	100
3.9	0	0	19.8	0	100	100.8	0	100
4.5	100	46	23.0	0	100	116.9	0	100
5.3	71	79	26.7	0	100	135.5	0	100
6.1	36	96	30.9	0	100	157.1	0	100
7.1	8	99	35.8	0	100	182.1	0	100
8.2	1	100	41.5	0	100	211.1	0	100
9.5	0	100	48.2	0	100	244.7	0	100
11.0	0	100	55.8	0	100	283.6	0	100

Number

Sample ID NL-G3 run 2 (Combined)
Date - Time May 14, 2018 12:27:25
Operator ID SF
Elapsed Time 00:05:00
Mean Diam. 6.1 nm
Rel. Var. 0.847
Skew 15.068

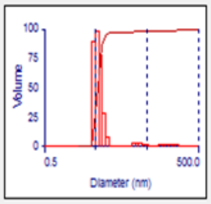

d(nm)	G(d)	C(d)	d(nm)	G(d)	C(d)	d(nm)	G(d)	C(d)
2.5	0	0	12.7	0	98	64.7	0	100
2.9	0	0	14.8	0	98	75.0	0	100
3.4	0	0	17.1	0	98	87.0	0	100
3.9	0	0	19.8	0	98	100.8	0	100
4.5	90	29	23.0	0	98	116.9	0	100
5.3	100	62	26.7	2	99	135.5	0	100
6.1	78	87	30.9	2	99	157.1	0	100
7.1	28	96	35.8	1	100	182.1	0	100
8.2	7	98	41.5	0	100	211.1	0	100
9.5	0	98	48.2	0	100	244.7	0	100
11.0	0	98	55.8	0	100	283.6	0	100

Volume

Sample ID NL-G3 run 3 (Combined)
Date - Time May 14, 2018 12:35:52
Operator ID SF
Elapsed Time 00:05:00
Mean Diam. 3.9 nm
Rel. Var. 0.025
Skew 6.077

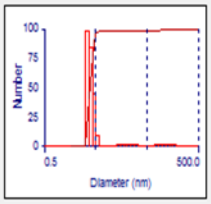

d(nm)	G(d)	C(d)	d(nm)	G(d)	C(d)	d(nm)	G(d)	C(d)
1.8	0	0	10.0	0	100	55.1	0	100
2.1	0	0	11.6	0	100	64.4	0	100
2.5	0	0	13.6	0	100	75.2	0	100
2.9	0	0	15.9	0	100	87.8	0	100
3.4	100	42	18.6	0	100	102.6	0	100
3.9	86	78	21.7	0	100	119.8	0	100
4.6	45	96	25.3	0	100	140.0	0	100
5.4	9	100	29.6	0	100	163.5	0	100
6.3	0	100	34.6	0	100	191.0	0	100
7.3	0	100	40.4	0	100	223.1	0	100
8.5	0	100	47.2	0	100	260.6	0	100

Number

Sample ID NL-G3 run 3 (Combined)
Date - Time May 14, 2018 12:35:52
Operator ID SF
Elapsed Time 00:05:00
Mean Diam. 4.8 nm
Rel. Var. 0.987
Skew 16.058

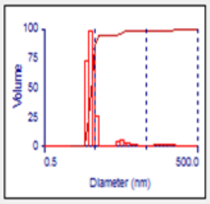

d(nm)	G(d)	C(d)	d(nm)	G(d)	C(d)	d(nm)	G(d)	C(d)
1.8	0	0	10.0	0	96	55.1	0	100
2.1	0	0	11.6	0	96	64.4	0	100
2.5	0	0	13.6	4	97	75.2	0	100
2.9	0	0	15.9	4	98	87.8	0	100
3.4	73	25	18.6	2	99	102.6	0	100
3.9	100	59	21.7	1	100	119.8	0	100
4.6	84	87	25.3	0	100	140.0	0	100
5.4	25	96	29.6	0	100	163.5	0	100
6.3	0	96	34.6	0	100	191.0	0	100
7.3	0	96	40.4	0	100	223.1	0	100
8.5	0	96	47.2	0	100	260.6	0	100

Volume

Supplementary Figure 33. Size distribution of NL-G3 in PBS. **a** Number- and **b** volume-weighted. Data in columns are technical replicates of **a** or **b**.

a

Sample ID	NL-G3 (Combined)
Date - Time	May 14, 2018 12:07:15
Operator ID	SF
Elapsed Time	00:05:00
Mean Diam.	3.4 nm
Rel. Var.	0.204
Skew	17.729

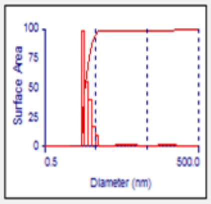

d(nm)	G(d)	C(d)	d(nm)	G(d)	C(d)	d(nm)	G(d)	C(d)
1.7	0	0	9.6	0	99	53.5	0	100
2.0	0	0	11.2	0	99	62.6	0	100
2.4	0	0	13.1	0	99	73.1	0	100
2.8	100	45	15.4	1	100	85.5	0	100
3.2	56	71	17.9	0	100	100.0	0	100
3.8	39	88	21.0	0	100	116.8	0	100
4.4	16	95	24.5	0	100	136.6	0	100
5.1	8	99	28.7	0	100	159.7	0	100
6.0	0	99	33.5	0	100	186.7	0	100
7.0	0	99	39.2	0	100	218.2	0	100
8.2	0	99	45.8	0	100	255.1	0	100

Surface Area

Sample ID	NL-G3 run 2 (Combined)
Date - Time	May 14, 2018 12:27:25
Operator ID	SF
Elapsed Time	00:05:00
Mean Diam.	5.4 nm
Rel. Var.	0.118
Skew	23.437

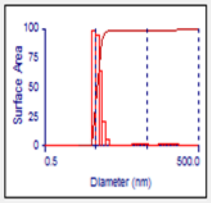

d(nm)	G(d)	C(d)	d(nm)	G(d)	C(d)	d(nm)	G(d)	C(d)
2.5	0	0	12.7	0	100	64.7	0	100
2.9	0	0	14.8	0	100	75.0	0	100
3.4	0	0	17.1	0	100	87.0	0	100
3.9	0	0	19.8	0	100	100.8	0	100
4.5	100	35	23.0	0	100	116.9	0	100
5.3	96	69	26.7	0	100	135.5	0	100
6.1	65	91	30.9	0	100	157.1	0	100
7.1	20	98	35.8	0	100	182.1	0	100
8.2	4	100	41.5	0	100	211.1	0	100
9.5	0	100	48.2	0	100	244.7	0	100
11.0	0	100	55.8	0	100	283.6	0	100

Surface Area

Sample ID	NL-G3 run 3 (Combined)
Date - Time	May 14, 2018 12:35:52
Operator ID	SF
Elapsed Time	00:05:00
Mean Diam.	4.2 nm
Rel. Var.	0.153
Skew	19.771

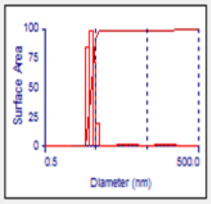

d(nm)	G(d)	C(d)	d(nm)	G(d)	C(d)	d(nm)	G(d)	C(d)
1.8	0	0	10.0	0	99	55.1	0	100
2.1	0	0	11.6	0	99	64.4	0	100
2.5	0	0	13.6	1	99	75.2	0	100
2.9	0	0	15.9	1	100	87.8	0	100
3.4	86	31	18.6	0	100	102.6	0	100
3.9	100	67	21.7	0	100	119.8	0	100
4.6	72	92	25.3	0	100	140.0	0	100
5.4	19	99	29.6	0	100	163.5	0	100
6.3	0	99	34.6	0	100	191.0	0	100
7.3	0	99	40.4	0	100	223.1	0	100
8.5	0	99	47.2	0	100	260.6	0	100

Surface Area

b

Sample ID	NL-G3 (Combined)
Date - Time	May 14, 2018 12:07:15
Operator ID	SF
Elapsed Time	00:05:00
Mean Diam.	97.8 nm
Rel. Var.	0.197
Skew	-0.888

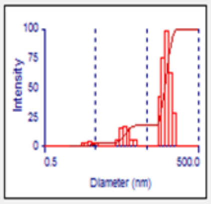

d(nm)	G(d)	C(d)	d(nm)	G(d)	C(d)	d(nm)	G(d)	C(d)
1.7	0	0	9.6	0	3	53.5	0	19
2.0	0	0	11.2	0	3	62.6	0	19
2.4	0	0	13.1	5	4	73.1	0	19
2.8	2	1	15.4	15	8	85.5	42	30
3.2	2	1	17.9	16	12	100.0	76	50
3.8	3	2	21.0	14	16	116.8	100	76
4.4	2	2	24.5	5	17	136.6	63	93
5.1	2	3	28.7	5	19	159.7	28	100
6.0	0	3	33.5	0	19	186.7	0	100
7.0	0	3	39.2	0	19	218.2	0	100
8.2	0	3	45.8	0	19	255.1	0	100

Intensity

Sample ID	NL-G3 run 2 (Combined)
Date - Time	May 14, 2018 12:27:25
Operator ID	SF
Elapsed Time	00:05:00
Mean Diam.	102.9 nm
Rel. Var.	0.241
Skew	-0.571

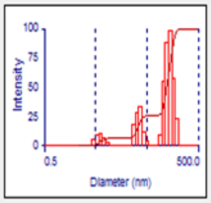

d(nm)	G(d)	C(d)	d(nm)	G(d)	C(d)	d(nm)	G(d)	C(d)
2.5	0	0	12.7	0	6	64.7	0	27
2.9	0	0	14.8	0	6	75.0	0	27
3.4	0	0	17.1	0	6	87.0	8	28
3.9	0	0	19.8	0	6	100.8	56	41
4.5	5	1	23.0	0	6	116.9	89	60
5.3	8	3	26.7	18	10	135.5	100	82
6.1	10	5	30.9	28	16	157.1	58	95
7.1	5	6	35.8	33	24	182.1	23	100
8.2	2	6	41.5	11	26	211.1	0	100
9.5	0	6	48.2	3	27	244.7	0	100
11.0	0	6	55.8	0	27	283.6	0	100

Intensity

Sample ID	NL-G3 run 3 (Combined)
Date - Time	May 14, 2018 12:35:52
Operator ID	SF
Elapsed Time	00:05:00
Mean Diam.	98.3 nm
Rel. Var.	0.189
Skew	-0.899

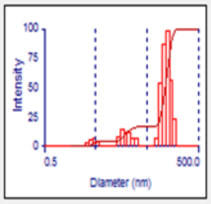

d(nm)	G(d)	C(d)	d(nm)	G(d)	C(d)	d(nm)	G(d)	C(d)
1.8	0	0	10.0	0	4	55.1	0	18
2.1	0	0	11.6	0	4	64.4	0	18
2.5	0	0	13.6	7	6	75.2	6	19
2.9	0	0	15.9	13	9	87.8	54	33
3.4	2	1	18.6	11	12	102.6	87	55
3.9	5	2	21.7	11	14	119.8	100	80
4.6	6	3	25.3	6	16	140.0	57	94
5.4	3	4	29.6	6	18	163.5	23	100
6.3	0	4	34.6	0	18	191.0	0	100
7.3	0	4	40.4	0	18	223.1	0	100
8.5	0	4	47.2	0	18	260.6	0	100

Intensity

Supplementary Figure 34. Size distribution of NL-G3 in PBS. **a** Surface area- and **b** intensity-weighted. Data in columns are technical replicates of **a** or **b**.

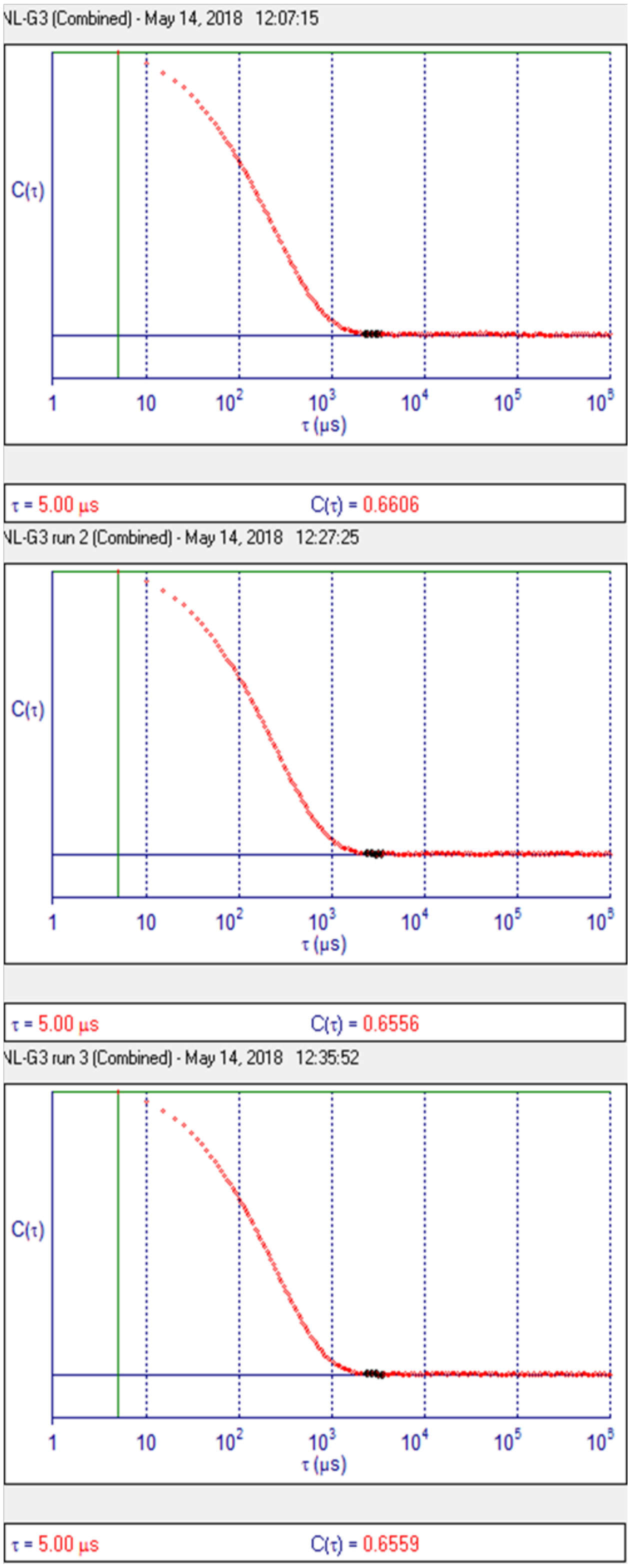

Supplementary Figure 35. Correlation functions for DLS measurements of NL-G3. Data are technical replicates.

a

Sample ID	NL-TT-G3 run 1 (Combined)
Date - Time	May 14, 2018 12:57:36
Operator ID	SF
Elapsed Time	00:05:00
Mean Diam.	9.6 nm
Rel. Var.	0.009
Skew	9.925

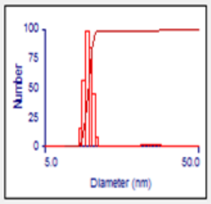

d(nm)	G(d)	C(d)	d(nm)	G(d)	C(d)	d(nm)	G(d)	C(d)
7.0	0	0	12.0	0	100	20.6	0	100
7.4	0	0	12.6	0	100	21.6	0	100
7.7	0	0	13.2	0	100	22.7	0	100
8.1	0	0	13.9	0	100	23.8	0	100
8.5	15	5	14.6	0	100	25.0	0	100
8.9	57	23	15.3	0	100	26.3	0	100
9.4	100	56	16.1	0	100	27.6	0	100
9.9	84	83	16.9	0	100	29.0	0	100
10.4	45	98	17.8	0	100	30.5	0	100
10.9	7	100	18.7	0	100	32.0	0	100
11.4	0	100	19.6	0	100	33.6	0	100

Number

Sample ID	NL-TT-G3 run 2 (Combined)
Date - Time	May 14, 2018 13:43:24
Operator ID	SF
Elapsed Time	00:05:00
Mean Diam.	9.5 nm
Rel. Var.	0.008
Skew	9.397

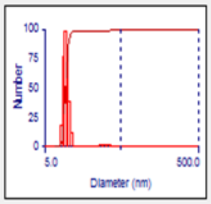

d(nm)	G(d)	C(d)	d(nm)	G(d)	C(d)	d(nm)	G(d)	C(d)
7.0	0	0	13.2	0	100	24.9	0	100
7.4	0	0	14.0	0	100	26.4	0	100
7.8	0	0	14.8	0	100	28.0	0	100
8.3	18	5	15.7	0	100	29.7	0	100
8.8	78	29	16.6	0	100	31.5	0	100
9.3	100	59	17.6	0	100	33.3	0	100
9.9	86	85	18.7	0	100	35.3	0	100
10.5	38	97	19.8	0	100	37.4	0	100
11.1	11	100	21.0	0	100	39.7	0	100
11.7	0	100	22.2	0	100	42.0	0	100
12.4	0	100	23.5	0	100	44.5	0	100

Number

Sample ID	NL-TT-G3 run 3 (Combined)
Date - Time	May 14, 2018 15:59:38
Operator ID	SF
Elapsed Time	00:05:00
Mean Diam.	9.6 nm
Rel. Var.	0.009
Skew	9.769

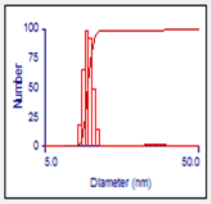

d(nm)	G(d)	C(d)	d(nm)	G(d)	C(d)	d(nm)	G(d)	C(d)
6.8	0	0	12.1	0	100	21.6	0	100
7.2	0	0	12.8	0	100	22.7	0	100
7.6	0	0	13.5	0	100	23.9	0	100
8.0	0	0	14.2	0	100	25.2	0	100
8.4	17	5	15.0	0	100	26.6	0	100
8.9	65	24	15.8	0	100	28.0	0	100
9.4	100	54	16.6	0	100	29.5	0	100
9.9	93	81	17.5	0	100	31.0	0	100
10.4	48	96	18.4	0	100	32.7	0	100
10.9	14	100	19.4	0	100	34.5	0	100
11.5	0	100	20.5	0	100	36.3	0	100

Number

b

Sample ID	NL-TT-G3 run 2 (Combined)
Date - Time	May 14, 2018 13:43:24
Operator ID	SF
Elapsed Time	00:05:00
Mean Diam.	10.0 nm
Rel. Var.	0.082
Skew	6.689

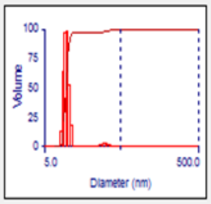

d(nm)	G(d)	C(d)	d(nm)	G(d)	C(d)	d(nm)	G(d)	C(d)
7.0	0	0	13.2	0	98	24.9	0	98
7.4	0	0	14.0	0	98	26.4	1	98
7.8	0	0	14.8	0	98	28.0	1	99
8.3	13	4	15.7	0	98	29.7	2	99
8.8	64	22	16.6	0	98	31.5	2	100
9.3	98	50	17.6	0	98	33.3	1	100
9.9	100	78	18.7	0	98	35.3	0	100
10.5	52	93	19.8	0	98	37.4	0	100
11.1	17	98	21.0	0	98	39.7	0	100
11.7	0	98	22.2	0	98	42.0	0	100
12.4	0	98	23.5	0	98	44.5	0	100

Volume

Sample ID	NL-TT-G3 run 1 (Combined)
Date - Time	May 14, 2018 12:57:36
Operator ID	SF
Elapsed Time	00:05:00
Mean Diam.	10.2 nm
Rel. Var.	0.075
Skew	4.424

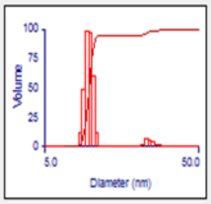

d(nm)	G(d)	C(d)	d(nm)	G(d)	C(d)	d(nm)	G(d)	C(d)
7.0	0	0	12.0	0	96	20.6	0	96
7.4	0	0	12.6	0	96	21.6	1	96
7.7	0	0	13.2	0	96	22.7	5	98
8.1	0	0	13.9	0	96	23.8	4	99
8.5	11	3	14.6	0	96	25.0	3	100
8.9	49	18	15.3	0	96	26.3	0	100
9.4	100	47	16.1	0	96	27.6	0	100
9.9	98	75	16.9	0	96	29.0	0	100
10.4	61	93	17.8	0	96	30.5	0	100
10.9	11	96	18.7	0	96	32.0	0	100
11.4	0	96	19.6	0	96	33.6	0	100

Volume

Sample ID	NL-TT-G3 run 3 (Combined)
Date - Time	May 14, 2018 15:59:38
Operator ID	SF
Elapsed Time	00:05:00
Mean Diam.	10.2 nm
Rel. Var.	0.085
Skew	4.735

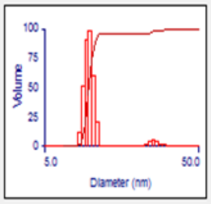

d(nm)	G(d)	C(d)	d(nm)	G(d)	C(d)	d(nm)	G(d)	C(d)
6.8	0	0	12.1	0	96	21.6	0	96
7.2	0	0	12.8	0	96	22.7	1	97
7.6	0	0	13.5	0	96	23.9	3	98
8.0	0	0	14.2	0	96	25.2	4	99
8.4	11	3	15.0	0	96	26.6	3	100
8.9	52	18	15.8	0	96	28.0	1	100
9.4	92	45	16.6	0	96	29.5	0	100
9.9	100	73	17.5	0	96	31.0	0	100
10.4	61	91	18.4	0	96	32.7	0	100
10.9	20	96	19.4	0	96	34.5	0	100
11.5	0	96	20.5	0	96	36.3	0	100

Volume

Supplementary Figure 36. Size distribution of NL-TT-G3 in PBS. **a** Number- and **b** volume-weighted. Data in columns are technical replicates of **a** or **b**.

a

Sample ID	NL-TT-G3 run 1 (Combined)
Date - Time	May 14, 2018 12:57:36
Operator ID	SF
Elapsed Time	00:05:00
Mean Diam.	9.9 nm
Rel. Var.	0.036
Skew	6.588

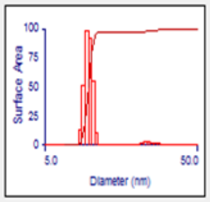

d(nm)	G(d)	C(d)	d(nm)	G(d)	C(d)	d(nm)	G(d)	C(d)
7.0	0	0	12.0	0	98	20.6	0	98
7.4	0	0	12.6	0	98	21.6	1	98
7.7	0	0	13.2	0	98	22.7	2	99
8.1	0	0	13.9	0	98	23.8	2	100
8.5	13	4	14.6	0	98	25.0	1	100
8.9	52	20	15.3	0	98	26.3	0	100
9.4	100	50	16.1	0	98	27.6	0	100
9.9	93	79	16.9	0	98	29.0	0	100
10.4	55	95	17.8	0	98	30.5	0	100
10.9	9	98	18.7	0	98	32.0	0	100
11.4	0	98	19.6	0	98	33.6	0	100

Surface Area

Sample ID	NL-TT-G3 run 2 (Combined)
Date - Time	May 14, 2018 13:43:24
Operator ID	SF
Elapsed Time	00:05:00
Mean Diam.	9.7 nm
Rel. Var.	0.031
Skew	10.218

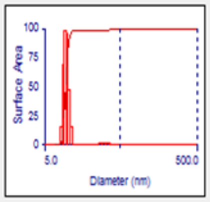

d(nm)	G(d)	C(d)	d(nm)	G(d)	C(d)	d(nm)	G(d)	C(d)
7.0	0	0	13.2	0	99	24.9	0	99
7.4	0	0	14.0	0	99	26.4	0	99
7.8	0	0	14.8	0	99	28.0	0	100
8.3	14	4	15.7	0	99	29.7	1	100
8.8	69	24	16.6	0	99	31.5	0	100
9.3	100	53	17.6	0	99	33.3	0	100
9.9	96	81	18.7	0	99	35.3	0	100
10.5	48	95	19.8	0	99	37.4	0	100
11.1	15	99	21.0	0	99	39.7	0	100
11.7	0	99	22.2	0	99	42.0	0	100
12.4	0	99	23.5	0	99	44.5	0	100

Surface Area

Sample ID	NL-TT-G3 run 3 (Combined)
Date - Time	May 14, 2018 15:59:38
Operator ID	SF
Elapsed Time	00:05:00
Mean Diam.	9.9 nm
Rel. Var.	0.039
Skew	7.144

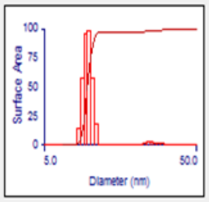

d(nm)	G(d)	C(d)	d(nm)	G(d)	C(d)	d(nm)	G(d)	C(d)
6.8	0	0	12.1	0	99	21.6	0	99
7.2	0	0	12.8	0	99	22.7	0	99
7.6	0	0	13.5	0	99	23.9	1	99
8.0	0	0	14.2	0	99	25.2	2	100
8.4	13	4	15.0	0	99	26.6	1	100
8.9	57	20	15.8	0	99	28.0	0	100
9.4	97	48	16.6	0	99	29.5	0	100
9.9	100	77	17.5	0	99	31.0	0	100
10.4	58	93	18.4	0	99	32.7	0	100
10.9	18	99	19.4	0	99	34.5	0	100
11.5	0	99	20.5	0	99	36.3	0	100

Surface Area

b

Sample ID	NL-TT-G3 run 1 (Combined)
Date - Time	May 14, 2018 12:57:36
Operator ID	SF
Elapsed Time	00:05:00
Mean Diam.	15.0 nm
Rel. Var.	0.206
Skew	0.525

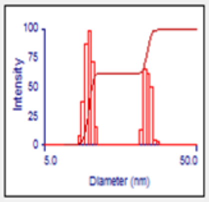

d(nm)	G(d)	C(d)	d(nm)	G(d)	C(d)	d(nm)	G(d)	C(d)
7.0	0	0	12.0	0	62	20.6	0	62
7.4	0	0	12.6	0	62	21.6	12	65
7.7	0	0	13.2	0	62	22.7	65	77
8.1	0	0	13.9	0	62	23.8	62	89
8.5	7	1	14.6	0	62	25.0	50	99
8.9	37	9	15.3	0	62	26.3	3	100
9.4	88	26	16.1	0	62	27.6	2	100
9.9	100	45	16.9	0	62	29.0	0	100
10.4	72	59	17.8	0	62	30.5	0	100
10.9	14	62	18.7	0	62	32.0	0	100
11.4	0	62	19.6	0	62	33.6	0	100

Intensity

Sample ID	NL-TT-G3 run 2 (Combined)
Date - Time	May 14, 2018 13:43:24
Operator ID	SF
Elapsed Time	00:05:00
Mean Diam.	17.3 nm
Rel. Var.	0.342
Skew	0.613

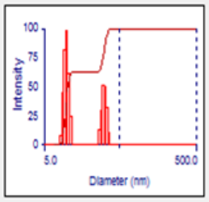

d(nm)	G(d)	C(d)	d(nm)	G(d)	C(d)	d(nm)	G(d)	C(d)
7.0	0	0	13.2	0	64	24.9	0	64
7.4	0	0	14.0	0	64	26.4	12	66
7.8	0	0	14.8	0	64	28.0	29	72
8.3	7	1	15.7	0	64	29.7	51	82
8.8	45	10	16.6	0	64	31.5	50	92
9.3	82	27	17.6	0	64	33.3	32	98
9.9	100	46	18.7	0	64	35.3	10	100
10.5	62	59	19.8	0	64	37.4	0	100
11.1	25	64	21.0	0	64	39.7	0	100
11.7	0	64	22.2	0	64	42.0	0	100
12.4	0	64	23.5	0	64	44.5	0	100

Intensity

Sample ID	NL-TT-G3 run 3 (Combined)
Date - Time	May 14, 2018 15:59:38
Operator ID	SF
Elapsed Time	00:05:00
Mean Diam.	16.1 nm
Rel. Var.	0.236
Skew	0.460

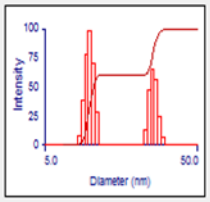

d(nm)	G(d)	C(d)	d(nm)	G(d)	C(d)	d(nm)	G(d)	C(d)
6.8	0	0	12.1	0	60	21.6	0	60
7.2	0	0	12.8	0	60	22.7	13	63
7.6	0	0	13.5	0	60	23.9	47	72
8.0	0	0	14.2	0	60	25.2	65	84
8.4	7	1	15.0	0	60	26.6	57	94
8.9	38	8	15.8	0	60	28.0	24	99
9.4	79	23	16.6	0	60	29.5	6	100
9.9	100	42	17.5	0	60	31.0	0	100
10.4	71	55	18.4	0	60	32.7	0	100
10.9	28	60	19.4	0	60	34.5	0	100
11.5	0	60	20.5	0	60	36.3	0	100

Intensity

Supplementary Figure 37. Size distribution of NL-TT-G3 in PBS. **a** Surface area- and **b** intensity-weighted. Data in columns are technical replicates of **a** or **b**.

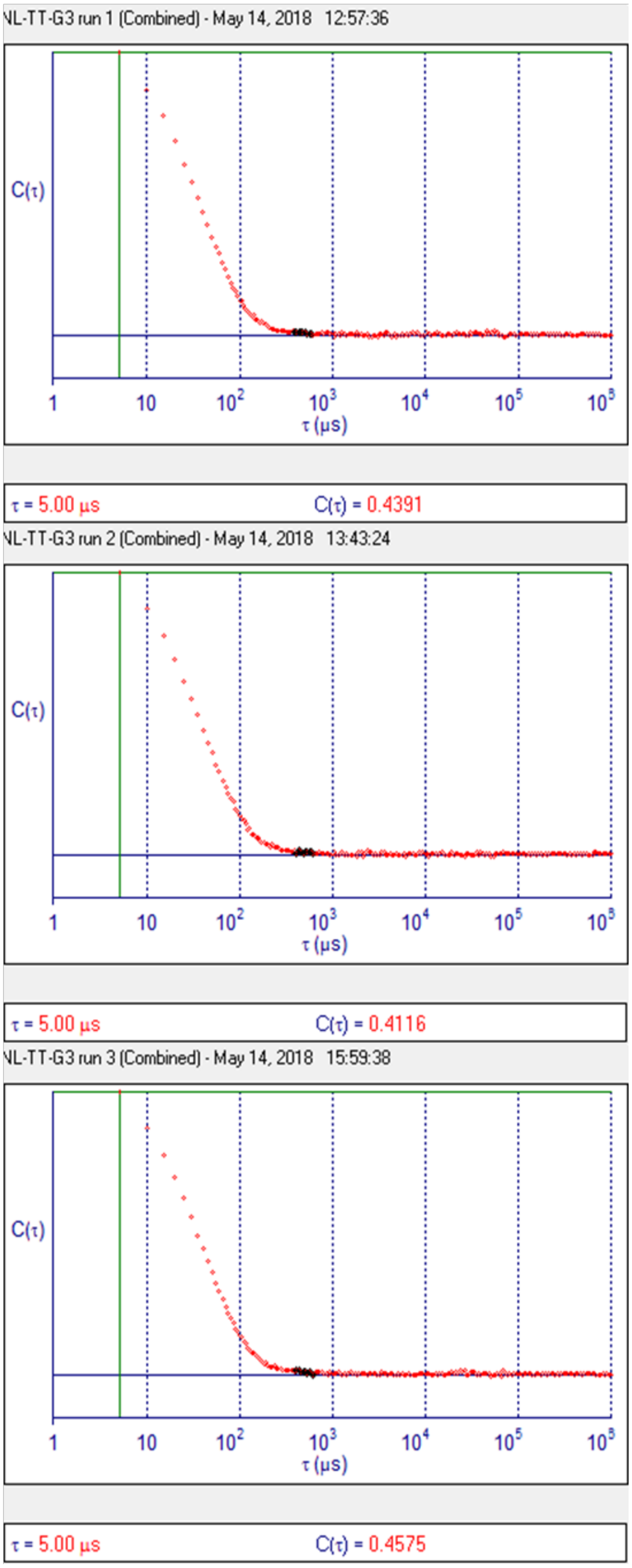

Supplementary Figure 38. Correlation functions for DLS measurements of NL-TT-G3. Data are technical replicates.

a

Sample ID	GFP-G3 run 1 (Combined)
Date - Time	May 14, 2018 13:14:57
Operator ID	SF
Elapsed Time	00:05:00
Mean Diam.	5.8 nm
Rel. Var.	0.030
Skew	2.906

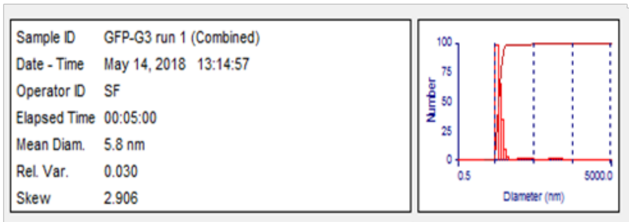

d(nm)	G(d)	C(d)	d(nm)	G(d)	C(d)	d(nm)	G(d)	C(d)
2.7	0	0	15.6	0	100	92.2	0	100
3.1	0	0	18.4	0	100	108.3	0	100
3.7	0	0	21.6	0	100	127.2	0	100
4.3	0	0	25.4	0	100	149.5	0	100
5.1	100	47	29.8	0	100	175.6	0	100
5.9	65	78	35.0	0	100	206.4	0	100
7.0	35	95	41.2	0	100	242.5	0	100
8.2	9	99	48.4	0	100	284.9	0	100
9.6	2	100	56.8	0	100	334.7	0	100
11.3	0	100	66.8	0	100	393.3	0	100
13.3	0	100	78.4	0	100	462.1	0	100

Number

Sample ID	GFP-G3 run 2 (Combined)
Date - Time	May 14, 2018 13:23:55
Operator ID	SF
Elapsed Time	00:05:00
Mean Diam.	5.2 nm
Rel. Var.	0.038
Skew	2.573

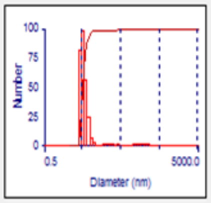

d(nm)	G(d)	C(d)	d(nm)	G(d)	C(d)	d(nm)	G(d)	C(d)
2.6	0	0	15.3	0	100	90.0	0	100
3.0	0	0	17.9	0	100	105.7	0	100
3.6	0	0	21.1	0	100	124.2	0	100
4.2	83	31	24.8	0	100	145.9	0	100
4.9	100	68	29.1	0	100	171.4	0	100
5.8	56	88	34.2	0	100	201.4	0	100
6.8	24	97	40.2	0	100	236.7	0	100
8.0	6	99	47.2	0	100	278.1	0	100
9.4	2	100	55.5	0	100	326.7	0	100
11.1	0	100	65.2	0	100	383.9	0	100
13.0	0	100	76.6	0	100	451.0	0	100

Number

Sample ID	GFP-G3 run 3 (Combined)
Date - Time	May 14, 2018 13:32:27
Operator ID	SF
Elapsed Time	00:05:00
Mean Diam.	4.0 nm
Rel. Var.	0.038
Skew	4.169

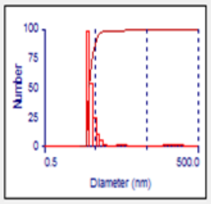

d(nm)	G(d)	C(d)	d(nm)	G(d)	C(d)	d(nm)	G(d)	C(d)
2.6	0	0	13.3	0	100	68.1	0	100
3.0	0	0	15.4	0	100	79.0	0	100
3.5	100	52	17.9	0	100	91.6	0	100
4.0	55	80	20.7	0	100	106.3	0	100
4.7	24	92	24.1	0	100	123.3	0	100
5.4	10	97	27.9	0	100	143.1	0	100
6.3	4	99	32.4	0	100	166.0	0	100
7.3	1	100	37.6	0	100	192.5	0	100
8.5	0	100	43.6	0	100	223.4	0	100
9.9	0	100	50.6	0	100	259.2	0	100
11.4	0	100	58.7	0	100	300.7	0	100

Number

b

Sample ID	GFP-G3 run 1 (Combined)
Date - Time	May 14, 2018 13:14:57
Operator ID	SF
Elapsed Time	00:05:00
Mean Diam.	6.8 nm
Rel. Var.	0.416
Skew	25.560

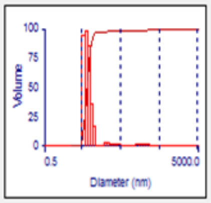

d(nm)	G(d)	C(d)	d(nm)	G(d)	C(d)	d(nm)	G(d)	C(d)
2.7	0	0	15.6	0	98	92.2	0	100
3.1	0	0	18.4	2	99	108.3	0	100
3.7	0	0	21.6	2	99	127.2	0	100
4.3	0	0	25.4	1	100	149.5	0	100
5.1	95	28	29.8	1	100	175.6	0	100
5.9	100	57	35.0	0	100	206.4	0	100
7.0	87	83	41.2	0	100	242.5	0	100
8.2	36	93	48.4	0	100	284.9	0	100
9.6	16	98	56.8	0	100	334.7	0	100
11.3	0	98	66.8	0	100	393.3	0	100
13.3	0	98	78.4	0	100	462.1	0	100

Volume

Sample ID	GFP-G3 run 2 (Combined)
Date - Time	May 14, 2018 13:23:55
Operator ID	SF
Elapsed Time	00:05:00
Mean Diam.	6.2 nm
Rel. Var.	0.423
Skew	25.258

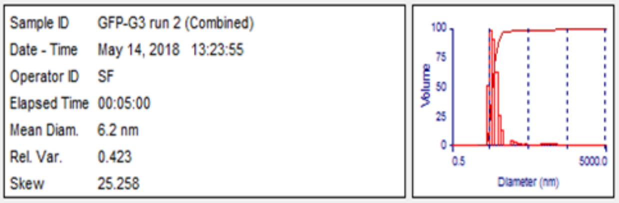

d(nm)	G(d)	C(d)	d(nm)	G(d)	C(d)	d(nm)	G(d)	C(d)
2.6	0	0	15.3	0	98	90.0	0	100
3.0	0	0	17.9	3	99	105.7	0	100
3.6	0	0	21.1	3	99	124.2	0	100
4.2	51	15	24.8	1	100	145.9	0	100
4.9	100	43	29.1	0	100	171.4	0	100
5.8	91	69	34.2	0	100	201.4	0	100
6.8	63	87	40.2	0	100	236.7	0	100
8.0	25	94	47.2	0	100	278.1	0	100
9.4	12	98	55.5	0	100	326.7	0	100
11.1	0	98	65.2	0	100	383.9	0	100
13.0	0	98	76.6	0	100	451.0	0	100

Volume

Sample ID	GFP-G3 run 3 (Combined)
Date - Time	May 14, 2018 13:32:27
Operator ID	SF
Elapsed Time	00:05:00
Mean Diam.	5.0 nm
Rel. Var.	0.525
Skew	23.963

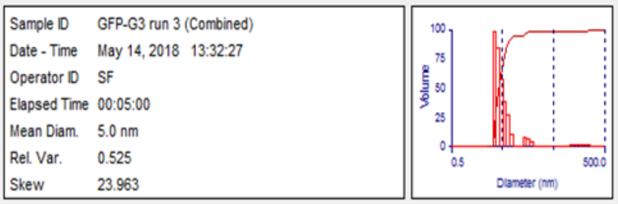

d(nm)	G(d)	C(d)	d(nm)	G(d)	C(d)	d(nm)	G(d)	C(d)
2.6	0	0	13.3	7	97	68.1	0	100
3.0	0	0	15.4	5	99	79.0	0	100
3.5	100	30	17.9	3	100	91.6	0	100
4.0	85	56	20.7	0	100	106.3	0	100
4.7	57	73	24.1	0	100	123.3	0	100
5.4	39	84	27.9	0	100	143.1	0	100
6.3	26	92	32.4	0	100	166.0	0	100
7.3	10	95	37.6	0	100	192.5	0	100
8.5	0	95	43.6	0	100	223.4	0	100
9.9	0	95	50.6	0	100	259.2	0	100
11.4	0	95	58.7	0	100	300.7	0	100

Volume

Supplementary Figure 39. Size distribution of GFP-G3 in PBS. **a** Number- and **b** volume-weighted. Data in columns are technical replicates of **a** or **b**.

a

Sample ID	GFP-G3 run 1 (Combined)
Date - Time	May 14, 2018 13:14:57
Operator ID	SF
Elapsed Time	00:05:00
Mean Diam.	6.3 nm
Rel. Var.	0.081
Skew	17.804

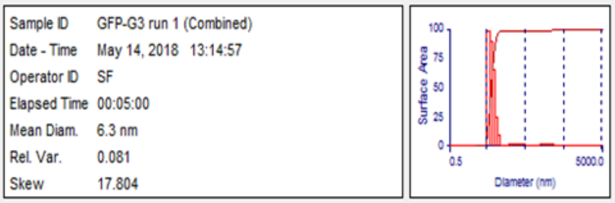

d(nm)	G(d)	C(d)	d(nm)	G(d)	C(d)	d(nm)	G(d)	C(d)
2.7	0	0	15.6	0	99	92.2	0	100
3.1	0	0	18.4	1	100	108.3	0	100
3.7	0	0	21.6	1	100	127.2	0	100
4.3	0	0	25.4	0	100	149.5	0	100
5.1	100	34	29.8	0	100	175.6	0	100
5.9	90	65	35.0	0	100	206.4	0	100
7.0	66	88	41.2	0	100	242.5	0	100
8.2	24	96	48.4	0	100	284.9	0	100
9.6	9	99	56.8	0	100	334.7	0	100
11.3	0	99	66.8	0	100	393.3	0	100
13.3	0	99	78.4	0	100	462.1	0	100

Surface Area

Sample ID	GFP-G3 run 2 (Combined)
Date - Time	May 14, 2018 13:23:55
Operator ID	SF
Elapsed Time	00:05:00
Mean Diam.	5.6 nm
Rel. Var.	0.095
Skew	14.463

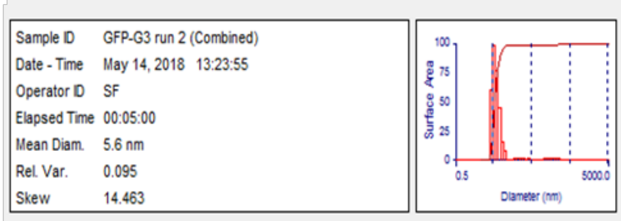

d(nm)	G(d)	C(d)	d(nm)	G(d)	C(d)	d(nm)	G(d)	C(d)
2.6	0	0	15.3	0	99	90.0	0	100
3.0	0	0	17.9	1	100	105.7	0	100
3.6	0	0	21.1	1	100	124.2	0	100
4.2	60	20	24.8	0	100	145.9	0	100
4.9	100	52	29.1	0	100	171.4	0	100
5.8	78	78	34.2	0	100	201.4	0	100
6.8	45	92	40.2	0	100	236.7	0	100
8.0	15	97	47.2	0	100	278.1	0	100
9.4	7	99	55.5	0	100	326.7	0	100
11.1	0	99	65.2	0	100	383.9	0	100
13.0	0	99	76.6	0	100	451.0	0	100

Surface Area

Sample ID	GFP-G3 run 3 (Combined)
Date - Time	May 14, 2018 13:32:27
Operator ID	SF
Elapsed Time	00:05:00
Mean Diam.	4.4 nm
Rel. Var.	0.132
Skew	11.600

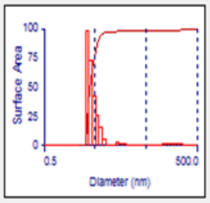

d(nm)	G(d)	C(d)	d(nm)	G(d)	C(d)	d(nm)	G(d)	C(d)
2.6	0	0	13.3	2	99	68.1	0	100
3.0	0	0	15.4	1	100	79.0	0	100
3.5	100	38	17.9	1	100	91.6	0	100
4.0	73	66	20.7	0	100	106.3	0	100
4.7	43	82	24.1	0	100	123.3	0	100
5.4	25	91	27.9	0	100	143.1	0	100
6.3	14	97	32.4	0	100	166.0	0	100
7.3	5	99	37.6	0	100	192.5	0	100
8.5	0	99	43.6	0	100	223.4	0	100
9.9	0	99	50.6	0	100	259.2	0	100
11.4	0	99	58.7	0	100	300.7	0	100

Surface Area

b

Sample ID	GFP-G3 run 1 (Combined)
Date - Time	May 14, 2018 13:14:57
Operator ID	SF
Elapsed Time	00:05:00
Mean Diam.	126.3 nm
Rel. Var.	0.370
Skew	-0.476

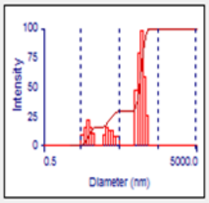

d(nm)	G(d)	C(d)	d(nm)	G(d)	C(d)	d(nm)	G(d)	C(d)
2.7	0	0	15.6	0	16	92.2	0	30
3.1	0	0	18.4	9	18	108.3	0	30
3.7	0	0	21.6	15	21	127.2	47	41
4.3	0	0	25.4	13	24	149.5	79	58
5.1	9	2	29.8	13	27	175.6	100	81
5.9	15	5	35.0	7	28	206.4	59	94
7.0	21	10	41.2	7	30	242.5	26	100
8.2	14	13	48.4	0	30	284.9	0	100
9.6	10	16	56.8	0	30	334.7	0	100
11.3	0	16	66.8	0	30	393.3	0	100
13.3	0	16	78.4	0	30	462.1	0	100

Intensity

Sample ID	GFP-G3 run 2 (Combined)
Date - Time	May 14, 2018 13:23:55
Operator ID	SF
Elapsed Time	00:05:00
Mean Diam.	122.2 nm
Rel. Var.	0.379
Skew	-0.466

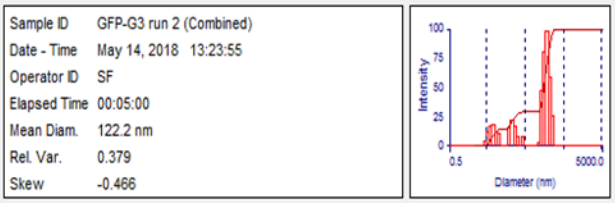

d(nm)	G(d)	C(d)	d(nm)	G(d)	C(d)	d(nm)	G(d)	C(d)
2.6	0	0	15.3	0	15	90.0	0	30
3.0	0	0	17.9	15	19	105.7	2	30
3.6	0	0	21.1	21	23	124.2	48	41
4.2	3	1	24.8	17	27	145.9	81	59
4.9	11	3	29.1	8	29	171.4	100	81
5.8	16	7	34.2	0	29	201.4	59	94
6.8	18	11	40.2	7	30	236.7	25	100
8.0	11	13	47.2	0	30	278.1	0	100
9.4	9	15	55.5	0	30	326.7	0	100
11.1	0	15	65.2	0	30	383.9	0	100
13.0	0	15	76.6	0	30	451.0	0	100

Intensity

Sample ID	GFP-G3 run 3 (Combined)
Date - Time	May 14, 2018 13:32:27
Operator ID	SF
Elapsed Time	00:05:00
Mean Diam.	120.5 nm
Rel. Var.	0.327
Skew	-0.654

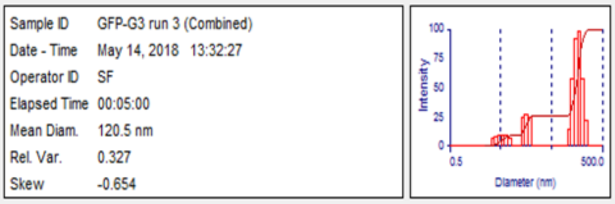

d(nm)	G(d)	C(d)	d(nm)	G(d)	C(d)	d(nm)	G(d)	C(d)
2.6	0	0	13.3	24	15	68.1	0	26
3.0	0	0	15.4	27	21	79.0	0	26
3.5	6	1	17.9	24	26	91.6	0	26
4.0	8	3	20.7	0	26	106.3	14	29
4.7	8	5	24.1	0	26	123.3	58	41
5.4	9	7	27.9	0	26	143.1	93	61
6.3	9	9	32.4	0	26	166.0	100	83
7.3	5	10	37.6	0	26	192.5	58	95
8.5	0	10	43.6	0	26	223.4	22	100
9.9	0	10	50.6	0	26	259.2	0	100
11.4	0	10	58.7	0	26	300.7	0	100

Intensity

Supplementary Figure 40. Size distribution of GFP-G3 in PBS. **a** Surface area- and **b** intensity-weighted. Data in columns are technical replicates of **a** or **b**.

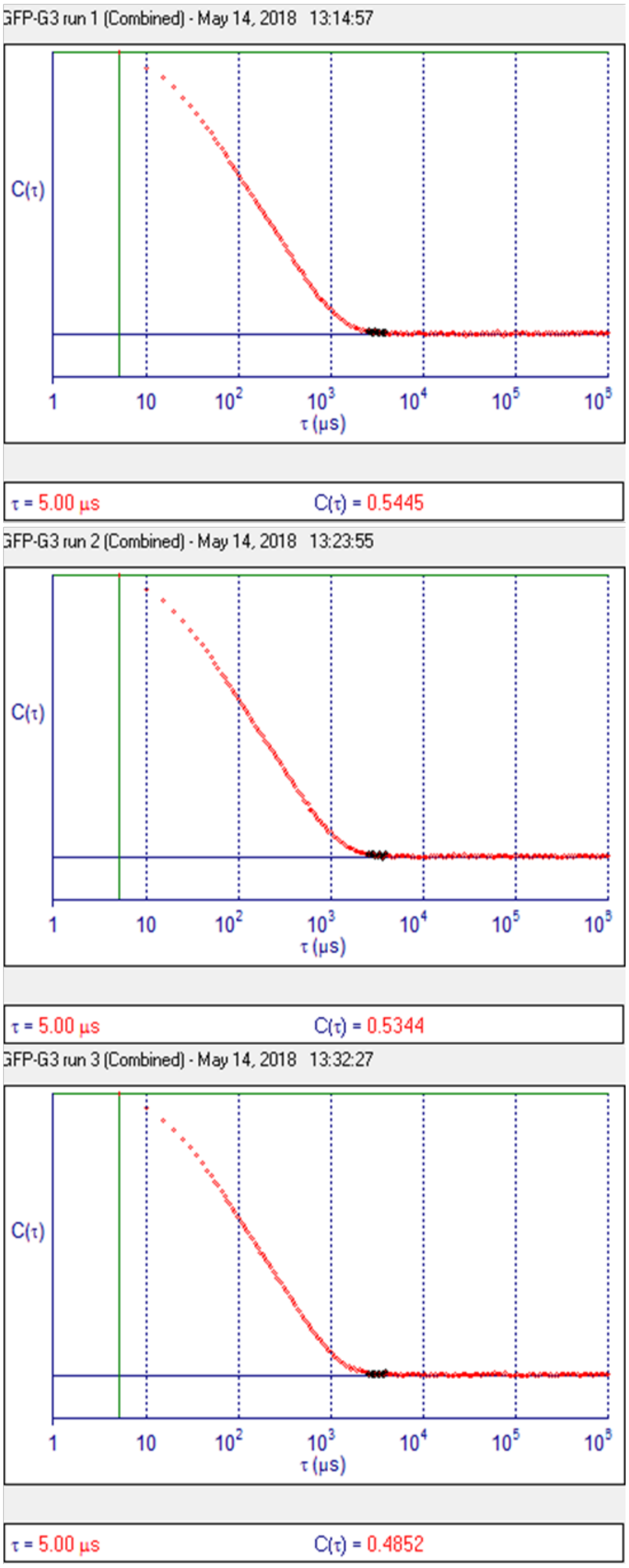

Supplementary Figure 41. Correlation functions for DLS measurements of GFP-G3. Data are technical replicates.

a

Sample ID	GFP-TT-G3 run 1 (Combined)
Date - Time	May 14, 2018 14:28:00
Operator ID	SF
Elapsed Time	00:05:00
Mean Diam.	12.6 nm
Rel. Var.	0.027
Skew	0.899

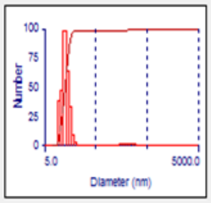

d(nm)	G(d)	C(d)	d(nm)	G(d)	C(d)	d(nm)	G(d)	C(d)
7.4	0	0	29.2	0	100	114.3	0	100
8.4	0	0	33.0	0	100	129.4	0	100
9.5	39	13	37.4	0	100	146.5	0	100
10.8	47	29	42.3	0	100	165.9	0	100
12.2	100	63	47.9	0	100	187.8	0	100
13.9	65	85	54.3	0	100	212.6	0	100
15.7	33	97	61.4	0	100	240.7	0	100
17.8	8	99	69.6	0	100	272.6	0	100
20.1	2	100	78.8	0	100	308.6	0	100
22.8	0	100	89.2	0	100	349.4	0	100
25.8	0	100	101.0	0	100	395.6	0	100

Number

Sample ID	GFP-TT-G3 run 2 (Combined)
Date - Time	May 14, 2018 14:52:57
Operator ID	SF
Elapsed Time	00:05:00
Mean Diam.	12.8 nm
Rel. Var.	0.016
Skew	1.718

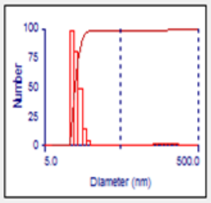

d(nm)	G(d)	C(d)	d(nm)	G(d)	C(d)	d(nm)	G(d)	C(d)
6.9	0	0	26.9	0	100	105.3	0	100
7.8	0	0	30.4	0	100	119.2	0	100
8.8	0	0	34.5	0	100	135.0	0	100
10.0	0	0	39.0	0	100	152.9	0	100
11.3	100	40	44.2	0	100	173.1	0	100
12.8	81	73	50.0	0	100	195.9	0	100
14.5	49	93	56.6	0	100	221.8	0	100
16.4	14	99	64.1	0	100	251.1	0	100
18.5	3	100	72.6	0	100	284.3	0	100
21.0	0	100	82.2	0	100	321.9	0	100
23.7	0	100	93.0	0	100	364.5	0	100

Number

Sample ID	GFP-TT-G3 run 3 (Combined)
Date - Time	May 14, 2018 14:44:50
Operator ID	SF
Elapsed Time	00:05:00
Mean Diam.	12.3 nm
Rel. Var.	0.019
Skew	1.348

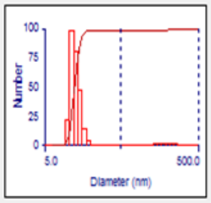

d(nm)	G(d)	C(d)	d(nm)	G(d)	C(d)	d(nm)	G(d)	C(d)
6.8	0	0	26.5	0	100	104.0	0	100
7.7	0	0	30.0	0	100	117.7	0	100
8.7	0	0	34.0	0	100	133.3	0	100
9.8	21	8	38.5	0	100	150.9	0	100
11.1	100	46	43.6	0	100	170.8	0	100
12.6	81	76	49.4	0	100	193.4	0	100
14.3	47	94	55.9	0	100	219.0	0	100
16.1	13	99	63.3	0	100	247.9	0	100
18.3	3	100	71.6	0	100	280.7	0	100
20.7	0	100	81.1	0	100	317.8	0	100
23.4	0	100	91.8	0	100	359.8	0	100

Number

b

Sample ID	GFP-TT-G3 run 1 (Combined)
Date - Time	May 14, 2018 14:28:00
Operator ID	SF
Elapsed Time	00:05:00
Mean Diam.	13.9 nm
Rel. Var.	0.266
Skew	22.986

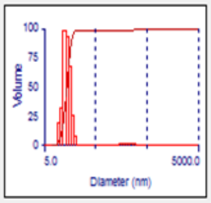

d(nm)	G(d)	C(d)	d(nm)	G(d)	C(d)	d(nm)	G(d)	C(d)
7.4	0	0	29.2	0	100	114.3	0	100
8.4	0	0	33.0	0	100	129.4	0	100
9.5	18	5	37.4	0	100	146.5	0	100
10.8	32	15	42.3	0	100	165.9	0	100
12.2	100	43	47.9	0	100	187.8	0	100
13.9	94	71	54.3	0	100	212.6	0	100
15.7	69	90	61.4	0	100	240.7	0	100
17.8	25	98	69.6	0	100	272.6	0	100
20.1	7	100	78.8	0	100	308.6	0	100
22.8	0	100	89.2	0	100	349.4	0	100
25.8	0	100	101.0	0	100	395.6	0	100

Volume

Sample ID	GFP-TT-G3 run 2 (Combined)
Date - Time	May 14, 2018 14:52:57
Operator ID	SF
Elapsed Time	00:05:00
Mean Diam.	13.7 nm
Rel. Var.	0.237
Skew	22.916

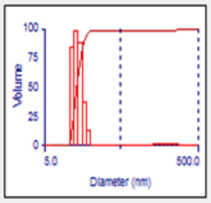

d(nm)	G(d)	C(d)	d(nm)	G(d)	C(d)	d(nm)	G(d)	C(d)
6.9	0	0	26.9	0	100	105.3	0	100
7.8	0	0	30.4	0	100	119.2	0	100
8.8	0	0	34.5	0	100	135.0	0	100
10.0	0	0	39.0	0	100	152.9	0	100
11.3	86	26	44.2	0	100	173.1	0	100
12.8	100	57	50.0	0	100	195.9	0	100
14.5	89	85	56.6	0	100	221.8	0	100
16.4	37	96	64.1	0	100	251.1	0	100
18.5	12	100	72.6	0	100	284.3	0	100
21.0	0	100	82.2	0	100	321.9	0	100
23.7	0	100	93.0	0	100	364.5	0	100

Volume

Sample ID	GFP-TT-G3 run 3 (Combined)
Date - Time	May 14, 2018 14:44:50
Operator ID	SF
Elapsed Time	00:05:00
Mean Diam.	13.3 nm
Rel. Var.	0.229
Skew	23.784

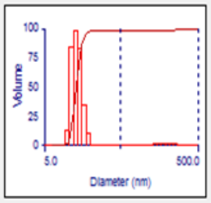

d(nm)	G(d)	C(d)	d(nm)	G(d)	C(d)	d(nm)	G(d)	C(d)
6.8	0	0	26.5	0	100	104.0	0	100
7.7	0	0	30.0	0	100	117.7	0	100
8.7	0	0	34.0	0	100	133.3	0	100
9.8	12	4	38.5	0	100	150.9	0	100
11.1	85	30	43.6	0	100	170.8	0	100
12.6	100	61	49.4	0	100	193.4	0	100
14.3	83	86	55.9	0	100	219.0	0	100
16.1	34	97	63.3	0	100	247.9	0	100
18.3	10	100	71.6	0	100	280.7	0	100
20.7	0	100	81.1	0	100	317.8	0	100
23.4	0	100	91.8	0	100	359.8	0	100

Volume

Supplementary Figure 42. Size distribution of GFP-TT-G3 in PBS. **a** Number- and **b** volume-weighted. Data in columns are technical replicates of **a** or **b**.

a

Sample ID	GFP-TT-G3 run 1 (Combined)
Date - Time	May 14, 2018 14:28:00
Operator ID	SF
Elapsed Time	00:05:00
Mean Diam.	13.3 nm
Rel. Var.	0.046
Skew	25.216

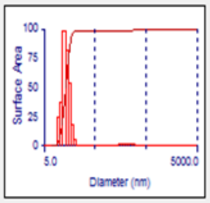

d(nm)	G(d)	C(d)	d(nm)	G(d)	C(d)	d(nm)	G(d)	C(d)
7.4	0	0	29.2	0	100	114.3	0	100
8.4	0	0	33.0	0	100	129.4	0	100
9.5	24	7	37.4	0	100	146.5	0	100
10.8	37	19	42.3	0	100	165.9	0	100
12.2	100	50	47.9	0	100	187.8	0	100
13.9	83	76	54.3	0	100	212.6	0	100
15.7	54	93	61.4	0	100	240.7	0	100
17.8	17	99	69.6	0	100	272.6	0	100
20.1	5	100	78.8	0	100	308.6	0	100
22.8	0	100	89.2	0	100	349.4	0	100
25.8	0	100	101.0	0	100	395.6	0	100

Surface Area

Sample ID	GFP-TT-G3 run 2 (Combined)
Date - Time	May 14, 2018 14:52:57
Operator ID	SF
Elapsed Time	00:05:00
Mean Diam.	13.2 nm
Rel. Var.	0.036
Skew	31.489

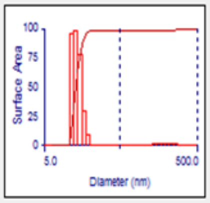

d(nm)	G(d)	C(d)	d(nm)	G(d)	C(d)	d(nm)	G(d)	C(d)
6.9	0	0	26.9	0	100	105.3	0	100
7.8	0	0	30.4	0	100	119.2	0	100
8.8	0	0	34.5	0	100	135.0	0	100
10.0	0	0	39.0	0	100	152.9	0	100
11.3	97	31	44.2	0	100	173.1	0	100
12.8	100	63	50.0	0	100	195.9	0	100
14.5	78	88	56.6	0	100	221.8	0	100
16.4	29	97	64.1	0	100	251.1	0	100
18.5	8	100	72.6	0	100	284.3	0	100
21.0	0	100	82.2	0	100	321.9	0	100
23.7	0	100	93.0	0	100	364.5	0	100

Surface Area

Sample ID	GFP-TT-G3 run 3 (Combined)
Date - Time	May 14, 2018 14:44:50
Operator ID	SF
Elapsed Time	00:05:00
Mean Diam.	12.8 nm
Rel. Var.	0.037
Skew	29.635

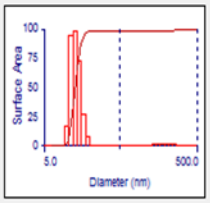

d(nm)	G(d)	C(d)	d(nm)	G(d)	C(d)	d(nm)	G(d)	C(d)
6.8	0	0	26.5	0	100	104.0	0	100
7.7	0	0	30.0	0	100	117.7	0	100
8.7	0	0	34.0	0	100	133.3	0	100
9.8	16	5	38.5	0	100	150.9	0	100
11.1	96	35	43.6	0	100	170.8	0	100
12.6	100	66	49.4	0	100	193.4	0	100
14.3	74	89	55.9	0	100	219.0	0	100
16.1	27	98	63.3	0	100	247.9	0	100
18.3	7	100	71.6	0	100	280.7	0	100
20.7	0	100	81.1	0	100	317.8	0	100
23.4	0	100	91.8	0	100	359.8	0	100

Surface Area

b

Sample ID	GFP-TT-G3 run 1 (Combined)
Date - Time	May 14, 2018 14:28:00
Operator ID	SF
Elapsed Time	00:05:00
Mean Diam.	131.5 nm
Rel. Var.	0.454
Skew	-0.404

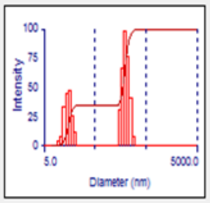

d(nm)	G(d)	C(d)	d(nm)	G(d)	C(d)	d(nm)	G(d)	C(d)
7.4	0	0	29.2	0	35	114.3	0	35
8.4	0	0	33.0	0	35	129.4	0	35
9.5	3	1	37.4	0	35	146.5	31	41
10.8	7	2	42.3	0	35	165.9	67	55
12.2	33	9	47.9	0	35	187.8	100	75
13.9	45	18	54.3	0	35	212.6	77	90
15.7	48	28	61.4	0	35	240.7	41	99
17.8	25	33	69.6	0	35	272.6	7	100
20.1	11	35	78.8	0	35	308.6	0	100
22.8	0	35	89.2	0	35	349.4	0	100
25.8	0	35	101.0	0	35	395.6	0	100

Intensity

Sample ID	GFP-TT-G3 run 2 (Combined)
Date - Time	May 14, 2018 14:52:57
Operator ID	SF
Elapsed Time	00:05:00
Mean Diam.	120.9 nm
Rel. Var.	0.452
Skew	-0.395

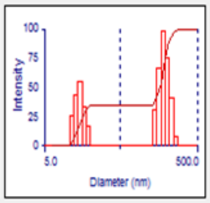

d(nm)	G(d)	C(d)	d(nm)	G(d)	C(d)	d(nm)	G(d)	C(d)
6.9	0	0	26.9	0	35	105.3	0	35
7.8	0	0	30.4	0	35	119.2	0	35
8.8	0	0	34.5	0	35	135.0	31	41
10.0	0	0	39.0	0	35	152.9	67	55
11.3	25	5	44.2	0	35	173.1	100	75
12.8	43	14	50.0	0	35	195.9	76	90
14.5	55	25	56.6	0	35	221.8	41	99
16.4	33	32	64.1	0	35	251.1	7	100
18.5	16	35	72.6	0	35	284.3	0	100
21.0	0	35	82.2	0	35	321.9	0	100
23.7	0	35	93.0	0	35	364.5	0	100

Intensity

Sample ID	GFP-TT-G3 run 3 (Combined)
Date - Time	May 14, 2018 14:44:50
Operator ID	SF
Elapsed Time	00:05:00
Mean Diam.	119.6 nm
Rel. Var.	0.466
Skew	-0.371

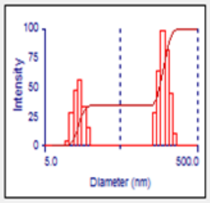

d(nm)	G(d)	C(d)	d(nm)	G(d)	C(d)	d(nm)	G(d)	C(d)
6.8	0	0	26.5	0	36	104.0	0	36
7.7	0	0	30.0	0	36	117.7	0	36
8.7	0	0	34.0	0	36	133.3	27	41
9.8	3	1	38.5	0	36	150.9	65	54
11.1	27	6	43.6	0	36	170.8	100	73
12.6	47	15	49.4	0	36	193.4	82	89
14.3	57	26	55.9	0	36	219.0	45	98
16.1	34	33	63.3	0	36	247.9	9	100
18.3	14	36	71.6	0	36	280.7	0	100
20.7	0	36	81.1	0	36	317.8	0	100
23.4	0	36	91.8	0	36	359.8	0	100

Intensity

Supplementary Figure 43. Size distribution of GFP-TT-G3 in PBS. **a** Surface area- and **b** intensity-weighted. Data in columns are technical replicates of **a** or **b**.

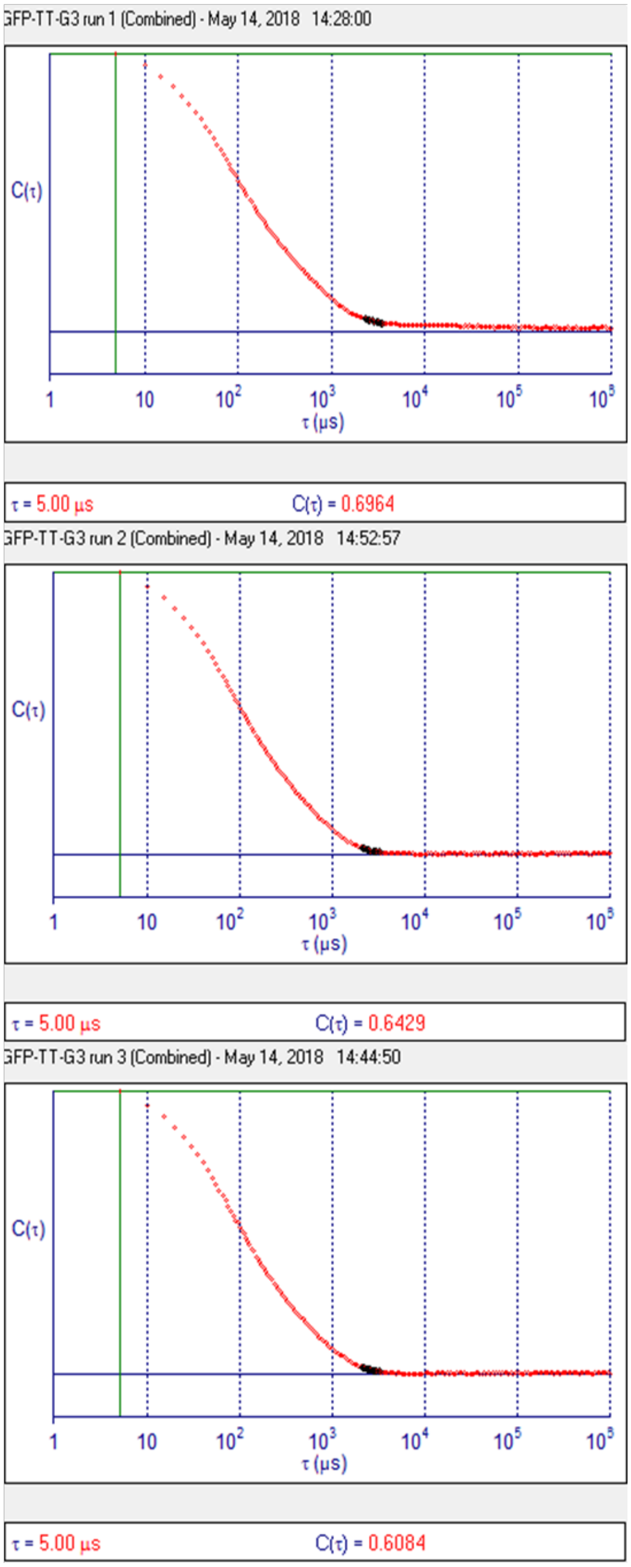

Supplementary Figure 44. Correlation functions for DLS measurements of GFP-TT-G3. Data are technical replicates.

Sample ID	ChaABC-Gal3 (Combined)
Date - Time	Jun 1, 2018 11:08:17
Operator ID	DS
Elapsed Time	00:05:00
Mean Diam.	6.3 nm
Rel. Var.	0.034
Skew	3.140

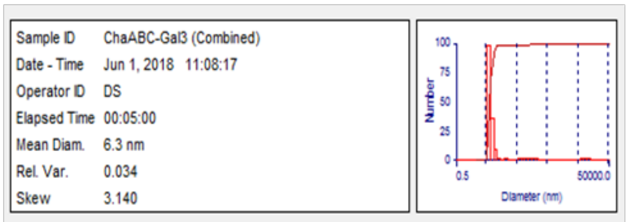

d(nm)	G(d)	C(d)	d(nm)	G(d)	C(d)	d(nm)	G(d)	C(d)
2.6	0	0	44.5	0	100	758.8	0	100
3.4	0	0	57.6	0	100	982.0	0	100
4.4	0	0	74.5	0	100	1270.9	0	100
5.7	100	69	96.4	0	100	1644.7	0	100
7.3	36	93	124.8	0	100	2128.5	0	100
9.5	9	100	161.5	0	100	2754.6	0	100
12.3	1	100	209.0	0	100	3564.9	0	100
15.9	0	100	270.5	0	100	4613.6	0	100
20.5	0	100	350.1	0	100	5970.7	0	100
26.6	0	100	453.0	0	100	7727.0	0	100
34.4	0	100	586.3	0	100	10000.0	0	100

Number

Sample ID	ChaABC-Gal3 (Combined)
Date - Time	Jun 1, 2018 11:21:38
Operator ID	DS
Elapsed Time	00:05:00
Mean Diam.	8.8 nm
Rel. Var.	0.046
Skew	3.016

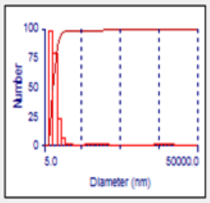

d(nm)	G(d)	C(d)	d(nm)	G(d)	C(d)	d(nm)	G(d)	C(d)
4.5	0	0	63.9	0	100	901.5	0	100
5.8	0	0	81.3	0	100	1146.8	0	100
7.3	100	48	103.4	0	100	1458.7	0	100
9.3	79	86	131.5	0	100	1855.6	0	100
11.9	23	97	167.3	0	100	2360.4	0	100
15.1	5	100	212.8	0	100	3002.5	0	100
19.2	0	100	270.7	0	100	3819.4	0	100
24.4	0	100	344.3	0	100	4858.4	0	100
31.0	0	100	438.0	0	100	6180.1	0	100
39.5	0	100	557.1	0	100	7861.4	0	100
50.2	0	100	708.7	0	100	10000.0	0	100

Number

Sample ID	ChaABC-Gal3 (Combined)
Date - Time	Jun 1, 2018 11:29:08
Operator ID	DS
Elapsed Time	00:05:00
Mean Diam.	7.5 nm
Rel. Var.	0.026
Skew	11.255

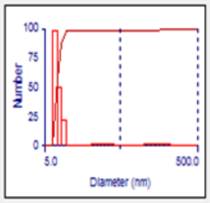

d(nm)	G(d)	C(d)	d(nm)	G(d)	C(d)	d(nm)	G(d)	C(d)
4.7	0	0	19.5	0	100	81.7	0	100
5.3	0	0	21.7	0	100	93.0	0	100
6.1	0	0	24.3	0	100	105.9	0	100
6.9	100	58	27.2	0	100	120.6	0	100
7.9	50	87	30.4	0	100	137.3	0	100
9.0	21	100	33.9	0	100	156.4	0	100
10.2	0	100	37.9	0	100	178.1	0	100
11.6	0	100	42.4	0	100	202.8	0	100
13.2	0	100	55.3	0	100	231.0	0	100
15.1	0	100	63.0	0	100	263.0	0	100
17.2	0	100	71.7	0	100	299.5	0	100

Number

Sample ID	ChaABC-Gal3 (Combined)
Date - Time	Jun 1, 2018 11:34:27
Operator ID	DS
Elapsed Time	00:05:00
Mean Diam.	5.6 nm
Rel. Var.	0.082
Skew	6.784

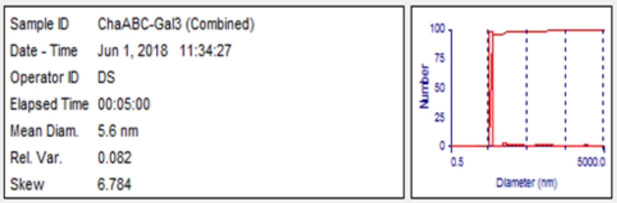

d(nm)	G(d)	C(d)	d(nm)	G(d)	C(d)	d(nm)	G(d)	C(d)
2.8	0	0	29.3	0	100	303.8	0	100
3.5	0	0	36.3	0	100	375.7	0	100
4.3	0	0	44.8	0	100	464.7	0	100
5.4	100	97	55.5	0	100	574.8	0	100
6.6	0	97	68.6	0	100	711.0	0	100
8.2	0	97	84.8	0	100	879.4	0	100
10.1	0	97	104.9	0	100	1087.7	0	100
12.5	2	99	129.8	0	100	1345.3	0	100
15.5	1	100	160.6	0	100	1663.9	0	100
19.2	0	100	198.6	0	100	2058.0	0	100
23.7	0	100	245.6	0	100	2545.5	0	100

Number

Sample ID	ChABC-Gal3 (Combined)
Date - Time	Jun 1, 2018 12:35:50
Operator ID	DS
Elapsed Time	00:05:00
Mean Diam.	9.5 nm
Rel. Var.	0.035
Skew	5.681

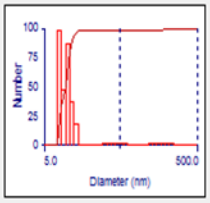

d(nm)	G(d)	C(d)	d(nm)	G(d)	C(d)	d(nm)	G(d)	C(d)
6.1	0	0	24.0	0	100	93.8	0	100
7.0	0	0	27.2	0	100	106.1	0	100
7.9	100	34	30.7	0	100	120.1	0	100
8.9	48	51	34.8	0	100	136.0	0	100
10.1	88	81	39.4	0	100	153.9	0	100
11.4	37	94	44.6	0	100	174.2	0	100
12.9	17	100	50.5	0	100	197.2	0	100
14.6	0	100	57.1	0	100	223.2	0	100
16.6	0	100	64.7	0	100	252.6	0	100
18.7	0	100	73.2	0	100	286.0	0	100
21.2	0	100	82.8	0	100	323.7	0	100

Number

Supplementary Figure 45. Number-weighted size distribution of ChABC-G3 in PBS. Data are technical replicates.

Sample ID ChaABC-Gal3 (Combined)
Date - Time Jun 1, 2018 11:08:17
Operator ID DS
Elapsed Time 00:05:00
Mean Diam. 529.1 nm
Rel. Var. 12.091
Skew 4.138

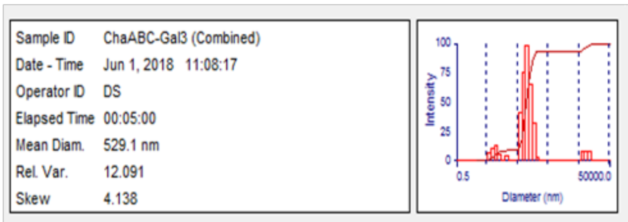

d(nm)	G(d)	C(d)	d(nm)	G(d)	C(d)	d(nm)	G(d)	C(d)
2.6	0	0	44.5	0	9	758.8	0	94
3.4	0	0	57.6	41	20	982.0	0	94
4.4	0	0	74.5	76	41	1270.9	0	94
5.7	6	2	96.4	100	68	1644.7	0	94
7.3	10	4	124.8	66	85	2128.5	0	94
9.5	12	8	161.5	31	94	2754.6	0	94
12.3	4	9	209.0	2	94	3564.9	0	94
15.9	0	9	270.5	0	94	4613.6	0	94
20.5	3	9	350.1	0	94	5970.7	7	96
26.6	0	9	453.0	0	94	7727.0	7	98
34.4	0	9	586.3	0	94	10000.0	7	100

Intensity

Sample ID ChaABC-Gal3 (Combined)
Date - Time Jun 1, 2018 11:21:38
Operator ID DS
Elapsed Time 00:05:00
Mean Diam. 489.4 nm
Rel. Var. 11.076
Skew 4.304

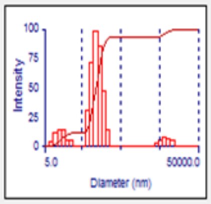

d(nm)	G(d)	C(d)	d(nm)	G(d)	C(d)	d(nm)	G(d)	C(d)
4.5	0	0	63.9	31	19	901.5	0	94
5.8	0	0	81.3	72	36	1146.8	0	94
7.3	3	1	103.4	100	59	1458.7	0	94
9.3	11	3	131.5	87	80	1855.6	0	94
11.9	14	7	167.3	48	91	2360.4	0	94
15.1	13	10	212.8	14	94	3002.5	0	94
19.2	5	11	270.7	0	94	3819.4	2	95
24.4	5	12	344.3	0	94	4858.4	4	96
31.0	0	12	438.0	0	94	6180.1	8	98
39.5	0	12	557.1	0	94	7861.4	6	99
50.2	0	12	708.7	0	94	10000.0	4	100

Intensity

Sample ID ChaABC-Gal3 (Combined)
Date - Time Jun 1, 2018 11:29:08
Operator ID DS
Elapsed Time 00:05:00
Mean Diam. 114.9 nm
Rel. Var. 0.187
Skew -0.928

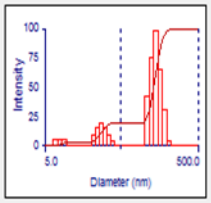

d(nm)	G(d)	C(d)	d(nm)	G(d)	C(d)	d(nm)	G(d)	C(d)
4.7	0	0	19.5	0	4	81.7	0	20
5.3	0	0	21.7	8	5	93.0	0	20
6.1	0	0	24.3	15	9	105.9	42	31
6.9	5	1	27.2	18	14	120.6	77	50
7.9	5	2	30.4	15	17	137.3	100	75
9.0	5	4	33.9	8	19	156.4	66	91
10.2	0	4	37.9	3	20	178.1	31	99
11.6	0	4	42.4	0	20	202.8	4	100
13.2	0	4	55.3	0	20	231.0	0	100
15.1	0	4	63.0	0	20	263.0	0	100
17.2	0	4	71.7	0	20	299.5	0	100

Intensity

Sample ID ChaABC-Gal3 (Combined)
Date - Time Jun 1, 2018 11:34:27
Operator ID DS
Elapsed Time 00:05:00
Mean Diam. 127.3 nm
Rel. Var. 1.547
Skew 8.563

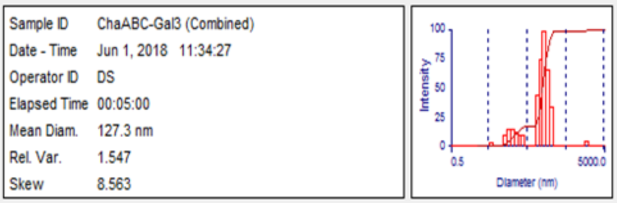

d(nm)	G(d)	C(d)	d(nm)	G(d)	C(d)	d(nm)	G(d)	C(d)
2.8	0	0	29.3	8	15	303.8	0	99
3.5	0	0	36.3	8	17	375.7	0	99
4.3	0	0	44.8	0	17	464.7	0	99
5.4	3	1	55.5	0	17	574.8	0	99
6.6	0	1	68.6	0	17	711.0	0	99
8.2	0	1	84.8	44	28	879.4	0	99
10.1	0	1	104.9	74	47	1087.7	0	99
12.5	9	3	129.8	100	73	1345.3	0	99
15.5	13	6	160.6	66	90	1663.9	4	100
19.2	13	10	198.6	33	99	2058.0	0	100
23.7	11	13	245.6	0	99	2545.5	0	100

Intensity

Sample ID ChABC-Gal3 (Combined)
Date - Time Jun 1, 2018 12:35:50
Operator ID DS
Elapsed Time 00:05:00
Mean Diam. 123.9 nm
Rel. Var. 0.238
Skew -0.583

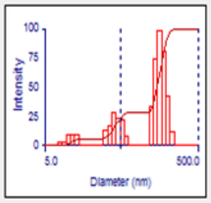

d(nm)	G(d)	C(d)	d(nm)	G(d)	C(d)	d(nm)	G(d)	C(d)
6.1	0	0	24.0	0	6	93.8	0	29
7.0	0	0	27.2	0	6	106.1	0	29
7.9	2	0	30.7	12	9	120.1	32	35
8.9	2	1	34.8	17	12	136.0	74	51
10.1	9	3	39.4	28	18	153.9	100	72
11.4	8	4	44.6	23	23	174.2	82	89
12.9	8	6	50.5	21	27	197.2	42	98
14.6	0	6	57.1	7	29	223.2	11	100
16.6	0	6	64.7	0	29	252.6	0	100
18.7	0	6	73.2	0	29	286.0	0	100
21.2	0	6	82.8	0	29	323.7	0	100

Intensity

Supplementary Figure 46. Intensity-weighted size distribution of ChABC-G3 in PBS. Data are technical replicates.

Sample ID ChaABC-Gal3 (Combined)
Date - Time Jun 1, 2018 11:08:17
Operator ID DS
Elapsed Time 00:05:00
Mean Diam. 9.3 nm
Rel. Var. 143.572
Skew 73.110

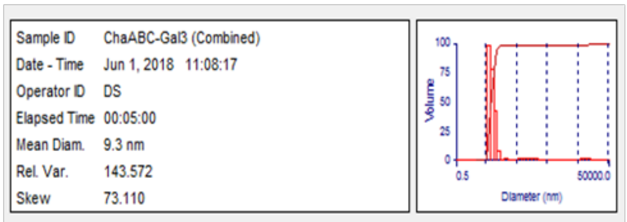

d(nm)	G(d)	C(d)	d(nm)	G(d)	C(d)	d(nm)	G(d)	C(d)
2.6	0	0	44.5	0	99	758.8	0	100
3.4	0	0	57.6	1	99	982.0	0	100
4.4	0	0	74.5	1	100	1270.9	0	100
5.7	100	44	96.4	0	100	1644.7	0	100
7.3	78	78	124.8	0	100	2128.5	0	100
9.5	42	96	161.5	0	100	2754.6	0	100
12.3	7	99	209.0	0	100	3564.9	0	100
15.9	0	99	270.5	0	100	4613.6	0	100
20.5	1	99	350.1	0	100	5970.7	0	100
26.6	0	99	453.0	0	100	7727.0	0	100
34.4	0	99	586.3	0	100	10000.0	0	100

Volume

Sample ID ChaABC-Gal3 (Combined)
Date - Time Jun 1, 2018 11:21:38
Operator ID DS
Elapsed Time 00:05:00
Mean Diam. 21.5 nm
Rel. Var. 132.330
Skew 26.285

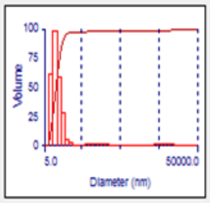

d(nm)	G(d)	C(d)	d(nm)	G(d)	C(d)	d(nm)	G(d)	C(d)
4.5	0	0	63.9	1	99	901.5	0	100
5.8	0	0	81.3	1	99	1146.8	0	100
7.3	61	24	103.4	1	100	1458.7	0	100
9.3	100	62	131.5	0	100	1855.6	0	100
11.9	59	85	167.3	0	100	2360.4	0	100
15.1	28	96	212.8	0	100	3002.5	0	100
19.2	5	98	270.7	0	100	3819.4	0	100
24.4	2	99	344.3	0	100	4858.4	0	100
31.0	0	99	438.0	0	100	6180.1	0	100
39.5	0	99	557.1	0	100	7861.4	0	100
50.2	0	99	708.7	0	100	10000.0	0	100

Volume

Sample ID ChaABC-Gal3 (Combined)
Date - Time Jun 1, 2018 11:29:08
Operator ID DS
Elapsed Time 00:05:00
Mean Diam. 10.2 nm
Rel. Var. 1.095
Skew 8.721

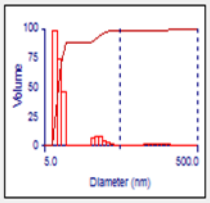

d(nm)	G(d)	C(d)	d(nm)	G(d)	C(d)	d(nm)	G(d)	C(d)
4.7	0	0	19.5	0	89	81.7	0	99
5.3	0	0	21.7	6	91	93.0	0	99
6.1	0	0	24.3	8	94	105.9	0	100
6.9	100	40	27.2	7	97	120.6	0	100
7.9	75	71	30.4	4	99	137.3	0	100
9.0	46	89	33.9	1	99	156.4	0	100
10.2	0	89	37.9	0	99	178.1	0	100
11.6	0	89	42.4	0	99	202.8	0	100
13.2	0	89	55.3	0	99	231.0	0	100
15.1	0	89	63.0	0	99	263.0	0	100
17.2	0	89	71.7	0	99	299.5	0	100

Volume

Sample ID ChaABC-Gal3 (Combined)
Date - Time Jun 1, 2018 11:34:27
Operator ID DS
Elapsed Time 00:05:00
Mean Diam. 10.9 nm
Rel. Var. 3.756
Skew 55.099

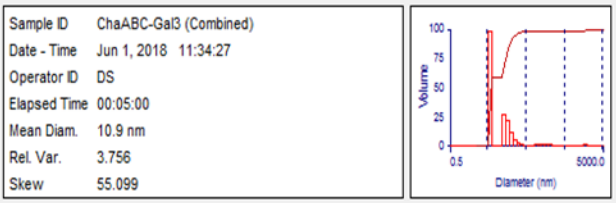

d(nm)	G(d)	C(d)	d(nm)	G(d)	C(d)	d(nm)	G(d)	C(d)
2.8	0	0	29.3	2	98	303.8	0	100
3.5	0	0	36.3	1	99	375.7	0	100
4.3	0	0	44.8	0	99	464.7	0	100
5.4	100	59	55.5	0	99	574.8	0	100
6.6	0	59	68.6	0	99	711.0	0	100
8.2	0	59	84.8	0	99	879.4	0	100
10.1	0	59	104.9	0	100	1087.7	0	100
12.5	27	75	129.8	0	100	1345.3	0	100
15.5	22	88	160.6	0	100	1663.9	0	100
19.2	11	94	198.6	0	100	2058.0	0	100
23.7	5	97	245.6	0	100	2545.5	0	100

Volume

Sample ID ChABC-Gal3 (Combined)
Date - Time Jun 1, 2018 12:35:50
Operator ID DS
Elapsed Time 00:05:00
Mean Diam. 12.8 nm
Rel. Var. 0.963
Skew 8.359

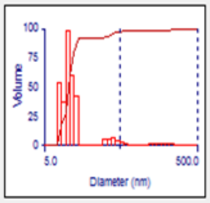

d(nm)	G(d)	C(d)	d(nm)	G(d)	C(d)	d(nm)	G(d)	C(d)
6.1	0	0	24.0	0	93	93.8	0	99
7.0	0	0	27.2	0	93	106.1	0	99
7.9	54	17	30.7	5	95	120.1	0	100
8.9	37	29	34.8	5	96	136.0	0	100
10.1	100	61	39.4	5	98	153.9	0	100
11.4	60	80	44.6	3	99	174.2	0	100
12.9	42	93	50.5	2	99	197.2	0	100
14.6	0	93	57.1	0	99	223.2	0	100
16.6	0	93	64.7	0	99	252.6	0	100
18.7	0	93	73.2	0	99	286.0	0	100
21.2	0	93	82.8	0	99	323.7	0	100

Volume

Supplementary Figure 47. Volume-weighted size distribution of ChABC-G3 in PBS. Data are technical replicates.

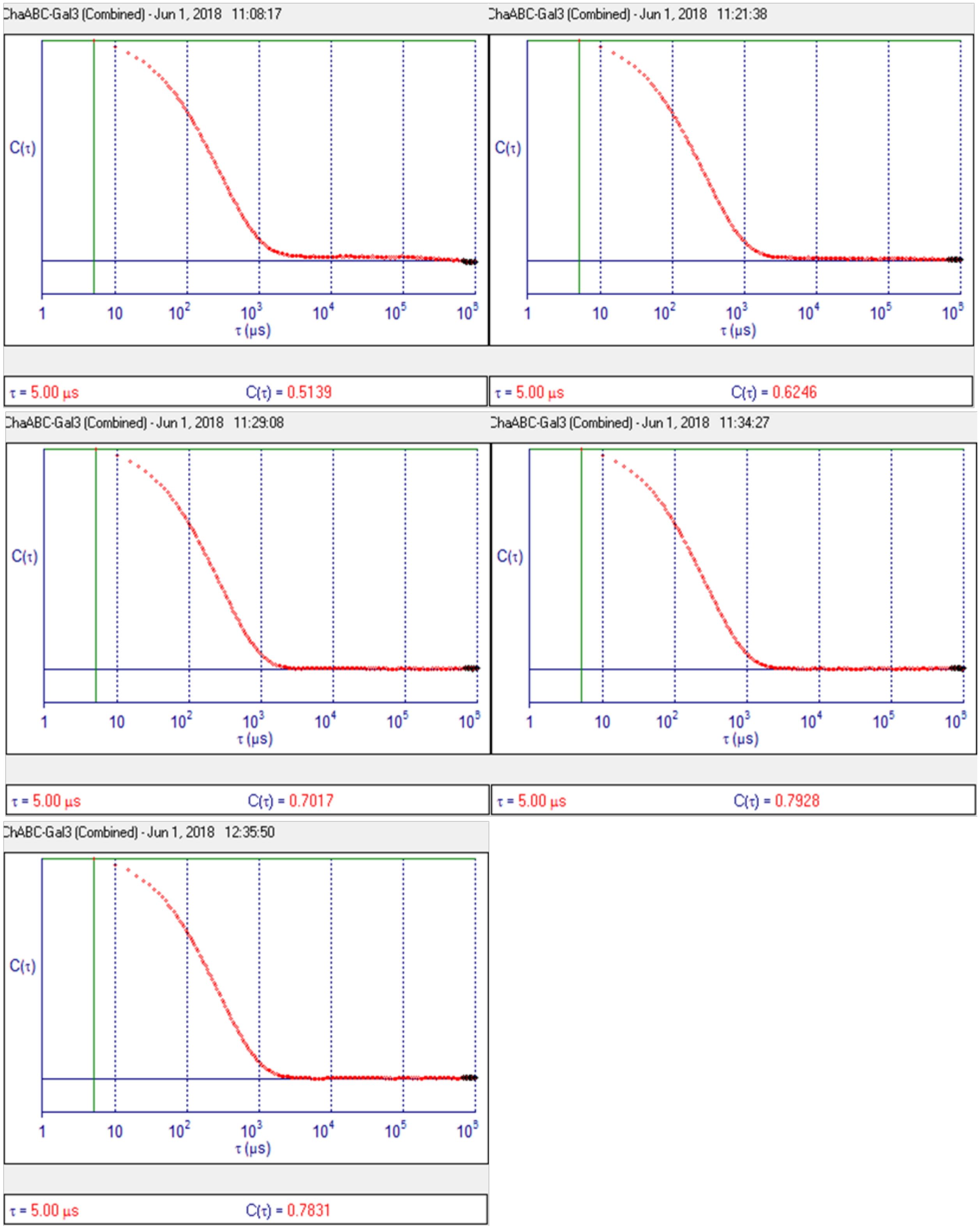

Supplementary Figure 48. Correlation functions for DLS measurements of ChABC-G3. Data are technical replicates.

Sample ID	ChABC-TT-Gal3 (Combined)
Date - Time	Jun 8, 2018 15:01:10
Operator ID	DS
Elapsed Time	00:05:00
Mean Diam.	17.2 nm
Rel. Var.	0.037
Skew	3.952

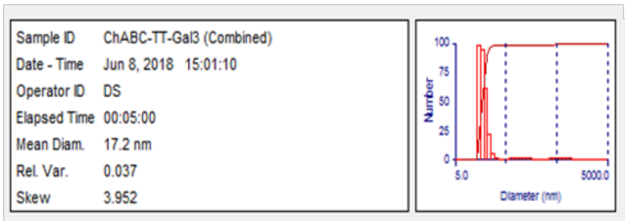

d(nm)	G(d)	C(d)	d(nm)	G(d)	C(d)	d(nm)	G(d)	C(d)
7.3	0	0	44.9	0	100	275.5	0	100
8.6	0	0	53.0	0	100	324.9	0	100
10.2	0	0	62.5	0	100	383.1	0	100
12.0	0	0	73.7	0	100	451.7	0	100
14.2	100	35	86.9	0	100	532.6	0	100
16.7	95	69	102.5	0	100	628.1	0	100
19.7	62	91	120.8	0	100	740.6	0	100
23.2	21	98	142.5	0	100	873.3	0	100
27.4	5	100	168.0	0	100	1029.8	0	100
32.3	0	100	198.1	0	100	1214.3	0	100
38.1	0	100	233.6	0	100	1431.9	0	100

Number

Sample ID	ChABC-TT-Gal3 (Combined)
Date - Time	Jun 8, 2018 15:06:29
Operator ID	DS
Elapsed Time	00:05:00
Mean Diam.	17.9 nm
Rel. Var.	0.037
Skew	2.894

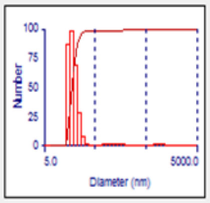

d(nm)	G(d)	C(d)	d(nm)	G(d)	C(d)	d(nm)	G(d)	C(d)
7.5	0	0	45.7	0	100	279.9	0	100
8.8	0	0	53.9	0	100	330.1	0	100
10.4	0	0	63.5	0	100	389.2	0	100
12.2	0	0	74.9	0	100	459.0	0	100
14.4	88	30	88.3	0	100	541.2	0	100
17.0	100	64	104.1	0	100	638.2	0	100
20.0	69	88	122.8	0	100	752.5	0	100
23.6	27	97	144.8	0	100	887.4	0	100
27.9	7	100	170.7	0	100	1046.4	0	100
32.8	1	100	201.3	0	100	1233.9	0	100
38.7	0	100	237.4	0	100	1454.9	0	100

Number

Sample ID	ChABC-TT-Gal3 (Combined)
Date - Time	Jun 8, 2018 15:23:54
Operator ID	DS
Elapsed Time	00:05:00
Mean Diam.	20.7 nm
Rel. Var.	0.013
Skew	5.801

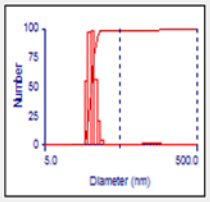

d(nm)	G(d)	C(d)	d(nm)	G(d)	C(d)	d(nm)	G(d)	C(d)
12.3	0	0	33.5	0	100	91.7	0	100
13.4	0	0	36.7	0	100	100.5	0	100
14.7	0	0	40.2	0	100	110.1	0	100
16.1	0	0	44.1	0	100	120.6	0	100
17.7	55	17	48.3	0	100	132.2	0	100
19.4	98	46	53.0	0	100	144.9	0	100
21.2	100	76	58.0	0	100	158.7	0	100
23.2	57	93	63.6	0	100	174.0	0	100
25.5	20	99	69.7	0	100	190.6	0	100
27.9	3	100	76.4	0	100	208.9	0	100
30.6	0	100	83.7	0	100	228.9	0	100

Number

Sample ID	ChABC-TT-Gal3 (Combined)
Date - Time	Jun 8, 2018 15:33:08
Operator ID	DS
Elapsed Time	00:05:00
Mean Diam.	21.9 nm
Rel. Var.	0.010
Skew	8.439

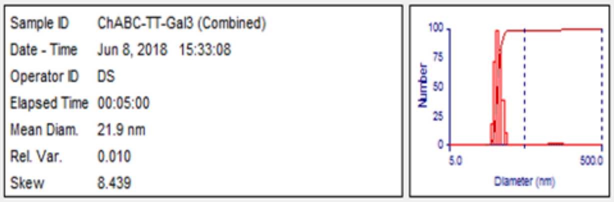

d(nm)	G(d)	C(d)	d(nm)	G(d)	C(d)	d(nm)	G(d)	C(d)
15.7	0	0	36.9	0	100	86.7	0	100
17.0	0	0	39.9	0	100	93.7	0	100
18.3	18	6	43.1	0	100	101.3	0	100
19.8	72	28	46.6	0	100	109.4	0	100
21.4	100	59	50.3	0	100	118.3	0	100
23.1	83	85	54.4	0	100	127.8	0	100
25.0	38	97	58.8	0	100	138.2	0	100
27.0	10	100	63.5	0	100	149.3	0	100
29.2	0	100	68.7	0	100	161.4	0	100
31.6	0	100	74.2	0	100	174.4	0	100
34.1	0	100	80.2	0	100	188.5	0	100

Number

Sample ID	ChABC-TT-Gal3 (Combined)
Date - Time	Jun 8, 2018 15:38:23
Operator ID	DS
Elapsed Time	00:05:00
Mean Diam.	18.5 nm
Rel. Var.	0.041
Skew	3.331

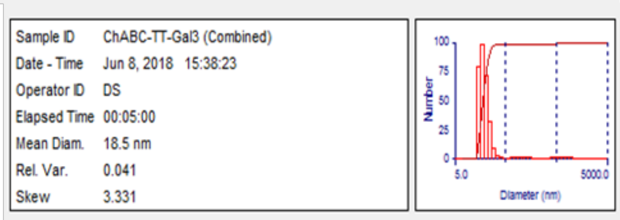

d(nm)	G(d)	C(d)	d(nm)	G(d)	C(d)	d(nm)	G(d)	C(d)
7.6	0	0	46.6	0	100	285.7	0	100
9.0	0	0	55.0	0	100	336.9	0	100
10.6	0	0	64.8	0	100	397.3	0	100
12.5	0	0	76.4	0	100	468.5	0	100
14.7	80	27	90.1	0	100	552.4	0	100
17.3	100	61	106.3	0	100	651.4	0	100
20.4	72	86	125.3	0	100	768.1	0	100
24.1	32	96	147.8	0	100	905.8	0	100
28.4	9	99	174.3	0	100	1068.1	0	100
33.5	1	100	205.5	0	100	1259.4	0	100
39.5	0	100	242.3	0	100	1485.1	0	100

Number

Sample ID	ChABC-TT-Gal3 (Combined)
Date - Time	Jun 8, 2018 15:43:37
Operator ID	DS
Elapsed Time	00:05:00
Mean Diam.	21.7 nm
Rel. Var.	0.014
Skew	5.889

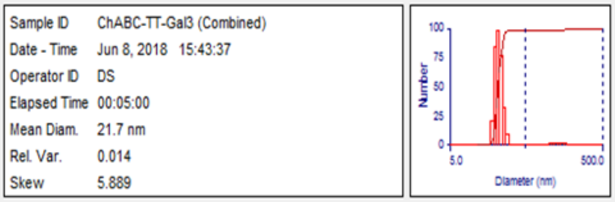

d(nm)	G(d)	C(d)	d(nm)	G(d)	C(d)	d(nm)	G(d)	C(d)
12.3	0	0	33.7	0	100	92.2	0	100
13.5	0	0	36.9	0	100	101.0	0	100
14.8	0	0	40.5	0	100	110.7	0	100
16.2	0	0	44.3	0	100	121.3	0	100
17.8	21	6	48.6	0	100	132.9	0	100
19.5	85	33	53.3	0	100	145.7	0	100
21.3	100	64	58.4	0	100	159.6	0	100
23.4	78	88	63.9	0	100	174.9	0	100
25.6	31	98	70.1	0	100	191.7	0	100
28.1	8	100	76.8	0	100	210.0	0	100
30.8	0	100	84.1	0	100	230.2	0	100

Number

Supplementary Figure 49. Number-weighted size distribution of ChABC-TT-G3 in PBS. Data are technical replicates.

Sample ID	ChABC-TT-Gal3 (Combined)
Date - Time	Jun 8, 2018 15:01:10
Operator ID	DS
Elapsed Time	00:05:00
Mean Diam.	23.8 nm
Rel. Var.	2.790
Skew	12.148

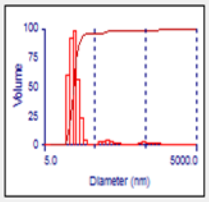

d(nm)	G(d)	C(d)	d(nm)	G(d)	C(d)	d(nm)	G(d)	C(d)
7.3	0	0	44.9	0	97	275.5	0	99
8.6	0	0	53.0	0	97	324.9	0	99
10.2	0	0	62.5	2	97	383.1	0	99
12.0	0	0	73.7	2	98	451.7	2	100
14.2	60	17	86.9	3	99	532.6	0	100
16.7	93	44	102.5	1	99	628.1	0	100
19.7	100	73	120.8	1	99	740.6	0	100
23.2	56	89	142.5	0	99	873.3	0	100
27.4	22	96	168.0	0	99	1029.8	0	100
32.3	3	97	198.1	0	99	1214.3	0	100
38.1	0	97	233.6	0	99	1431.9	0	100

Volume

Sample ID	ChABC-TT-Gal3 (Combined)
Date - Time	Jun 8, 2018 15:06:29
Operator ID	DS
Elapsed Time	00:05:00
Mean Diam.	23.4 nm
Rel. Var.	3.361
Skew	19.033

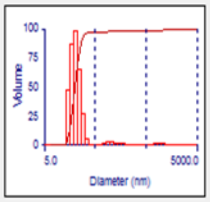

d(nm)	G(d)	C(d)	d(nm)	G(d)	C(d)	d(nm)	G(d)	C(d)
7.5	0	0	45.7	0	98	279.9	0	100
8.8	0	0	53.9	0	98	330.1	0	100
10.4	0	0	63.5	0	98	389.2	0	100
12.2	0	0	74.9	1	98	459.0	0	100
14.4	48	14	88.3	2	99	541.2	0	100
17.0	88	40	104.1	2	99	638.2	0	100
20.0	100	70	122.8	1	100	752.5	0	100
23.6	65	89	144.8	0	100	887.4	0	100
27.9	27	97	170.7	0	100	1046.4	0	100
32.8	5	98	201.3	0	100	1233.9	0	100
38.7	0	98	237.4	0	100	1454.9	0	100

Volume

Sample ID	ChABC-TT-Gal3 (Combined)
Date - Time	Jun 8, 2018 15:23:54
Operator ID	DS
Elapsed Time	00:05:00
Mean Diam.	22.9 nm
Rel. Var.	0.269
Skew	7.938

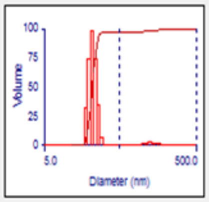

d(nm)	G(d)	C(d)	d(nm)	G(d)	C(d)	d(nm)	G(d)	C(d)
12.3	0	0	33.5	0	99	91.7	0	99
13.4	0	0	36.7	0	99	100.5	1	99
14.7	0	0	40.2	0	99	110.1	1	99
16.1	0	0	44.1	0	99	120.6	1	100
17.7	32	10	48.3	0	99	132.2	1	100
19.4	74	32	53.0	0	99	144.9	0	100
21.2	100	63	58.0	0	99	158.7	0	100
23.2	75	86	63.6	0	99	174.0	0	100
25.5	34	97	69.7	0	99	190.6	0	100
27.9	6	99	76.4	0	99	208.9	0	100
30.6	0	99	83.7	0	99	228.9	0	100

Volume

Sample ID	ChABC-TT-Gal3 (Combined)
Date - Time	Jun 8, 2018 15:33:08
Operator ID	DS
Elapsed Time	00:05:00
Mean Diam.	24.0 nm
Rel. Var.	0.264
Skew	7.659

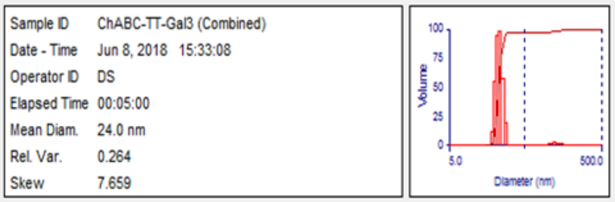

d(nm)	G(d)	C(d)	d(nm)	G(d)	C(d)	d(nm)	G(d)	C(d)
15.7	0	0	36.9	0	98	86.7	0	98
17.0	0	0	39.9	0	98	93.7	0	98
18.3	11	3	43.1	0	98	101.3	0	99
19.8	55	19	46.6	0	98	109.4	1	99
21.4	95	47	50.3	0	98	118.3	2	99
23.1	100	76	54.4	0	98	127.8	1	100
25.0	58	93	58.8	0	98	138.2	1	100
27.0	19	98	63.5	0	98	149.3	0	100
29.2	0	98	68.7	0	98	161.4	0	100
31.6	0	98	74.2	0	98	174.4	0	100
34.1	0	98	80.2	0	98	188.5	0	100

Volume

Sample ID	ChABC-TT-Gal3 (Combined)
Date - Time	Jun 8, 2018 15:38:23
Operator ID	DS
Elapsed Time	00:05:00
Mean Diam.	25.7 nm
Rel. Var.	2.664
Skew	11.944

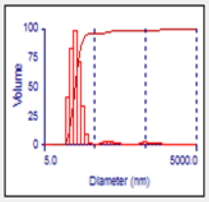

d(nm)	G(d)	C(d)	d(nm)	G(d)	C(d)	d(nm)	G(d)	C(d)
7.6	0	0	46.6	0	97	285.7	0	99
9.0	0	0	55.0	0	97	336.9	0	99
10.6	0	0	64.8	0	97	397.3	0	99
12.5	0	0	76.4	2	98	468.5	1	100
14.7	41	12	90.1	2	98	552.4	0	100
17.3	84	36	106.3	2	99	651.4	0	100
20.4	100	64	125.3	1	99	768.1	0	100
24.1	72	85	147.8	0	99	905.8	0	100
28.4	34	94	174.3	0	99	1068.1	0	100
33.5	9	97	205.5	0	99	1259.4	0	100
39.5	1	97	242.3	0	99	1485.1	0	100

Volume

Sample ID	ChABC-TT-Gal3 (Combined)
Date - Time	Jun 8, 2018 15:43:37
Operator ID	DS
Elapsed Time	00:05:00
Mean Diam.	24.0 nm
Rel. Var.	0.270
Skew	7.703

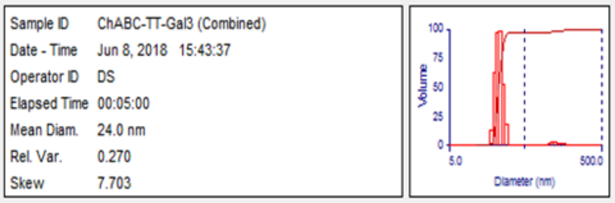

d(nm)	G(d)	C(d)	d(nm)	G(d)	C(d)	d(nm)	G(d)	C(d)
12.3	0	0	33.7	0	98	92.2	0	98
13.5	0	0	36.9	0	98	101.0	1	99
14.8	0	0	40.5	0	98	110.7	1	99
16.2	0	0	44.3	0	98	121.3	2	100
17.8	12	3	48.6	0	98	132.9	1	100
19.5	63	22	53.3	0	98	145.7	0	100
21.3	98	50	58.4	0	98	159.6	0	100
23.4	100	78	63.9	0	98	174.9	0	100
25.6	53	93	70.1	0	98	191.7	0	100
28.1	18	98	76.8	0	98	210.0	0	100
30.8	0	98	84.1	0	98	230.2	0	100

Volume

Supplementary Figure 50. Volume-weighted size distribution of ChABC-TT-G3 in PBS. Data are technical replicates.

Sample ID	ChABC-TT-Gal3 (Combined)
Date - Time	Jun 8, 2018 15:01:10
Operator ID	DS
Elapsed Time	00:05:00
Mean Diam.	119.7 nm
Rel. Var.	1.635
Skew	2.624

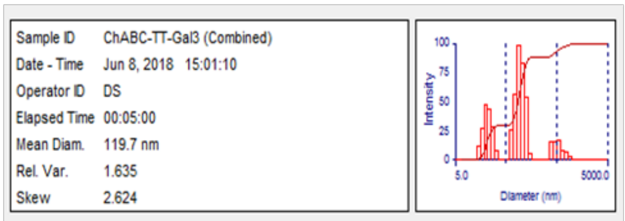

d(nm)	G(d)	C(d)	d(nm)	G(d)	C(d)	d(nm)	G(d)	C(d)
7.3	0	0	44.9	0	30	275.5	0	89
8.6	0	0	53.0	0	30	324.9	0	89
10.2	0	0	62.5	25	35	383.1	15	91
12.0	0	0	73.7	56	45	451.7	15	94
14.2	11	2	86.9	100	63	532.6	16	97
16.7	27	7	102.5	84	78	628.1	8	99
19.7	48	16	120.8	54	88	740.6	6	100
23.2	44	24	142.5	5	89	873.3	2	100
27.4	28	29	168.0	0	89	1029.8	0	100
32.3	7	30	198.1	0	89	1214.3	0	100
38.1	0	30	233.6	0	89	1431.9	0	100

Intensity

Sample ID	ChABC-TT-Gal3 (Combined)
Date - Time	Jun 8, 2018 15:06:29
Operator ID	DS
Elapsed Time	00:05:00
Mean Diam.	118.8 nm
Rel. Var.	2.252
Skew	4.018

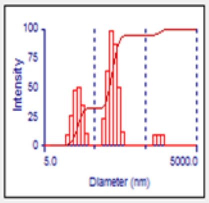

d(nm)	G(d)	C(d)	d(nm)	G(d)	C(d)	d(nm)	G(d)	C(d)
7.5	0	0	45.7	0	33	279.9	0	95
8.8	0	0	53.9	0	33	330.1	0	95
10.4	0	0	63.5	0	33	389.2	0	95
12.2	0	0	74.9	23	37	459.0	0	95
14.4	8	2	88.3	65	49	541.2	0	95
17.0	26	6	104.1	100	68	638.2	0	95
20.0	48	15	122.8	88	84	752.5	8	97
23.6	51	25	144.8	50	93	887.4	9	98
27.9	34	31	170.7	11	95	1046.4	8	100
32.8	9	33	201.3	0	95	1233.9	0	100
38.7	0	33	237.4	0	95	1454.9	0	100

Intensity

Sample ID	ChABC-TT-Gal3 (Combined)
Date - Time	Jun 8, 2018 15:23:54
Operator ID	DS
Elapsed Time	00:05:00
Mean Diam.	88.0 nm
Rel. Var.	0.309
Skew	-0.495

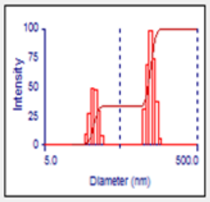

d(nm)	G(d)	C(d)	d(nm)	G(d)	C(d)	d(nm)	G(d)	C(d)
12.3	0	0	33.5	0	35	91.7	0	35
13.4	0	0	36.7	0	35	100.5	31	41
14.7	0	0	40.2	0	35	110.1	69	55
16.1	0	0	44.1	0	35	120.6	100	76
17.7	9	2	48.3	0	35	132.2	75	91
19.4	27	7	53.0	0	35	144.9	37	99
21.2	48	17	58.0	0	35	158.7	5	100
23.2	47	27	63.6	0	35	174.0	0	100
25.5	29	33	69.7	0	35	190.6	0	100
27.9	7	35	76.4	0	35	208.9	0	100
30.6	0	35	83.7	0	35	228.9	0	100

Intensity

Sample ID	ChABC-TT-Gal3 (Combined)
Date - Time	Jun 8, 2018 15:33:08
Operator ID	DS
Elapsed Time	00:05:00
Mean Diam.	88.2 nm
Rel. Var.	0.302
Skew	-0.521

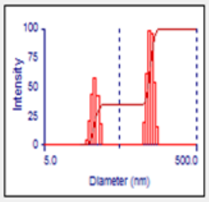

d(nm)	G(d)	C(d)	d(nm)	G(d)	C(d)	d(nm)	G(d)	C(d)
15.7	0	0	36.9	0	35	86.7	0	35
17.0	0	0	39.9	0	35	93.7	0	35
18.3	3	1	43.1	0	35	101.3	18	38
19.8	20	4	46.6	0	35	109.4	62	50
21.4	44	13	50.3	0	35	118.3	100	69
23.1	58	24	54.4	0	35	127.8	96	87
25.0	42	32	58.8	0	35	138.2	54	97
27.0	17	35	63.5	0	35	149.3	15	100
29.2	0	35	68.7	0	35	161.4	0	100
31.6	0	35	74.2	0	35	174.4	0	100
34.1	0	35	80.2	0	35	188.5	0	100

Intensity

Sample ID	ChABC-TT-Gal3 (Combined)
Date - Time	Jun 8, 2018 15:38:23
Operator ID	DS
Elapsed Time	00:05:00
Mean Diam.	134.4 nm
Rel. Var.	1.459
Skew	2.409

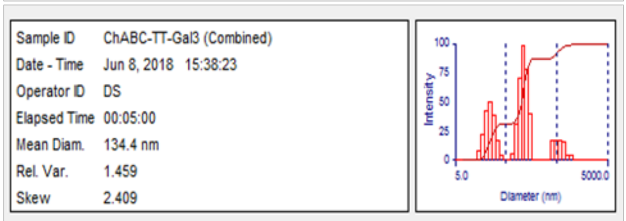

d(nm)	G(d)	C(d)	d(nm)	G(d)	C(d)	d(nm)	G(d)	C(d)
7.6	0	0	46.6	0	31	285.7	0	88
9.0	0	0	55.0	0	31	336.9	0	88
10.6	0	0	64.8	4	32	397.3	16	91
12.5	0	0	76.4	31	38	468.5	17	94
14.7	7	1	90.1	70	50	552.4	16	96
17.3	22	5	106.3	100	67	651.4	15	99
20.4	43	12	125.3	78	81	768.1	3	100
24.1	50	21	147.8	39	88	905.8	3	100
28.4	38	28	174.3	0	88	1068.1	0	100
33.5	16	31	205.5	0	88	1259.4	0	100
39.5	3	31	242.3	0	88	1485.1	0	100

Intensity

Sample ID	ChABC-TT-Gal3 (Combined)
Date - Time	Jun 8, 2018 15:43:37
Operator ID	DS
Elapsed Time	00:05:00
Mean Diam.	89.8 nm
Rel. Var.	0.310
Skew	-0.474

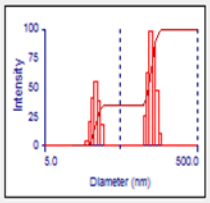

d(nm)	G(d)	C(d)	d(nm)	G(d)	C(d)	d(nm)	G(d)	C(d)
12.3	0	0	33.7	0	35	92.2	0	35
13.5	0	0	36.9	0	35	101.0	25	40
14.8	0	0	40.5	0	35	110.7	63	52
16.2	0	0	44.3	0	35	121.3	100	72
17.8	3	1	48.6	0	35	132.9	85	89
19.5	20	5	53.3	0	35	145.7	47	98
21.3	41	13	58.4	0	35	159.6	10	100
23.4	56	24	63.9	0	35	174.9	0	100
25.6	39	31	70.1	0	35	191.7	0	100
28.1	17	35	76.8	0	35	210.0	0	100
30.8	0	35	84.1	0	35	230.2	0	100

Intensity

Supplementary Figure 51. Intensity-weighted size distribution of ChABC-TT-G3 in PBS. Data are technical replicates.

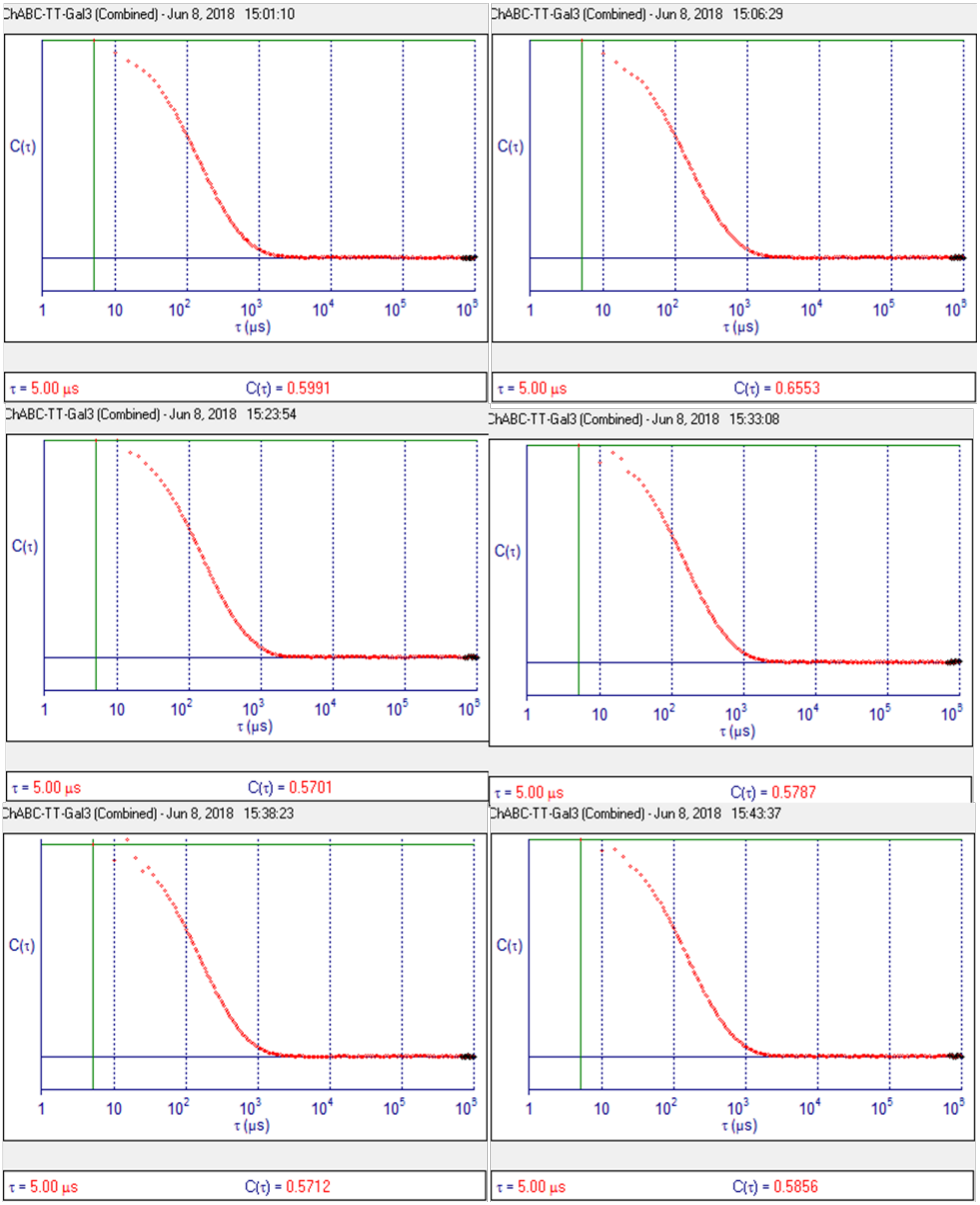

Supplementary Figure 52. Correlation functions for DLS measurements of ChABC-TT-G3. Data are technical replicates.

a

Sample ID	WT-G3 + ASF 2.5 uM (Combined)
Date - Time	Jul 16, 2018 17:38:32
Operator ID	SF
Elapsed Time	00:05:00
Mean Diam.	11.9 nm
Rel. Var.	0.027
Skew	9.683

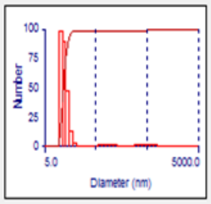

d(nm)	G(d)	C(d)	d(nm)	G(d)	C(d)	d(nm)	G(d)	C(d)
6.2	0	0	37.1	0	100	221.6	0	100
7.3	0	0	43.7	0	100	260.7	0	100
8.6	0	0	51.4	0	100	306.6	0	100
10.1	100	40	60.5	0	100	360.7	0	100
11.9	90	75	71.1	0	100	424.3	0	100
14.0	47	94	83.7	0	100	499.1	0	100
16.5	13	99	98.4	0	100	587.1	0	100
19.4	2	100	115.8	0	100	690.6	0	100
22.8	0	100	136.2	0	100	812.3	0	100
26.8	0	100	160.2	0	100	955.6	0	100
31.6	0	100	188.4	0	100	1124.0	0	100

Number

Sample ID	WT-G3 + ASF 2.5 uM (Combined)
Date - Time	Jul 16, 2018 17:45:46
Operator ID	SF
Elapsed Time	00:05:00
Mean Diam.	11.3 nm
Rel. Var.	0.034
Skew	7.081

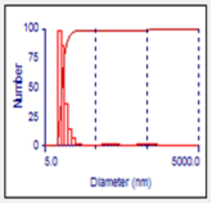

d(nm)	G(d)	C(d)	d(nm)	G(d)	C(d)	d(nm)	G(d)	C(d)
7.1	0	0	38.5	0	100	207.7	0	100
8.3	0	0	44.8	0	100	242.1	0	100
9.7	100	41	52.3	0	100	282.2	0	100
11.3	86	77	60.9	0	100	328.9	0	100
13.1	36	92	71.0	0	100	383.4	0	100
15.3	14	97	82.8	0	100	446.9	0	100
17.9	5	99	96.5	0	100	521.0	0	100
20.8	1	100	112.5	0	100	607.3	0	100
24.3	0	100	131.1	0	100	707.9	0	100
28.3	0	100	152.8	0	100	825.2	0	100
33.0	0	100	178.1	0	100	962.0	0	100

Number

Sample ID	WT-G3 + ASF 2.5 uM (Combined)
Date - Time	Jul 16, 2018 17:55:10
Operator ID	SF
Elapsed Time	00:05:00
Mean Diam.	9.6 nm
Rel. Var.	0.021
Skew	8.189

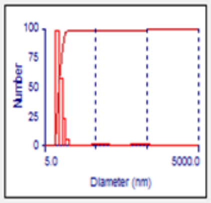

d(nm)	G(d)	C(d)	d(nm)	G(d)	C(d)	d(nm)	G(d)	C(d)
5.3	0	0	31.5	0	100	188.0	0	100
6.2	0	0	37.1	0	100	221.1	0	100
7.3	0	0	43.6	0	100	260.1	0	100
8.6	100	54	51.3	0	100	305.9	0	100
10.1	58	85	60.3	0	100	359.9	0	100
11.9	22	98	70.9	0	100	423.3	0	100
14.0	5	100	83.5	0	100	497.9	0	100
16.5	0	100	98.2	0	100	585.7	0	100
19.4	0	100	115.5	0	100	689.0	0	100
22.8	0	100	135.8	0	100	810.5	0	100
26.8	0	100	159.8	0	100	953.3	0	100

Number

b

Sample ID	WT-G3 + ASF 2.5 uM (Combined)
Date - Time	Jul 16, 2018 17:38:32
Operator ID	SF
Elapsed Time	00:05:00
Mean Diam.	27.8 nm
Rel. Var.	8.388
Skew	5.505

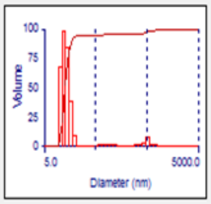

d(nm)	G(d)	C(d)	d(nm)	G(d)	C(d)	d(nm)	G(d)	C(d)
6.2	0	0	37.1	0	96	221.6	0	97
7.3	0	0	43.7	0	96	260.7	0	97
8.6	0	0	51.4	0	96	306.6	0	97
10.1	68	22	60.5	0	96	360.7	0	97
11.9	100	54	71.1	1	96	424.3	2	98
14.0	85	81	83.7	1	97	499.1	7	100
16.5	38	93	98.4	1	97	587.1	0	100
19.4	9	96	115.8	0	97	690.6	0	100
22.8	0	96	136.2	0	97	812.3	0	100
26.8	0	96	160.2	0	97	955.6	0	100
31.6	0	96	188.4	0	97	1124.0	0	100

Volume

Sample ID	WT-G3 + ASF 2.5 uM (Combined)
Date - Time	Jul 16, 2018 17:45:46
Operator ID	SF
Elapsed Time	00:05:00
Mean Diam.	24.7 nm
Rel. Var.	8.556
Skew	6.216

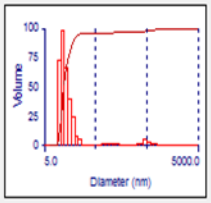

d(nm)	G(d)	C(d)	d(nm)	G(d)	C(d)	d(nm)	G(d)	C(d)
7.1	0	0	38.5	0	97	207.7	0	98
8.3	0	0	44.8	0	97	242.1	0	98
9.7	73	22	52.3	0	97	282.2	0	98
11.3	100	53	60.9	0	97	328.9	0	98
13.1	67	73	71.0	1	97	383.4	0	98
15.3	40	86	82.8	1	97	446.9	5	99
17.9	24	93	96.5	1	97	521.0	2	100
20.8	8	95	112.5	0	98	607.3	0	100
24.3	5	97	131.1	0	98	707.9	0	100
28.3	0	97	152.8	0	98	825.2	0	100
33.0	0	97	178.1	0	98	962.0	0	100

Volume

Sample ID	WT-G3 + ASF 2.5 uM (Combined)
Date - Time	Jul 16, 2018 17:55:10
Operator ID	SF
Elapsed Time	00:05:00
Mean Diam.	15.8 nm
Rel. Var.	8.584
Skew	9.443

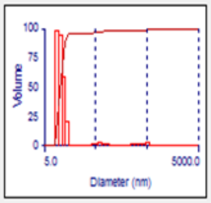

d(nm)	G(d)	C(d)	d(nm)	G(d)	C(d)	d(nm)	G(d)	C(d)
5.3	0	0	31.5	0	97	188.0	0	99
6.2	0	0	37.1	0	97	221.1	0	99
7.3	0	0	43.6	1	97	260.1	0	99
8.6	100	35	51.3	1	98	305.9	0	99
10.1	95	69	60.3	2	98	359.9	0	99
11.9	59	90	70.9	1	99	423.3	1	99
14.0	20	97	83.5	0	99	497.9	2	100
16.5	0	97	98.2	0	99	585.7	0	100
19.4	0	97	115.5	0	99	689.0	0	100
22.8	0	97	135.8	0	99	810.5	0	100
26.8	0	97	159.8	0	99	953.3	0	100

Volume

Supplementary Figure 53. Size distribution of 2.5 μM WT-G3 plus 7 μM ASF. **a** Number- and **b** volume-weighted. Data in columns are technical replicates of **a** or **b**.

a

Sample ID	WT-G3 + ASF 2.5 uM (Combined)
Date - Time	Jul 16, 2018 17:45:46
Operator ID	SF
Elapsed Time	00:05:00
Mean Diam.	12.6 nm
Rel. Var.	0.972
Skew	34.694

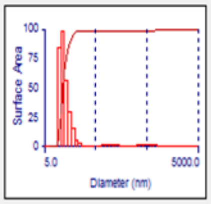

d(nm)	G(d)	C(d)	d(nm)	G(d)	C(d)	d(nm)	G(d)	C(d)
7.1	0	0	38.5	0	100	207.7	0	100
8.3	0	0	44.8	0	100	242.1	0	100
9.7	85	29	52.3	0	100	282.2	0	100
11.3	100	63	60.9	0	100	328.9	0	100
13.1	57	82	71.0	0	100	383.4	0	100
15.3	29	92	82.8	0	100	446.9	0	100
17.9	15	98	96.5	0	100	521.0	0	100
20.8	4	99	112.5	0	100	607.3	0	100
24.3	2	100	131.1	0	100	707.9	0	100
28.3	0	100	152.8	0	100	825.2	0	100
33.0	0	100	178.1	0	100	962.0	0	100

Surface Area

Sample ID	WT-G3 + ASF 2.5 uM (Combined)
Date - Time	Jul 16, 2018 17:38:32
Operator ID	SF
Elapsed Time	00:05:00
Mean Diam.	13.0 nm
Rel. Var.	1.129
Skew	31.817

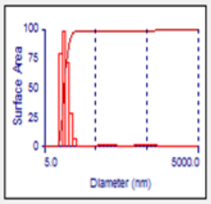

d(nm)	G(d)	C(d)	d(nm)	G(d)	C(d)	d(nm)	G(d)	C(d)
6.2	0	0	37.1	0	100	221.6	0	100
7.3	0	0	43.7	0	100	260.7	0	100
8.6	0	0	51.4	0	100	306.6	0	100
10.1	80	28	60.5	0	100	360.7	0	100
11.9	100	63	71.1	0	100	424.3	0	100
14.0	72	88	83.7	0	100	499.1	0	100
16.5	27	98	98.4	0	100	587.1	0	100
19.4	5	100	115.8	0	100	690.6	0	100
22.8	0	100	136.2	0	100	812.3	0	100
26.8	0	100	160.2	0	100	955.6	0	100
31.6	0	100	188.4	0	100	1124.0	0	100

Surface Area

Sample ID	WT-G3 + ASF 2.5 uM (Combined)
Date - Time	Jul 16, 2018 17:55:10
Operator ID	SF
Elapsed Time	00:05:00
Mean Diam.	10.2 nm
Rel. Var.	0.545
Skew	50.288

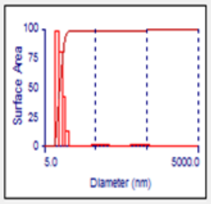

d(nm)	G(d)	C(d)	d(nm)	G(d)	C(d)	d(nm)	G(d)	C(d)
5.3	0	0	31.5	0	100	188.0	0	100
6.2	0	0	37.1	0	100	221.1	0	100
7.3	0	0	43.6	0	100	260.1	0	100
8.6	100	42	51.3	0	100	305.9	0	100
10.1	81	76	60.3	0	100	359.9	0	100
11.9	43	94	70.9	0	100	423.3	0	100
14.0	12	100	83.5	0	100	497.9	0	100
16.5	0	100	98.2	0	100	585.7	0	100
19.4	0	100	115.5	0	100	689.0	0	100
22.8	0	100	135.8	0	100	810.5	0	100
26.8	0	100	159.8	0	100	953.3	0	100

Surface Area

b

Sample ID	WT-G3 + ASF 2.5 uM (Combined)
Date - Time	Jul 16, 2018 17:45:46
Operator ID	SF
Elapsed Time	00:05:00
Mean Diam.	325.7 nm
Rel. Var.	0.431
Skew	-0.134

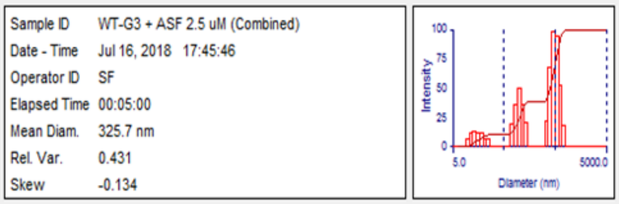

d(nm)	G(d)	C(d)	d(nm)	G(d)	C(d)	d(nm)	G(d)	C(d)
7.1	0	0	38.5	0	11	207.7	0	39
8.3	0	0	44.8	0	11	242.1	0	39
9.7	5	1	52.3	0	11	282.2	0	39
11.3	12	3	60.9	0	11	328.9	22	42
13.1	12	5	71.0	19	14	383.4	68	54
15.3	12	7	82.8	35	20	446.9	100	71
17.9	11	9	96.5	50	29	521.0	95	88
20.8	6	10	112.5	36	35	607.3	52	97
24.3	6	11	131.1	20	39	707.9	18	100
28.3	0	11	152.8	0	39	825.2	0	100
33.0	0	11	178.1	0	39	962.0	0	100

Intensity

Sample ID	WT-G3 + ASF 2.5 uM (Combined)
Date - Time	Jul 16, 2018 17:38:32
Operator ID	SF
Elapsed Time	00:05:00
Mean Diam.	306.2 nm
Rel. Var.	0.382
Skew	-0.209

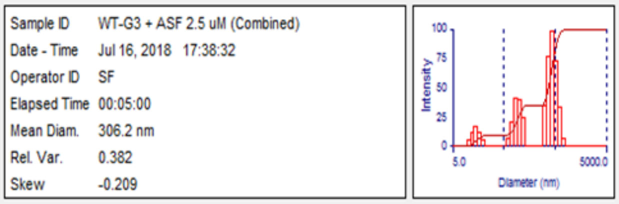

d(nm)	G(d)	C(d)	d(nm)	G(d)	C(d)	d(nm)	G(d)	C(d)
6.2	0	0	37.1	0	10	221.6	0	35
7.3	0	0	43.7	0	10	260.7	0	35
8.6	0	0	51.4	0	10	306.6	35	42
10.1	5	1	60.5	6	11	360.7	78	58
11.9	11	3	71.1	20	15	424.3	100	78
14.0	16	6	83.7	41	23	499.1	73	92
16.5	11	9	98.4	40	31	587.1	34	99
19.4	4	10	115.8	24	35	690.6	6	100
22.8	0	10	136.2	0	35	812.3	0	100
26.8	0	10	160.2	0	35	955.6	0	100
31.6	0	10	188.4	0	35	1124.0	0	100

Intensity

Sample ID	WT-G3 + ASF 2.5 uM (Combined)
Date - Time	Jul 16, 2018 17:55:10
Operator ID	SF
Elapsed Time	00:05:00
Mean Diam.	259.0 nm
Rel. Var.	0.355
Skew	-0.347

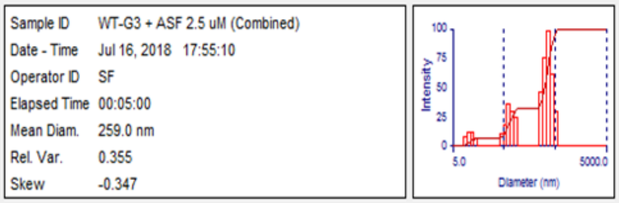

d(nm)	G(d)	C(d)	d(nm)	G(d)	C(d)	d(nm)	G(d)	C(d)
5.3	0	0	31.5	0	8	188.0	0	32
6.2	0	0	37.1	0	8	221.1	0	32
7.3	0	0	43.6	9	9	260.1	46	42
8.6	7	2	51.3	18	13	305.9	76	59
10.1	11	4	60.3	36	21	359.9	100	80
11.9	11	6	70.9	29	27	423.3	62	94
14.0	6	8	83.5	24	32	497.9	30	100
16.5	0	8	98.2	0	32	585.7	0	100
19.4	0	8	115.5	0	32	689.0	0	100
22.8	0	8	135.8	0	32	810.5	0	100
26.8	0	8	159.8	0	32	953.3	0	100

Intensity

Supplementary Figure 54. Size distribution of 2.5 µM WT-G3 plus 7 µM ASF. **a** Surface area- and **b** intensity-weighted. Data in columns are technical replicates of **a** or **b**.

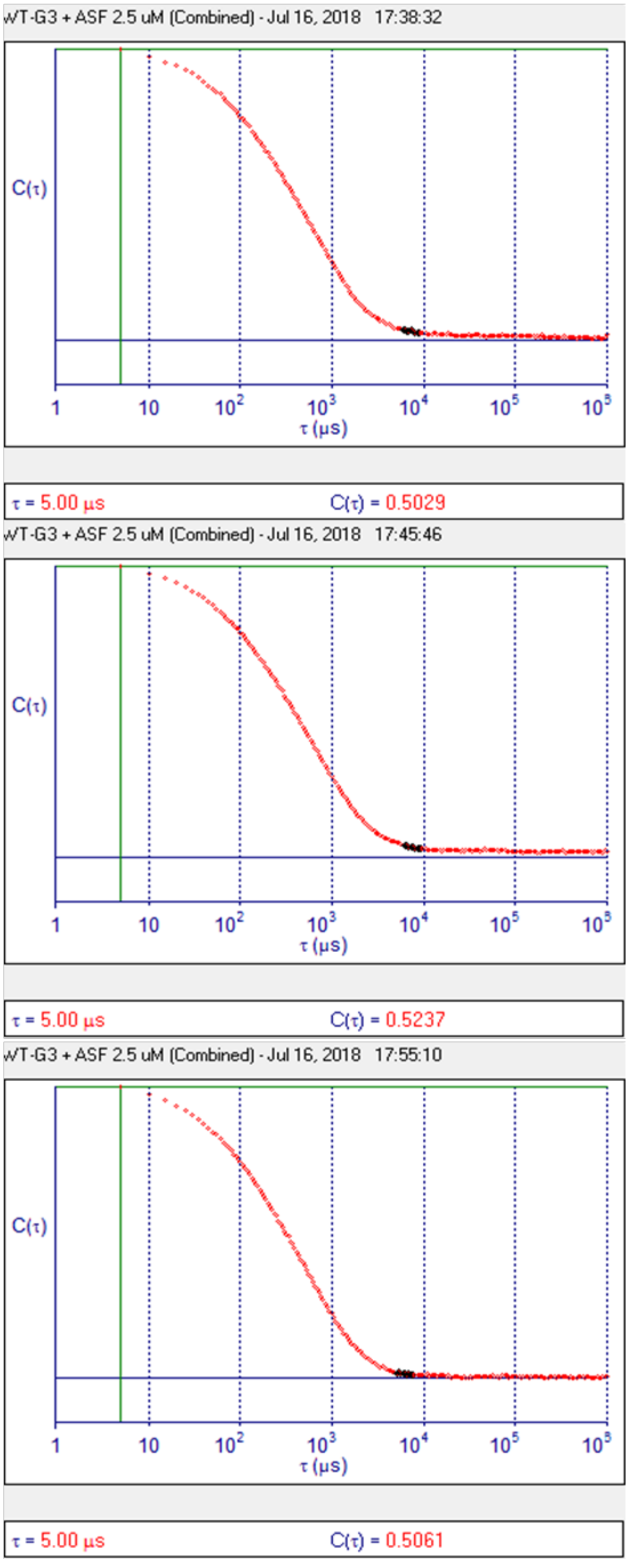

Supplementary Figure 55. Correlation functions for DLS measurements of 2.5 µM WT-G3 plus 7 µM ASF. Data are technical replicates.

a **b**

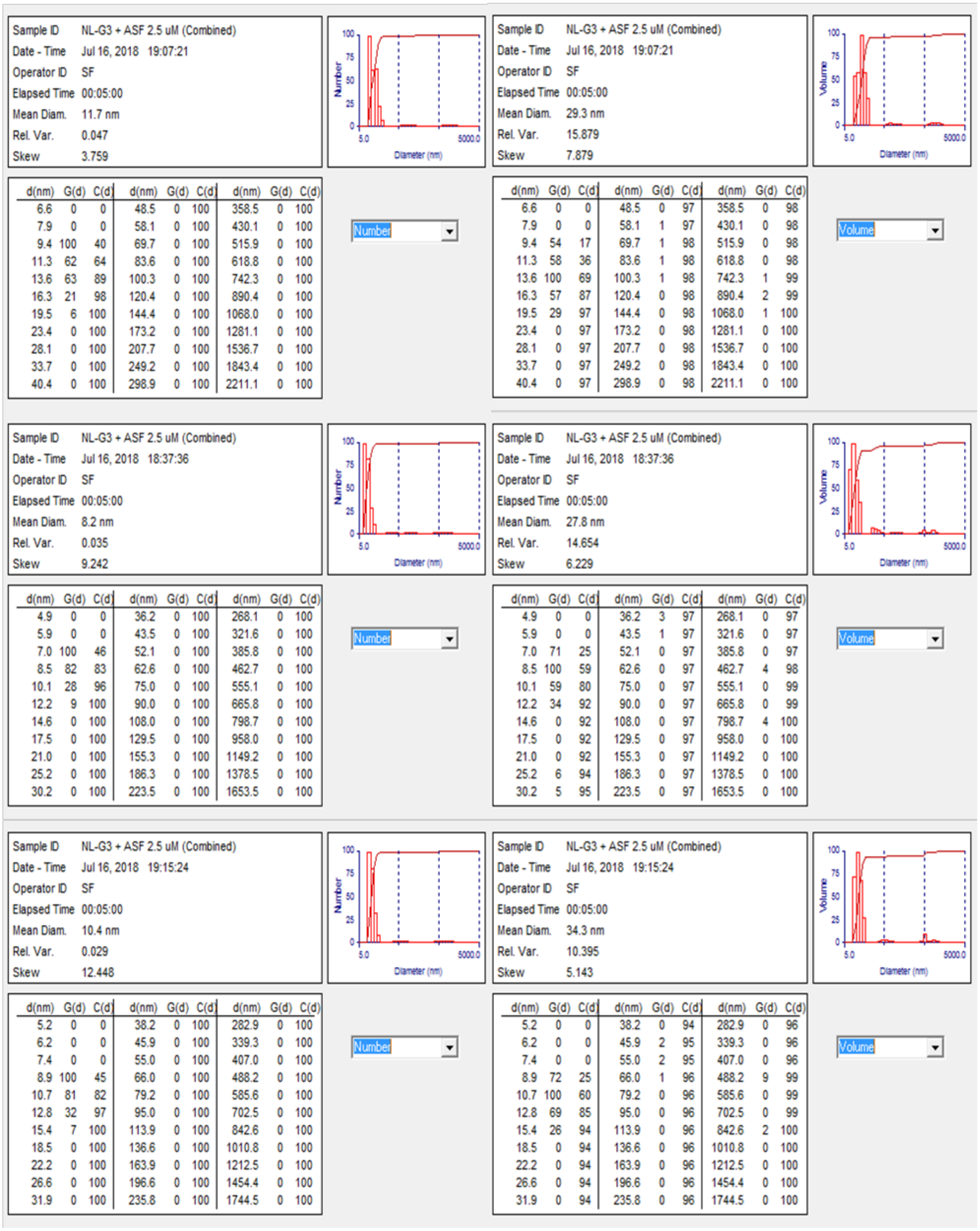

a

Sample ID NL-G3 + ASF 2.5 uM (Combined)
Date - Time Jul 16, 2018 19:07:21
Operator ID SF
Elapsed Time 00:05:00
Mean Diam. 11.7 nm
Rel. Var. 0.047
Skew 3.759

d(nm)	G(d)	C(d)	d(nm)	G(d)	C(d)	d(nm)	G(d)	C(d)
6.6	0	0	48.5	0	100	358.5	0	100
7.9	0	0	58.1	0	100	430.1	0	100
9.4	100	40	69.7	0	100	515.9	0	100
11.3	62	64	83.6	0	100	618.8	0	100
13.6	63	89	100.3	0	100	742.3	0	100
16.3	21	98	120.4	0	100	890.4	0	100
19.5	6	100	144.4	0	100	1068.0	0	100
23.4	0	100	173.2	0	100	1281.1	0	100
28.1	0	100	207.7	0	100	1536.7	0	100
33.7	0	100	249.2	0	100	1843.4	0	100
40.4	0	100	298.9	0	100	2211.1	0	100

Sample ID NL-G3 + ASF 2.5 uM (Combined)
Date - Time Jul 16, 2018 18:37:36
Operator ID SF
Elapsed Time 00:05:00
Mean Diam. 8.2 nm
Rel. Var. 0.035
Skew 9.242

d(nm)	G(d)	C(d)	d(nm)	G(d)	C(d)	d(nm)	G(d)	C(d)
4.9	0	0	36.2	0	100	268.1	0	100
5.9	0	0	43.5	0	100	321.6	0	100
7.0	100	46	52.1	0	100	385.8	0	100
8.5	82	83	62.6	0	100	462.7	0	100
10.1	28	96	75.0	0	100	555.1	0	100
12.2	9	100	90.0	0	100	665.8	0	100
14.6	0	100	108.0	0	100	798.7	0	100
17.5	0	100	129.5	0	100	958.0	0	100
21.0	0	100	155.3	0	100	1149.2	0	100
25.2	0	100	186.3	0	100	1378.5	0	100
30.2	0	100	223.5	0	100	1653.5	0	100

Sample ID NL-G3 + ASF 2.5 uM (Combined)
Date - Time Jul 16, 2018 19:15:24
Operator ID SF
Elapsed Time 00:05:00
Mean Diam. 10.4 nm
Rel. Var. 0.029
Skew 12.448

d(nm)	G(d)	C(d)	d(nm)	G(d)	C(d)	d(nm)	G(d)	C(d)
5.2	0	0	38.2	0	100	282.9	0	100
6.2	0	0	45.9	0	100	339.3	0	100
7.4	0	0	55.0	0	100	407.0	0	100
8.9	100	45	66.0	0	100	488.2	0	100
10.7	81	82	79.2	0	100	585.6	0	100
12.8	32	97	95.0	0	100	702.5	0	100
15.4	7	100	113.9	0	100	842.6	0	100
18.5	0	100	136.6	0	100	1010.8	0	100
22.2	0	100	163.9	0	100	1212.5	0	100
26.6	0	100	196.6	0	100	1454.4	0	100
31.9	0	100	235.8	0	100	1744.5	0	100

b

Sample ID NL-G3 + ASF 2.5 uM (Combined)
Date - Time Jul 16, 2018 19:07:21
Operator ID SF
Elapsed Time 00:05:00
Mean Diam. 29.3 nm
Rel. Var. 15.879
Skew 7.879

d(nm)	G(d)	C(d)	d(nm)	G(d)	C(d)	d(nm)	G(d)	C(d)
6.6	0	0	48.5	0	97	358.5	0	98
7.9	0	0	58.1	1	97	430.1	0	98
9.4	54	17	69.7	1	98	515.9	0	98
11.3	58	36	83.6	1	98	618.8	0	98
13.6	100	69	100.3	1	98	742.3	1	99
16.3	57	87	120.4	0	98	890.4	2	99
19.5	29	97	144.4	0	98	1068.0	1	100
23.4	0	97	173.2	0	98	1281.1	0	100
28.1	0	97	207.7	0	98	1536.7	0	100
33.7	0	97	249.2	0	98	1843.4	0	100
40.4	0	97	298.9	0	98	2211.1	0	100

Sample ID NL-G3 + ASF 2.5 uM (Combined)
Date - Time Jul 16, 2018 18:37:36
Operator ID SF
Elapsed Time 00:05:00
Mean Diam. 27.8 nm
Rel. Var. 14.654
Skew 6.229

d(nm)	G(d)	C(d)	d(nm)	G(d)	C(d)	d(nm)	G(d)	C(d)
4.9	0	0	36.2	3	97	268.1	0	97
5.9	0	0	43.5	1	97	321.6	0	97
7.0	71	25	52.1	0	97	385.8	0	97
8.5	100	59	62.6	0	97	462.7	4	98
10.1	59	80	75.0	0	97	555.1	0	99
12.2	34	92	90.0	0	97	665.8	0	99
14.6	0	92	108.0	0	97	798.7	4	100
17.5	0	92	129.5	0	97	958.0	0	100
21.0	0	92	155.3	0	97	1149.2	0	100
25.2	6	94	186.3	0	97	1378.5	0	100
30.2	5	95	223.5	0	97	1653.5	0	100

Sample ID NL-G3 + ASF 2.5 uM (Combined)
Date - Time Jul 16, 2018 19:15:24
Operator ID SF
Elapsed Time 00:05:00
Mean Diam. 34.3 nm
Rel. Var. 10.395
Skew 5.143

d(nm)	G(d)	C(d)	d(nm)	G(d)	C(d)	d(nm)	G(d)	C(d)
5.2	0	0	38.2	0	94	282.9	0	96
6.2	0	0	45.9	2	95	339.3	0	96
7.4	0	0	55.0	2	95	407.0	0	96
8.9	72	25	66.0	1	96	488.2	9	99
10.7	100	60	79.2	0	96	585.6	0	99
12.8	69	85	95.0	0	96	702.5	0	99
15.4	26	94	113.9	0	96	842.6	2	100
18.5	0	94	136.6	0	96	1010.8	0	100
22.2	0	94	163.9	0	96	1212.5	0	100
26.6	0	94	196.6	0	96	1454.4	0	100
31.9	0	94	235.8	0	96	1744.5	0	100

Supplementary Figure 56. Size distribution of 2.5 μM NL-G3 plus 7 μM ASF. **a** Number- and **b** volume-weighted. Data in columns are technical replicates of **a** or **b**.

a

Sample ID NL-G3 + ASF 2.5 uM (Combined)
Date - Time Jul 16, 2018 19:07:21
Operator ID SF
Elapsed Time 00:05:00
Mean Diam. 13.2 nm
Rel. Var. 1.217
Skew 58.329

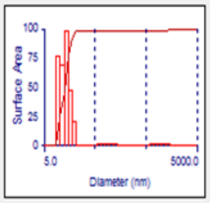

d(nm)	G(d)	C(d)	d(nm)	G(d)	C(d)	d(nm)	G(d)	C(d)
6.6	0	0	48.5	0	100	358.5	0	100
7.9	0	0	58.1	0	100	430.1	0	100
9.4	77	24	69.7	0	100	515.9	0	100
11.3	69	46	83.6	0	100	618.8	0	100
13.6	100	78	100.3	0	100	742.3	0	100
16.3	48	93	120.4	0	100	890.4	0	100
19.5	20	100	144.4	0	100	1068.0	0	100
23.4	0	100	173.2	0	100	1281.1	0	100
28.1	0	100	207.7	0	100	1536.7	0	100
33.7	0	100	249.2	0	100	1843.4	0	100
40.4	0	100	298.9	0	100	2211.1	0	100

Surface Area

Sample ID NL-G3 + ASF 2.5 uM (Combined)
Date - Time Jul 16, 2018 18:37:36
Operator ID SF
Elapsed Time 00:05:00
Mean Diam. 9.3 nm
Rel. Var. 2.005
Skew 47.319

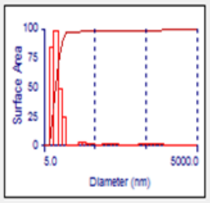

d(nm)	G(d)	C(d)	d(nm)	G(d)	C(d)	d(nm)	G(d)	C(d)
4.9	0	0	36.2	1	100	268.1	0	100
5.9	0	0	43.5	0	100	321.6	0	100
7.0	85	32	52.1	0	100	385.8	0	100
8.5	100	71	62.6	0	100	462.7	0	100
10.1	49	89	75.0	0	100	555.1	0	100
12.2	24	98	90.0	0	100	665.8	0	100
14.6	0	98	108.0	0	100	798.7	0	100
17.5	0	98	129.5	0	100	958.0	0	100
21.0	0	98	155.3	0	100	1149.2	0	100
25.2	2	99	186.3	0	100	1378.5	0	100
30.2	1	100	223.5	0	100	1653.5	0	100

Surface Area

Sample ID NL-G3 + ASF 2.5 uM (Combined)
Date - Time Jul 16, 2018 19:15:24
Operator ID SF
Elapsed Time 00:05:00
Mean Diam. 11.6 nm
Rel. Var. 1.970
Skew 33.851

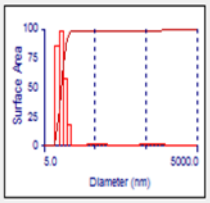

d(nm)	G(d)	C(d)	d(nm)	G(d)	C(d)	d(nm)	G(d)	C(d)
5.2	0	0	38.2	0	100	282.9	0	100
6.2	0	0	45.9	0	100	339.3	0	100
7.4	0	0	55.0	0	100	407.0	0	100
8.9	86	33	66.0	0	100	488.2	0	100
10.7	100	71	79.2	0	100	585.6	0	100
12.8	57	93	95.0	0	100	702.5	0	100
15.4	18	99	113.9	0	100	842.6	0	100
18.5	0	99	136.6	0	100	1010.8	0	100
22.2	0	99	163.9	0	100	1212.5	0	100
26.6	0	99	196.6	0	100	1454.4	0	100
31.9	0	99	235.8	0	100	1744.5	0	100

Surface Area

b

Sample ID NL-G3 + ASF 2.5 uM (Combined)
Date - Time Jul 16, 2018 19:07:21
Operator ID SF
Elapsed Time 00:05:00
Mean Diam. 597.8 nm
Rel. Var. 0.512
Skew -0.194

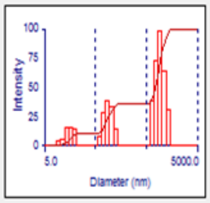

d(nm)	G(d)	C(d)	d(nm)	G(d)	C(d)	d(nm)	G(d)	C(d)
6.6	0	0	48.5	0	11	358.5	0	36
7.9	0	0	58.1	7	12	430.1	0	36
9.4	3	1	69.7	28	18	515.9	0	36
11.3	5	2	83.6	39	26	618.8	38	44
13.6	15	5	100.3	33	33	742.3	73	59
16.3	15	8	120.4	13	36	890.4	100	80
19.5	14	11	144.4	0	36	1068.0	65	94
23.4	0	11	173.2	0	36	1281.1	30	100
28.1	0	11	207.7	0	36	1536.7	0	100
33.7	0	11	249.2	0	36	1843.4	0	100
40.4	0	11	298.9	0	36	2211.1	0	100

Intensity

Sample ID NL-G3 + ASF 2.5 uM (Combined)
Date - Time Jul 16, 2018 18:37:36
Operator ID SF
Elapsed Time 00:05:00
Mean Diam. 427.0 nm
Rel. Var. 0.510
Skew -0.109

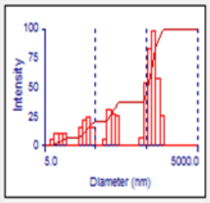

d(nm)	G(d)	C(d)	d(nm)	G(d)	C(d)	d(nm)	G(d)	C(d)
4.9	0	0	36.2	24	18	268.1	0	37
5.9	0	0	43.5	15	21	321.6	0	37
7.0	4	1	52.1	0	21	385.8	5	38
8.5	10	3	62.6	0	21	462.7	54	49
10.1	10	5	75.0	5	22	555.1	84	65
12.2	10	6	90.0	30	27	665.8	100	84
14.6	0	6	108.0	26	32	798.7	57	95
17.5	0	6	129.5	25	37	958.0	25	100
21.0	0	6	155.3	0	37	1149.2	0	100
25.2	14	9	186.3	0	37	1378.5	0	100
30.2	21	13	223.5	0	37	1653.5	0	100

Intensity

Sample ID NL-G3 + ASF 2.5 uM (Combined)
Date - Time Jul 16, 2018 19:15:24
Operator ID SF
Elapsed Time 00:05:00
Mean Diam. 436.2 nm
Rel. Var. 0.450
Skew -0.247

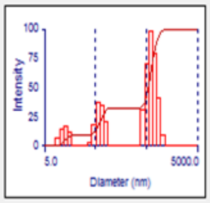

d(nm)	G(d)	C(d)	d(nm)	G(d)	C(d)	d(nm)	G(d)	C(d)
5.2	0	0	38.2	2	10	282.9	0	32
6.2	0	0	45.9	18	13	339.3	0	32
7.4	0	0	55.0	37	21	407.0	30	38
8.9	6	1	66.0	35	28	488.2	71	53
10.7	13	4	79.2	20	32	585.6	100	73
12.8	16	7	95.0	0	32	702.5	80	90
15.4	10	9	113.9	0	32	842.6	41	98
18.5	0	9	136.6	0	32	1010.8	9	100
22.2	0	9	163.9	0	32	1212.5	0	100
26.6	0	9	196.6	0	32	1454.4	0	100
31.9	0	9	235.8	0	32	1744.5	0	100

Intensity

Supplementary Figure 57. Size distribution of 2.5 μM NL-G3 plus 7 μM ASF. **a** Surface area- and **b** intensity-weighted. Data in columns are technical replicates of **a** or **b**.

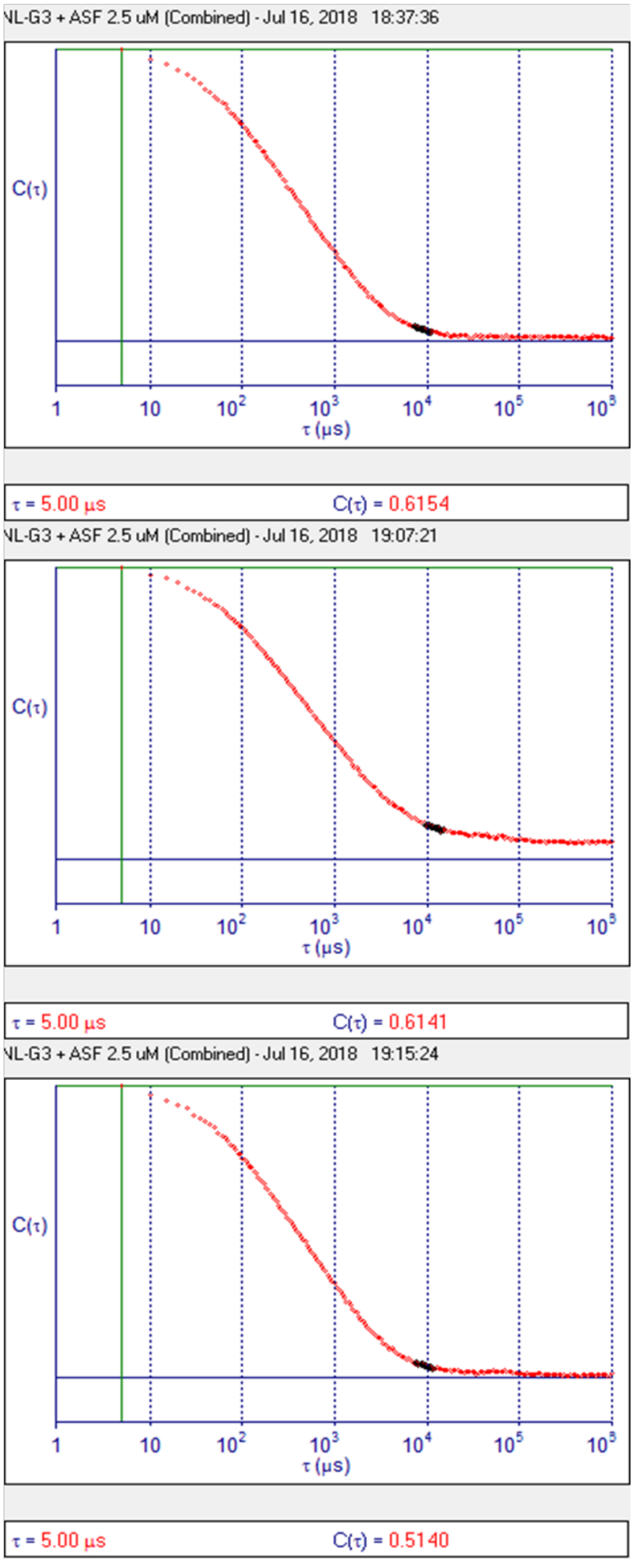

Supplementary Figure 58. Correlation functions for DLS measurements of 2.5 μM NL-G3 plus 7 μM ASF. Data are technical replicates.

a

Sample ID NL-TT-G3 + ASF 2.5 uM (Combined)
Date - Time Jul 16, 2018 18:08:54
Operator ID SF
Elapsed Time 00:05:00
Mean Diam. 16.9 nm
Rel. Var. 0.038
Skew 26.059

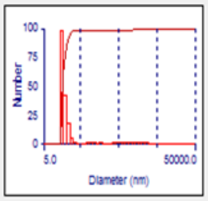

d(nm)	G(d)	C(d)	d(nm)	G(d)	C(d)	d(nm)	G(d)	C(d)
6.8	0	0	59.0	0	100	510.6	0	100
8.3	0	0	71.8	0	100	621.3	0	100
10.1	0	0	87.3	0	100	756.0	0	100
12.3	0	0	106.3	0	100	919.9	0	100
14.9	100	61	129.3	0	100	1119.3	0	100
18.2	43	87	157.3	0	100	1361.9	0	100
22.1	17	97	191.4	0	100	1657.2	0	100
26.9	4	100	232.9	0	100	2016.4	0	100
32.7	1	100	283.4	0	100	2453.6	0	100
39.8	0	100	344.9	0	100	2985.5	0	100
48.5	0	100	419.6	0	100	3632.8	0	100

Number

Sample ID NL-TT-G3 + ASF 2.5 uM (Combined)
Date - Time Jul 16, 2018 18:21:50
Operator ID SF
Elapsed Time 00:05:00
Mean Diam. 17.4 nm
Rel. Var. 0.031
Skew 38.184

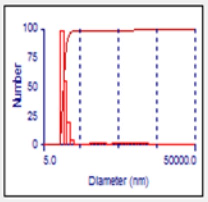

d(nm)	G(d)	C(d)	d(nm)	G(d)	C(d)	d(nm)	G(d)	C(d)
7.0	0	0	60.7	0	100	525.4	0	100
8.5	0	0	73.8	0	100	639.3	0	100
10.4	0	0	89.8	0	100	777.8	0	100
12.6	0	0	109.3	0	100	946.5	0	100
15.4	100	56	133.0	0	100	1151.7	0	100
18.7	55	88	161.9	0	100	1401.3	0	100
22.8	19	98	197.0	0	100	1705.1	0	100
27.7	3	100	239.7	0	100	2074.8	0	100
33.7	0	100	291.6	0	100	2524.6	0	100
41.0	0	100	354.8	0	100	3071.9	0	100
49.9	0	100	431.8	0	100	3737.9	0	100

Number

Sample ID NL-TT-G3 + ASF 2.5 uM (Combined)
Date - Time Jul 16, 2018 18:29:55
Operator ID SF
Elapsed Time 00:05:00
Mean Diam. 15.6 nm
Rel. Var. 0.022
Skew 25.574

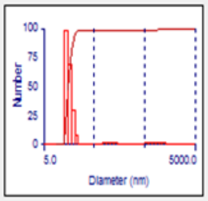

d(nm)	G(d)	C(d)	d(nm)	G(d)	C(d)	d(nm)	G(d)	C(d)
10.0	0	0	57.5	0	100	331.3	0	100
11.7	0	0	67.5	0	100	388.5	0	100
13.7	100	49	79.1	0	100	455.5	0	100
16.1	69	82	92.8	0	100	534.0	0	100
18.9	30	97	108.8	0	100	626.2	0	100
22.1	7	100	127.5	0	100	734.2	0	100
26.0	0	100	149.5	0	100	860.8	0	100
30.4	0	100	175.3	0	100	1009.3	0	100
35.7	0	100	205.5	0	100	1183.5	0	100
41.9	0	100	241.0	0	100	1387.6	0	100
49.1	0	100	282.6	0	100	1627.0	0	100

Number

b

Sample ID NL-TT-G3 + ASF 2.5 uM (Combined)
Date - Time Jul 16, 2018 18:08:54
Operator ID SF
Elapsed Time 00:05:00
Mean Diam. 130.0 nm
Rel. Var. 5.597
Skew 3.482

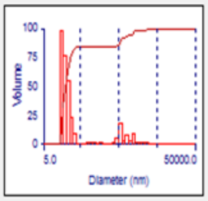

d(nm)	G(d)	C(d)	d(nm)	G(d)	C(d)	d(nm)	G(d)	C(d)
6.8	0	0	59.0	0	85	510.6	18	93
8.3	0	0	71.8	1	85	621.3	2	93
10.1	0	0	87.3	1	85	756.0	7	96
12.3	0	0	106.3	0	85	919.9	2	96
14.9	100	32	129.3	0	85	1119.3	8	99
18.2	77	57	157.3	0	85	1361.9	1	99
22.1	55	75	191.4	0	85	1657.2	1	100
26.9	23	82	232.9	0	85	2016.4	1	100
32.7	8	85	283.4	0	85	2453.6	1	100
39.8	0	85	344.9	0	85	2985.5	0	100
48.5	0	85	419.6	4	87	3632.8	0	100

Volume

Sample ID NL-TT-G3 + ASF 2.5 uM (Combined)
Date - Time Jul 16, 2018 18:21:50
Operator ID SF
Elapsed Time 00:05:00
Mean Diam. 166.0 nm
Rel. Var. 4.807
Skew 2.661

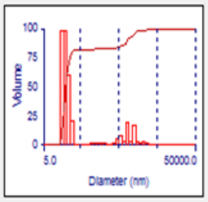

d(nm)	G(d)	C(d)	d(nm)	G(d)	C(d)	d(nm)	G(d)	C(d)
7.0	0	0	60.7	0	83	525.4	7	87
8.5	0	0	73.8	0	83	639.3	2	88
10.4	0	0	89.8	0	83	777.8	18	93
12.6	0	0	109.3	1	83	946.5	2	94
15.4	100	30	133.0	0	84	1151.7	17	99
18.7	99	59	161.9	0	84	1401.3	2	99
22.8	60	77	197.0	0	84	1705.1	1	99
27.7	20	83	239.7	0	84	2074.8	1	100
33.7	0	83	291.6	0	84	2524.6	0	100
41.0	0	83	354.8	0	84	3071.9	0	100
49.9	0	83	431.8	4	85	3737.9	0	100

Volume

Sample ID NL-TT-G3 + ASF 2.5 uM (Combined)
Date - Time Jul 16, 2018 18:29:55
Operator ID SF
Elapsed Time 00:05:00
Mean Diam. 71.3 nm
Rel. Var. 7.778
Skew 3.537

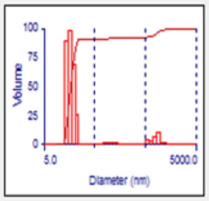

d(nm)	G(d)	C(d)	d(nm)	G(d)	C(d)	d(nm)	G(d)	C(d)
10.0	0	0	57.5	0	92	331.3	0	93
11.7	0	0	67.5	0	92	388.5	0	93
13.7	90	29	79.1	1	92	455.5	0	93
16.1	100	61	92.8	1	93	534.0	3	94
18.9	70	84	108.8	1	93	626.2	2	94
22.1	26	92	127.5	0	93	734.2	6	96
26.0	0	92	149.5	0	93	860.8	9	99
30.4	0	92	175.3	0	93	1009.3	1	100
35.7	0	92	205.5	0	93	1183.5	1	100
41.9	0	92	241.0	0	93	1387.6	0	100
49.1	0	92	282.6	0	93	1627.0	0	100

Volume

Supplementary Figure 59. Size distribution of 2.5 μM NL-TT-G3 plus 7 μM ASF. **a** Number- and **b** volume-weighted. Data in columns are technical replicates of **a** or **b**.

a

Sample ID NL-TT-G3 + ASF 2.5 uM (Combined)
Date - Time Jul 16, 2018 18:08:54
Operator ID SF
Elapsed Time 00:05:00
Mean Diam. 21.3 nm
Rel. Var. 5.115
Skew 19.912

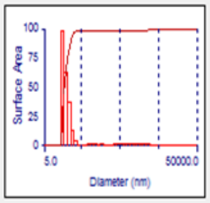

d(nm)	G(d)	C(d)	d(nm)	G(d)	C(d)	d(nm)	G(d)	C(d)
6.8	0	0	59.0	0	99	510.6	1	100
8.3	0	0	71.8	0	99	621.3	0	100
10.1	0	0	87.3	0	99	756.0	0	100
12.3	0	0	106.3	0	100	919.9	0	100
14.9	100	46	129.3	0	100	1119.3	0	100
18.2	63	75	157.3	0	100	1361.9	0	100
22.1	37	92	191.4	0	100	1657.2	0	100
26.9	13	98	232.9	0	100	2016.4	0	100
32.7	4	99	283.4	0	100	2453.6	0	100
39.8	0	99	344.9	0	100	2985.5	0	100
48.5	0	99	419.6	0	100	3632.8	0	100

Surface Area

Sample ID NL-TT-G3 + ASF 2.5 uM (Combined)
Date - Time Jul 16, 2018 18:21:50
Operator ID SF
Elapsed Time 00:05:00
Mean Diam. 22.0 nm
Rel. Var. 6.550
Skew 18.516

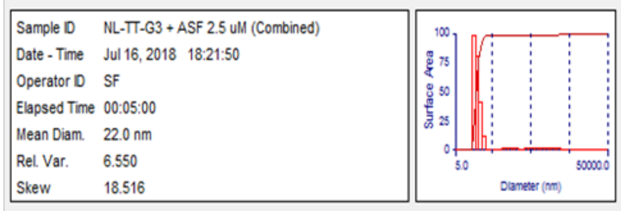

d(nm)	G(d)	C(d)	d(nm)	G(d)	C(d)	d(nm)	G(d)	C(d)
7.0	0	0	60.7	0	99	525.4	0	100
8.5	0	0	73.8	0	99	639.3	0	100
10.4	0	0	89.8	0	99	777.8	0	100
12.6	0	0	109.3	0	100	946.5	0	100
15.4	100	43	133.0	0	100	1151.7	0	100
18.7	82	77	161.9	0	100	1401.3	0	100
22.8	41	95	197.0	0	100	1705.1	0	100
27.7	11	99	239.7	0	100	2074.8	0	100
33.7	0	99	291.6	0	100	2524.6	0	100
41.0	0	99	354.8	0	100	3071.9	0	100
49.9	0	99	431.8	0	100	3737.9	0	100

Surface Area

Sample ID NL-TT-G3 + ASF 2.5 uM (Combined)
Date - Time Jul 16, 2018 18:29:55
Operator ID SF
Elapsed Time 00:05:00
Mean Diam. 17.6 nm
Rel. Var. 3.061
Skew 25.130

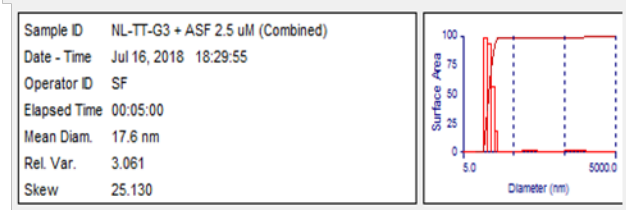

d(nm)	G(d)	C(d)	d(nm)	G(d)	C(d)	d(nm)	G(d)	C(d)
10.0	0	0	57.5	0	100	331.3	0	100
11.7	0	0	67.5	0	100	388.5	0	100
13.7	100	37	79.1	0	100	455.5	0	100
16.1	95	72	92.8	0	100	534.0	0	100
18.9	56	93	108.8	0	100	626.2	0	100
22.1	18	100	127.5	0	100	734.2	0	100
26.0	0	100	149.5	0	100	860.8	0	100
30.4	0	100	175.3	0	100	1009.3	0	100
35.7	0	100	205.5	0	100	1183.5	0	100
41.9	0	100	241.0	0	100	1387.6	0	100
49.1	0	100	282.6	0	100	1627.0	0	100

Surface Area

b

Sample ID NL-TT-G3 + ASF 2.5 uM (Combined)
Date - Time Jul 16, 2018 18:08:54
Operator ID SF
Elapsed Time 00:05:00
Mean Diam. 698.8 nm
Rel. Var. 0.429
Skew 1.288

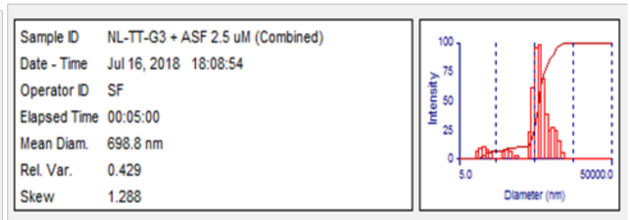

d(nm)	G(d)	C(d)	d(nm)	G(d)	C(d)	d(nm)	G(d)	C(d)
6.8	0	0	59.0	0	7	510.6	97	44
8.3	0	0	71.8	6	8	621.3	100	63
10.1	0	0	87.3	7	9	756.0	70	76
12.3	0	0	106.3	6	10	919.9	39	83
14.9	6	1	129.3	0	10	1119.3	24	87
18.2	8	3	157.3	2	11	1361.9	26	92
22.1	10	4	191.4	0	11	1657.2	24	97
26.9	8	6	232.9	0	11	2016.4	14	99
32.7	5	7	283.4	0	11	2453.6	4	100
39.8	0	7	344.9	23	15	2985.5	0	100
48.5	0	7	419.6	62	26	3632.8	0	100

Intensity

Sample ID NL-TT-G3 + ASF 2.5 uM (Combined)
Date - Time Jul 16, 2018 18:21:50
Operator ID SF
Elapsed Time 00:05:00
Mean Diam. 731.8 nm
Rel. Var. 0.440
Skew 1.210

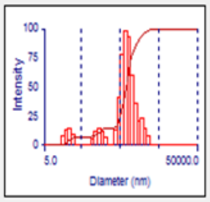

d(nm)	G(d)	C(d)	d(nm)	G(d)	C(d)	d(nm)	G(d)	C(d)
7.0	0	0	60.7	0	7	525.4	79	38
8.5	0	0	73.8	0	7	639.3	100	56
10.4	0	0	89.8	4	8	777.8	94	72
12.6	0	0	109.3	11	10	946.5	60	83
15.4	7	1	133.0	14	12	1151.7	35	89
18.7	13	3	161.9	12	14	1401.3	20	92
22.8	14	6	197.0	5	15	1705.1	22	96
27.7	8	7	239.7	0	15	2074.8	14	99
33.7	0	7	291.6	0	15	2524.6	7	100
41.0	0	7	354.8	13	17	3071.9	0	100
49.9	0	7	431.8	41	25	3737.9	0	100

Intensity

Sample ID NL-TT-G3 + ASF 2.5 uM (Combined)
Date - Time Jul 16, 2018 18:29:55
Operator ID SF
Elapsed Time 00:05:00
Mean Diam. 638.0 nm
Rel. Var. 0.192
Skew -0.998

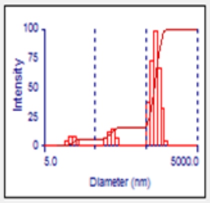

d(nm)	G(d)	C(d)	d(nm)	G(d)	C(d)	d(nm)	G(d)	C(d)
10.0	0	0	57.5	0	6	331.3	0	16
11.7	0	0	67.5	0	6	388.5	0	16
13.7	4	1	79.1	6	8	455.5	0	16
16.1	7	3	92.8	11	11	534.0	37	26
18.9	8	5	108.8	13	14	626.2	73	45
22.1	5	6	127.5	6	16	734.2	100	72
26.0	0	6	149.5	0	16	860.8	67	91
30.4	0	6	175.3	0	16	1009.3	32	99
35.7	0	6	205.5	0	16	1183.5	3	100
41.9	0	6	241.0	0	16	1387.6	0	100
49.1	0	6	282.6	0	16	1627.0	0	100

Intensity

Supplementary Figure 60. Size distribution of 2.5 μM NL-TT-G3 plus 7 μM ASF. **a** Surface area- and **b** intensity-weighted. Data in columns are technical replicates of **a** or **b**.

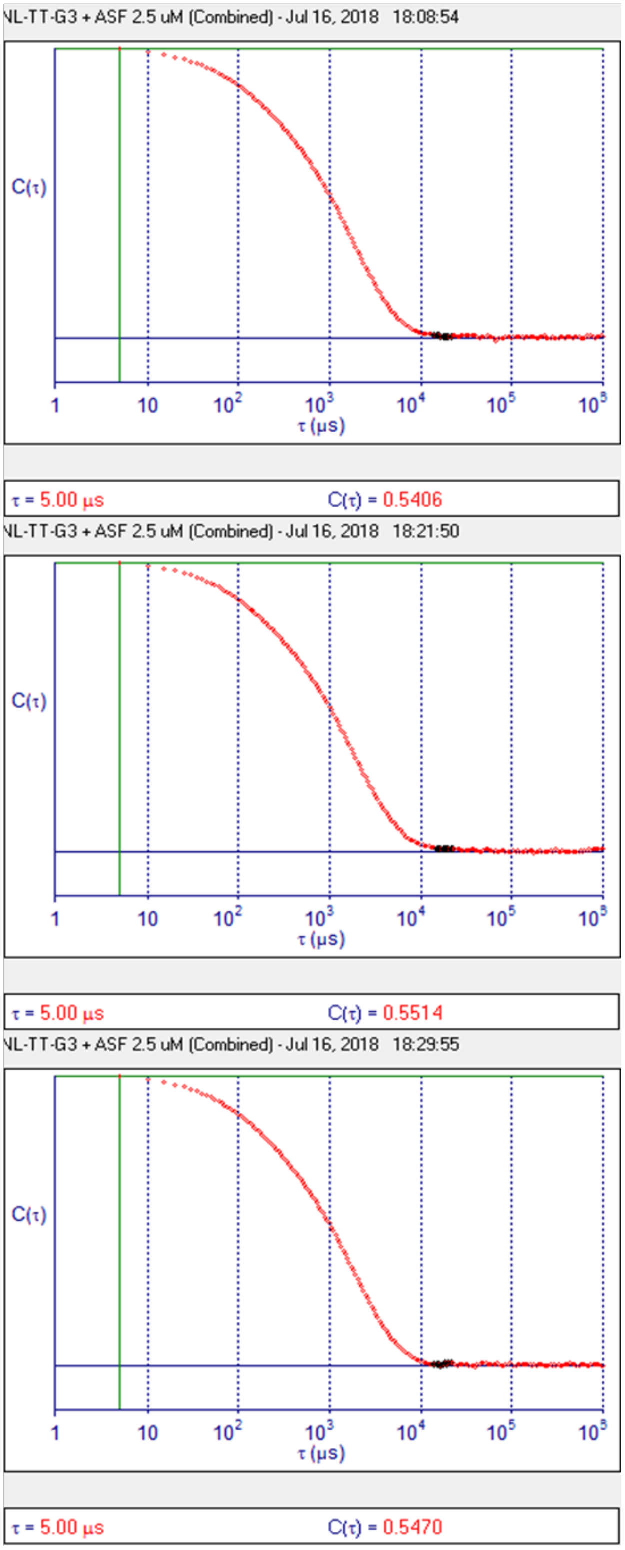

Supplementary Figure 61. Correlation functions for DLS measurements of 2.5 µM NL-TT-G3 plus 7 µM ASF. Data are technical replicates.

a

Sample ID	WT-G3 + ASF (Combined)
Date - Time	Jul 16, 2018 14:55:20
Operator ID	SF
Elapsed Time	00:05:00
Mean Diam.	869.9 nm
Rel. Var.	0.137
Skew	5.462

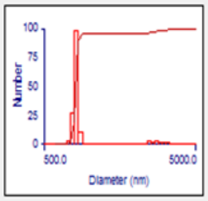

d(nm)	G(d)	C(d)	d(nm)	G(d)	C(d)	d(nm)	G(d)	C(d)
615.5	0	0	1155.6	0	97	2169.8	0	97
651.8	0	0	1223.7	0	97	2297.7	0	97
690.2	0	0	1295.9	0	97	2433.1	1	98
730.9	2	1	1372.2	0	97	2576.5	1	98
773.9	27	20	1453.1	0	97	2728.4	2	100
819.6	100	90	1538.8	0	97	2889.2	0	100
867.9	10	97	1629.5	0	97	3059.5	0	100
919.0	0	97	1725.5	0	97	3239.8	0	100
973.2	0	97	1827.2	0	97	3430.8	0	100
1030.5	0	97	1934.9	0	97	3633.0	0	100
1091.3	0	97	2049.0	0	97	3847.1	0	100

Number

Sample ID	WT-G3 + ASF (Combined)
Date - Time	Jul 16, 2018 15:06:54
Operator ID	SF
Elapsed Time	00:05:00
Mean Diam.	1225.1 nm
Rel. Var.	0.078
Skew	7.186

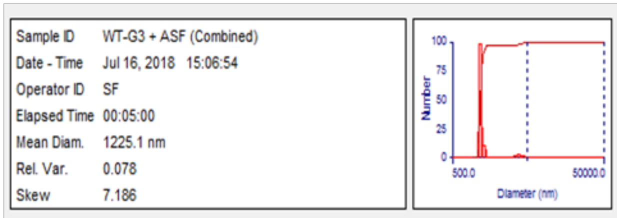

d(nm)	G(d)	C(d)	d(nm)	G(d)	C(d)	d(nm)	G(d)	C(d)
943.1	0	0	1681.3	0	98	2997.5	0	98
994.0	0	0	1772.1	0	98	3159.3	0	98
1047.6	0	0	1867.7	0	98	3329.8	0	98
1104.2	22	13	1968.5	0	98	3509.5	1	99
1163.8	100	74	2074.7	0	98	3698.9	1	99
1226.6	25	89	2186.7	0	98	3898.5	0	100
1292.8	10	95	2304.7	0	98	4108.9	0	100
1362.5	6	98	2429.1	0	98	4330.6	0	100
1436.1	0	98	2560.2	0	98	4564.4	0	100
1513.6	0	98	2698.4	0	98	4810.7	0	100
1595.2	0	98	2844.0	0	98	5070.3	0	100

Number

Sample ID	WT-G3 + ASF (Combined)
Date - Time	Jul 16, 2018 15:20:40
Operator ID	SF
Elapsed Time	00:05:00
Mean Diam.	2278.1 nm
Rel. Var.	0.006
Skew	5.674

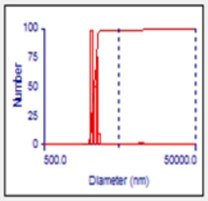

d(nm)	G(d)	C(d)	d(nm)	G(d)	C(d)	d(nm)	G(d)	C(d)
1677.3	0	0	3098.5	0	100	5723.9	0	100
1773.6	0	0	3276.3	0	100	6052.4	0	100
1875.3	0	0	3464.3	0	100	6399.6	0	100
1982.9	3	1	3663.1	0	100	6766.8	0	100
2096.7	100	37	3873.3	0	100	7155.1	0	100
2217.0	43	52	4095.5	0	100	7565.7	0	100
2344.2	50	70	4330.5	0	100	7999.8	0	100
2478.8	76	97	4579.0	0	100	8458.8	0	100
2621.0	8	100	4841.7	0	100	8944.1	0	100
2771.4	0	100	5119.6	0	100	9457.3	0	100
2930.4	0	100	5413.3	0	100	10000.0	0	100

Number

b

Sample ID	WT-G3 + ASF (Combined)
Date - Time	Jul 16, 2018 14:55:20
Operator ID	SF
Elapsed Time	00:05:00
Mean Diam.	1812.1 nm
Rel. Var.	0.275
Skew	-0.051

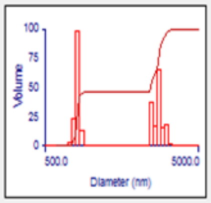

d(nm)	G(d)	C(d)	d(nm)	G(d)	C(d)	d(nm)	G(d)	C(d)
615.5	0	0	1155.6	0	47	2169.8	0	47
651.8	0	0	1223.7	0	47	2297.7	0	47
690.2	0	0	1295.9	0	47	2433.1	37	60
730.9	1	1	1372.2	0	47	2576.5	17	66
773.9	22	8	1453.1	0	47	2728.4	66	88
819.6	100	43	1538.8	0	47	2889.2	15	94
867.9	12	47	1629.5	0	47	3059.5	18	100
919.0	0	47	1725.5	0	47	3239.8	0	100
973.2	0	47	1827.2	0	47	3430.8	0	100
1030.5	0	47	1934.9	0	47	3633.0	0	100
1091.3	0	47	2049.0	0	47	3847.1	0	100

Volume

Sample ID	WT-G3 + ASF (Combined)
Date - Time	Jul 16, 2018 15:06:54
Operator ID	SF
Elapsed Time	00:05:00
Mean Diam.	2134.0 nm
Rel. Var.	0.350
Skew	0.608

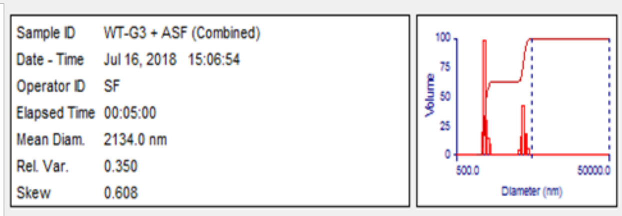

d(nm)	G(d)	C(d)	d(nm)	G(d)	C(d)	d(nm)	G(d)	C(d)
943.1	0	0	1681.3	0	64	2997.5	0	64
994.0	0	0	1772.1	0	64	3159.3	0	64
1047.6	0	0	1867.7	0	64	3329.8	3	65
1104.2	18	7	1968.5	0	64	3509.5	14	70
1163.8	100	44	2074.7	0	64	3698.9	42	86
1226.6	29	55	2186.7	0	64	3898.5	15	92
1292.8	14	60	2304.7	0	64	4108.9	18	98
1362.5	9	64	2429.1	0	64	4330.6	5	100
1436.1	0	64	2560.2	0	64	4564.4	0	100
1513.6	0	64	2698.4	0	64	4810.7	0	100
1595.2	0	64	2844.0	0	64	5070.3	0	100

Volume

Sample ID	WT-G3 + ASF (Combined)
Date - Time	Jul 16, 2018 15:20:40
Operator ID	SF
Elapsed Time	00:05:00
Mean Diam.	2359.4 nm
Rel. Var.	0.062
Skew	11.028

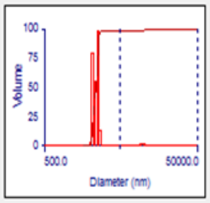

d(nm)	G(d)	C(d)	d(nm)	G(d)	C(d)	d(nm)	G(d)	C(d)
1677.3	0	0	3098.5	0	99	5723.9	0	99
1773.6	0	0	3276.3	0	99	6052.4	0	99
1875.3	0	0	3464.3	0	99	6399.6	0	99
1982.9	2	1	3663.1	0	99	6766.8	0	99
2096.7	80	28	3873.3	0	99	7155.1	0	99
2217.0	41	42	4095.5	0	99	7565.7	0	99
2344.2	55	61	4330.5	0	99	7999.8	0	99
2478.8	100	95	4579.0	0	99	8458.8	0	99
2621.0	13	99	4841.7	0	99	8944.1	1	100
2771.4	0	99	5119.6	0	99	9457.3	1	100
2930.4	0	99	5413.3	0	99	10000.0	1	100

Volume

Supplementary Figure 62. Size distribution of 10 µM WT-G3 plus 7 µM ASF. **a** Number- and **b** volume-weighted. Data in columns are technical replicates of **a** or **b**.

a

Sample ID	WT-G3 + ASF (Combined)
Date - Time	Jul 16, 2018 14:55:20
Operator ID	SF
Elapsed Time	00:05:00
Mean Diam.	1290.6 nm
Rel. Var.	0.404
Skew	1.173

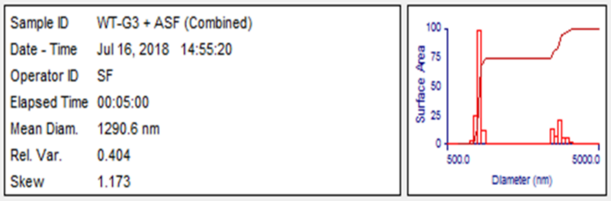

d(nm)	G(d)	C(d)	d(nm)	G(d)	C(d)	d(nm)	G(d)	C(d)
615.5	0	0	1155.6	0	75	2169.8	0	75
651.8	0	0	1223.7	0	75	2297.7	0	75
690.2	0	0	1295.9	0	75	2433.1	12	81
730.9	2	1	1372.2	0	75	2576.5	5	84
773.9	24	14	1453.1	0	75	2728.4	20	95
819.6	100	68	1538.8	0	75	2889.2	4	97
867.9	11	75	1629.5	0	75	3059.5	5	100
919.0	0	75	1725.5	0	75	3239.8	0	100
973.2	0	75	1827.2	0	75	3430.8	0	100
1030.5	0	75	1934.9	0	75	3633.0	0	100
1091.3	0	75	2049.0	0	75	3847.1	0	100

Surface Area

Sample ID	WT-G3 + ASF (Combined)
Date - Time	Jul 16, 2018 15:06:54
Operator ID	SF
Elapsed Time	00:05:00
Mean Diam.	1579.0 nm
Rel. Var.	0.352
Skew	1.971

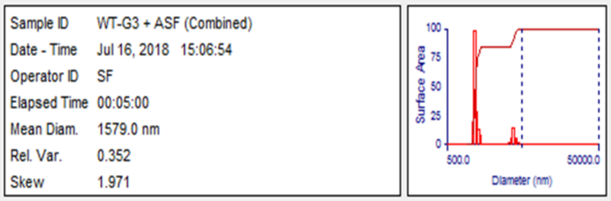

d(nm)	G(d)	C(d)	d(nm)	G(d)	C(d)	d(nm)	G(d)	C(d)
943.1	0	0	1681.3	0	85	2997.5	0	85
994.0	0	0	1772.1	0	85	3159.3	0	85
1047.6	0	0	1867.7	0	85	3329.8	1	85
1104.2	19	10	1968.5	0	85	3509.5	5	88
1163.8	100	61	2074.7	0	85	3698.9	13	95
1226.6	28	75	2186.7	0	85	3898.5	4	97
1292.8	13	81	2304.7	0	85	4108.9	5	99
1362.5	8	85	2429.1	0	85	4330.6	1	100
1436.1	0	85	2560.2	0	85	4564.4	0	100
1513.6	0	85	2698.4	0	85	4810.7	0	100
1595.2	0	85	2844.0	0	85	5070.3	0	100

Surface Area

Sample ID	WT-G3 + ASF (Combined)
Date - Time	Jul 16, 2018 15:20:40
Operator ID	SF
Elapsed Time	00:05:00
Mean Diam.	2313.4 nm
Rel. Var.	0.020
Skew	15.903

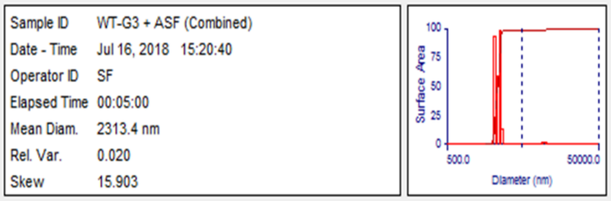

d(nm)	G(d)	C(d)	d(nm)	G(d)	C(d)	d(nm)	G(d)	C(d)
1677.3	0	0	3098.5	0	100	5723.9	0	100
1773.6	0	0	3276.3	0	100	6052.4	0	100
1875.3	0	0	3464.3	0	100	6399.6	0	100
1982.9	2	1	3663.1	0	100	6766.8	0	100
2096.7	94	31	3873.3	0	100	7155.1	0	100
2217.0	46	45	4095.5	0	100	7565.7	0	100
2344.2	59	64	4330.5	0	100	7999.8	0	100
2478.8	100	96	4579.0	0	100	8458.8	0	100
2621.0	12	100	4841.7	0	100	8944.1	0	100
2771.4	0	100	5119.6	0	100	9457.3	0	100
2930.4	0	100	5413.3	0	100	10000.0	0	100

Surface Area

b

Sample ID	WT-G3 + ASF (Combined)
Date - Time	Jul 16, 2018 14:55:20
Operator ID	SF
Elapsed Time	00:05:00
Mean Diam.	2427.4 nm
Rel. Var.	0.089
Skew	-1.640

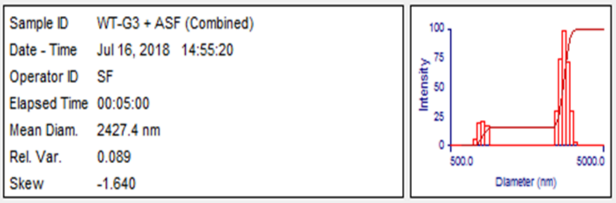

d(nm)	G(d)	C(d)	d(nm)	G(d)	C(d)	d(nm)	G(d)	C(d)
615.5	0	0	1155.6	0	16	2169.8	0	16
651.8	0	0	1223.7	0	16	2297.7	0	16
690.2	0	0	1295.9	0	16	2433.1	30	24
730.9	4	1	1372.2	0	16	2576.5	75	44
773.9	18	6	1453.1	0	16	2728.4	100	72
819.6	20	11	1538.8	0	16	2889.2	72	91
867.9	17	16	1629.5	0	16	3059.5	30	99
919.0	0	16	1725.5	0	16	3239.8	2	100
973.2	0	16	1827.2	0	16	3430.8	0	100
1030.5	0	16	1934.9	0	16	3633.0	0	100
1091.3	0	16	2049.0	0	16	3847.1	0	100

Intensity

Sample ID	WT-G3 + ASF (Combined)
Date - Time	Jul 16, 2018 15:06:54
Operator ID	SF
Elapsed Time	00:05:00
Mean Diam.	3018.2 nm
Rel. Var.	0.163
Skew	-0.719

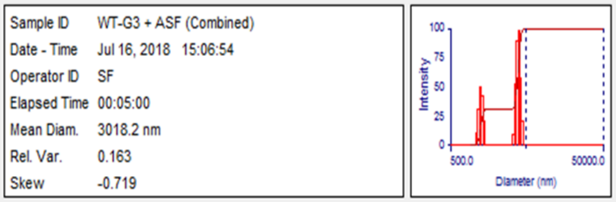

d(nm)	G(d)	C(d)	d(nm)	G(d)	C(d)	d(nm)	G(d)	C(d)
943.1	0	0	1681.3	0	31	2997.5	0	31
994.0	0	0	1772.1	0	31	3159.3	0	31
1047.6	0	0	1867.7	0	31	3329.8	9	33
1104.2	10	2	1968.5	0	31	3509.5	53	44
1163.8	30	8	2074.7	0	31	3698.9	91	63
1226.6	50	19	2186.7	0	31	3898.5	100	84
1292.8	42	27	2304.7	0	31	4108.9	58	96
1362.5	20	31	2429.1	0	31	4330.6	20	100
1436.1	0	31	2560.2	0	31	4564.4	0	100
1513.6	0	31	2698.4	0	31	4810.7	0	100
1595.2	0	31	2844.0	0	31	5070.3	0	100

Intensity

Sample ID	WT-G3 + ASF (Combined)
Date - Time	Jul 16, 2018 15:20:40
Operator ID	SF
Elapsed Time	00:05:00
Mean Diam.	3085.4 nm
Rel. Var.	0.502
Skew	2.589

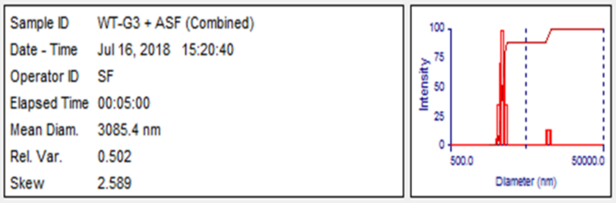

d(nm)	G(d)	C(d)	d(nm)	G(d)	C(d)	d(nm)	G(d)	C(d)
1677.3	0	0	3098.5	0	90	5723.9	0	90
1773.6	0	0	3276.3	0	90	6052.4	0	90
1875.3	0	0	3464.3	0	90	6399.6	0	90
1982.9	4	1	3663.1	0	90	6766.8	0	90
2096.7	35	11	3873.3	0	90	7155.1	0	90
2217.0	71	31	4095.5	0	90	7565.7	0	90
2344.2	100	60	4330.5	0	90	7999.8	0	90
2478.8	70	80	4579.0	0	90	8458.8	0	90
2621.0	34	90	4841.7	0	90	8944.1	12	93
2771.4	0	90	5119.6	0	90	9457.3	12	97
2930.4	0	90	5413.3	0	90	10000.0	12	100

Intensity

Supplementary Figure 63. Size distribution of 10 µM WT-G3 plus 7 µM ASF. **a** Surface area- and **b** intensity-weighted. Data in columns are technical replicates of **a** or **b**.

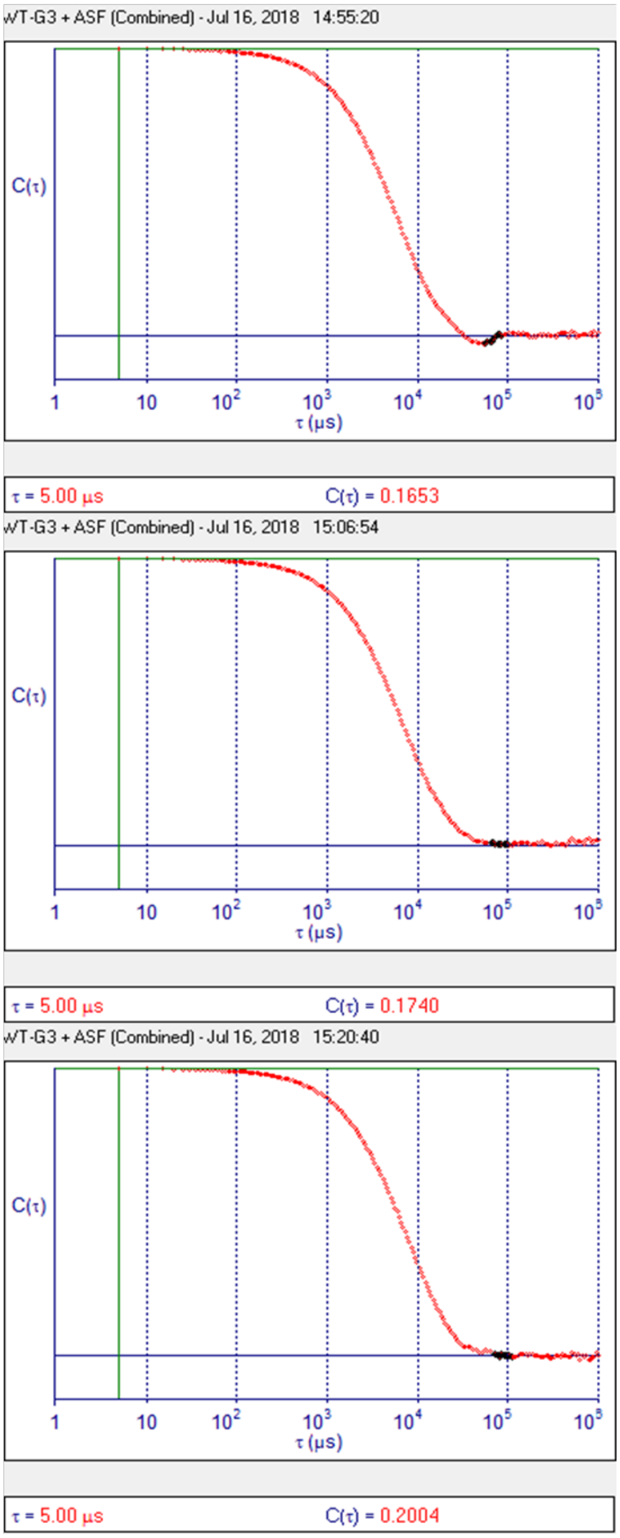

Supplementary Figure 64. Correlation functions for DLS measurements of 10 μM WT-G3 plus 7 μM ASF. Data are technical replicates.

a

Sample ID	NL-G3 + ASF (Combined)
Date - Time	Jul 16, 2018 16:01:57
Operator ID	SF
Elapsed Time	00:05:00
Mean Diam.	13.8 nm
Rel. Var.	0.039
Skew	8.218

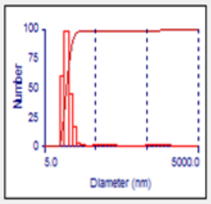

d(nm)	G(d)	C(d)	d(nm)	G(d)	C(d)	d(nm)	G(d)	C(d)
6.4	0	0	47.3	0	100	350.2	0	100
7.7	0	0	56.8	0	100	420.1	0	100
9.2	0	0	68.1	0	100	504.0	0	100
11.0	60	27	81.7	0	100	604.5	0	100
13.2	100	71	98.0	0	100	725.1	0	100
15.9	45	91	117.6	0	100	869.8	0	100
19.1	16	99	141.0	0	100	1043.3	0	100
22.9	2	100	169.2	0	100	1251.5	0	100
27.4	1	100	202.9	0	100	1501.2	0	100
32.9	0	100	243.4	0	100	1800.7	0	100
39.5	0	100	292.0	0	100	2160.0	0	100

Number

Sample ID	NL-G3 + ASF (Combined)
Date - Time	Jul 16, 2018 16:10:10
Operator ID	SF
Elapsed Time	00:05:00
Mean Diam.	9.2 nm
Rel. Var.	0.026
Skew	14.928

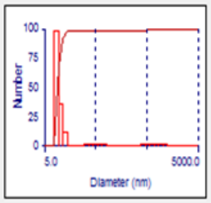

d(nm)	G(d)	C(d)	d(nm)	G(d)	C(d)	d(nm)	G(d)	C(d)
3.9	0	0	33.0	0	100	280.7	0	100
4.7	0	0	40.1	0	100	340.9	0	100
5.7	0	0	48.7	0	100	414.2	0	100
7.0	0	0	59.2	0	100	503.2	0	100
8.5	100	68	71.9	0	100	611.3	0	100
10.3	36	93	87.3	0	100	742.6	0	100
12.5	11	100	106.1	0	100	902.1	0	100
15.2	0	100	128.9	0	100	1095.9	0	100
18.4	0	100	156.5	0	100	1331.3	0	100
22.4	0	100	190.2	0	100	1617.3	0	100
27.2	0	100	231.0	0	100	1964.7	0	100

Number

Sample ID	NL-G3 + ASF (Combined)
Date - Time	Jul 16, 2018 16:22:00
Operator ID	SF
Elapsed Time	00:05:00
Mean Diam.	13.2 nm
Rel. Var.	0.040
Skew	9.769

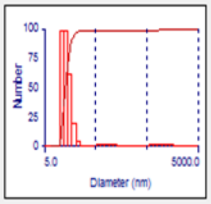

d(nm)	G(d)	C(d)	d(nm)	G(d)	C(d)	d(nm)	G(d)	C(d)
7.5	0	0	55.5	0	100	410.4	0	100
9.0	0	0	66.5	0	100	492.3	0	100
10.8	100	35	79.8	0	100	590.5	0	100
12.9	100	71	95.7	0	100	708.3	0	100
15.5	61	92	114.8	0	100	849.6	0	100
18.6	19	99	137.8	0	100	1019.1	0	100
22.3	4	100	165.3	0	100	1222.5	0	100
26.8	0	100	198.2	0	100	1466.4	0	100
32.1	0	100	237.8	0	100	1759.0	0	100
38.6	0	100	285.2	0	100	2109.9	0	100
46.2	0	100	342.1	0	100	2530.9	0	100

Number

b

Sample ID	NL-G3 + ASF (Combined)
Date - Time	Jul 16, 2018 16:01:57
Operator ID	SF
Elapsed Time	00:05:00
Mean Diam.	42.7 nm
Rel. Var.	11.149
Skew	5.634

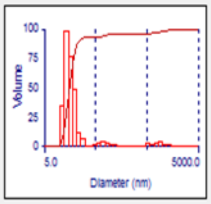

d(nm)	G(d)	C(d)	d(nm)	G(d)	C(d)	d(nm)	G(d)	C(d)
6.4	0	0	47.3	1	94	350.2	0	97
7.7	0	0	56.8	2	95	420.1	0	97
9.2	0	0	68.1	3	96	504.0	2	97
11.0	35	12	81.7	2	96	604.5	1	98
13.2	100	45	98.0	1	97	725.1	2	98
15.9	78	72	117.6	0	97	869.8	4	100
19.1	49	88	141.0	0	97	1043.3	1	100
22.9	11	92	169.2	0	97	1251.5	0	100
27.4	5	94	202.9	0	97	1501.2	0	100
32.9	0	94	243.4	0	97	1800.7	0	100
39.5	0	94	292.0	0	97	2160.0	0	100

Volume

Sample ID	NL-G3 + ASF (Combined)
Date - Time	Jul 16, 2018 16:10:10
Operator ID	SF
Elapsed Time	00:05:00
Mean Diam.	27.7 nm
Rel. Var.	14.125
Skew	6.922

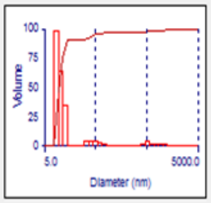

d(nm)	G(d)	C(d)	d(nm)	G(d)	C(d)	d(nm)	G(d)	C(d)
3.9	0	0	33.0	3	93	280.7	0	97
4.7	0	0	40.1	3	95	340.9	0	97
5.7	0	0	48.7	3	96	414.2	0	98
7.0	0	0	59.2	1	97	503.2	3	99
8.5	100	46	71.9	1	97	611.3	0	99
10.3	64	76	87.3	0	97	742.6	1	100
12.5	35	92	106.1	0	97	902.1	0	100
15.2	0	92	128.9	0	97	1095.9	0	100
18.4	0	92	156.5	0	97	1331.3	0	100
22.4	0	92	190.2	0	97	1617.3	0	100
27.2	0	92	231.0	0	97	1964.7	0	100

Volume

Sample ID	NL-G3 + ASF (Combined)
Date - Time	Jul 16, 2018 16:22:00
Operator ID	SF
Elapsed Time	00:05:00
Mean Diam.	64.2 nm
Rel. Var.	11.777
Skew	4.701

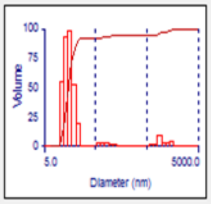

d(nm)	G(d)	C(d)	d(nm)	G(d)	C(d)	d(nm)	G(d)	C(d)
7.5	0	0	55.5	1	93	410.4	0	95
9.0	0	0	66.5	2	94	492.3	0	95
10.8	55	16	79.8	2	95	590.5	0	95
12.9	95	43	95.7	1	95	708.3	1	96
15.5	100	72	114.8	0	95	849.6	9	98
18.6	53	88	137.8	0	95	1019.1	2	99
22.3	18	93	165.3	0	95	1222.5	2	99
26.8	0	93	198.2	0	95	1466.4	3	100
32.1	0	93	237.8	0	95	1759.0	0	100
38.6	0	93	285.2	0	95	2109.9	0	100
46.2	0	93	342.1	0	95	2530.9	0	100

Volume

Supplementary Figure 65. Size distribution of 10 µM NL-G3 plus 7 µM ASF. **a** Number- and **b** volume-weighted. Data in columns are technical replicates of **a** or **b**.

a

Sample ID	NL-G3 + ASF (Combined)
Date - Time	Jul 16, 2018 16:01:57
Operator ID	SF
Elapsed Time	00:05:00
Mean Diam.	15.6 nm
Rel. Var.	1.731
Skew	37.068

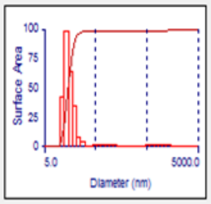

d(nm)	G(d)	C(d)	d(nm)	G(d)	C(d)	d(nm)	G(d)	C(d)
6.4	0	0	47.3	0	99	350.2	0	100
7.7	0	0	56.8	1	100	420.1	0	100
9.2	0	0	68.1	1	100	504.0	0	100
11.0	42	17	81.7	0	100	604.5	0	100
13.2	100	56	98.0	0	100	725.1	0	100
15.9	65	82	117.6	0	100	869.8	0	100
19.1	34	96	141.0	0	100	1043.3	0	100
22.9	7	98	169.2	0	100	1251.5	0	100
27.4	3	99	202.9	0	100	1501.2	0	100
32.9	0	99	243.4	0	100	1800.7	0	100
39.5	0	99	292.0	0	100	2160.0	0	100

Surface Area

Sample ID	NL-G3 + ASF (Combined)
Date - Time	Jul 16, 2018 16:10:10
Operator ID	SF
Elapsed Time	00:05:00
Mean Diam.	10.2 nm
Rel. Var.	1.710
Skew	46.934

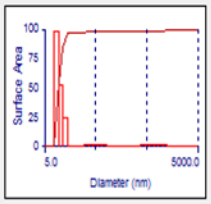

d(nm)	G(d)	C(d)	d(nm)	G(d)	C(d)	d(nm)	G(d)	C(d)
3.9	0	0	33.0	1	99	280.7	0	100
4.7	0	0	40.1	1	99	340.9	0	100
5.7	0	0	48.7	1	100	414.2	0	100
7.0	0	0	59.2	0	100	503.2	0	100
8.5	100	56	71.9	0	100	611.3	0	100
10.3	53	85	87.3	0	100	742.6	0	100
12.5	23	99	106.1	0	100	902.1	0	100
15.2	0	99	128.9	0	100	1095.9	0	100
18.4	0	99	156.5	0	100	1331.3	0	100
22.4	0	99	190.2	0	100	1617.3	0	100
27.2	0	99	231.0	0	100	1964.7	0	100

Surface Area

Sample ID	NL-G3 + ASF (Combined)
Date - Time	Jul 16, 2018 16:22:00
Operator ID	SF
Elapsed Time	00:05:00
Mean Diam.	15.2 nm
Rel. Var.	3.215
Skew	37.530

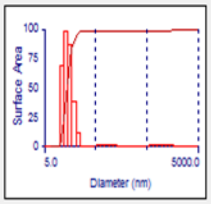

d(nm)	G(d)	C(d)	d(nm)	G(d)	C(d)	d(nm)	G(d)	C(d)
7.5	0	0	55.5	0	100	410.4	0	100
9.0	0	0	66.5	0	100	492.3	0	100
10.8	69	22	79.8	0	100	590.5	0	100
12.9	100	55	95.7	0	100	708.3	0	100
15.5	88	83	114.8	0	100	849.6	0	100
18.6	39	96	137.8	0	100	1019.1	0	100
22.3	11	99	165.3	0	100	1222.5	0	100
26.8	0	99	198.2	0	100	1466.4	0	100
32.1	0	99	237.8	0	100	1759.0	0	100
38.6	0	99	285.2	0	100	2109.9	0	100
46.2	0	99	342.1	0	100	2530.9	0	100

Surface Area

b

Sample ID	NL-G3 + ASF (Combined)
Date - Time	Jul 16, 2018 16:01:57
Operator ID	SF
Elapsed Time	00:05:00
Mean Diam.	561.9 nm
Rel. Var.	0.467
Skew	-0.220

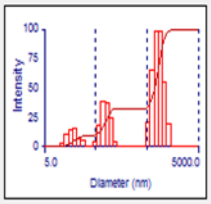

d(nm)	G(d)	C(d)	d(nm)	G(d)	C(d)	d(nm)	G(d)	C(d)
6.4	0	0	47.3	4	10	350.2	0	33
7.7	0	0	56.8	18	14	420.1	0	33
9.2	0	0	68.1	38	21	504.0	20	37
11.0	2	0	81.7	37	28	604.5	66	49
13.2	10	2	98.0	24	32	725.1	100	68
15.9	14	5	117.6	3	33	869.8	99	86
19.1	15	8	141.0	0	33	1043.3	56	96
22.9	6	9	169.2	0	33	1251.5	19	100
27.4	5	10	202.9	0	33	1501.2	0	100
32.9	0	10	243.4	0	33	1800.7	0	100
39.5	0	10	292.0	0	33	2160.0	0	100

Intensity

Sample ID	NL-G3 + ASF (Combined)
Date - Time	Jul 16, 2018 16:10:10
Operator ID	SF
Elapsed Time	00:05:00
Mean Diam.	498.1 nm
Rel. Var.	0.429
Skew	-0.266

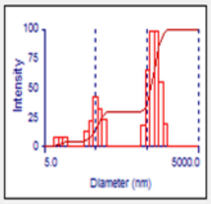

d(nm)	G(d)	C(d)	d(nm)	G(d)	C(d)	d(nm)	G(d)	C(d)
3.9	0	0	33.0	12	7	280.7	0	30
4.7	0	0	40.1	21	11	340.9	0	30
5.7	0	0	48.7	42	19	414.2	18	33
7.0	0	0	59.2	32	25	503.2	65	46
8.5	7	1	71.9	22	30	611.3	100	66
10.3	8	3	87.3	0	30	742.6	100	85
12.5	7	4	106.1	0	30	902.1	56	96
15.2	0	4	128.9	0	30	1095.9	19	100
18.4	0	4	156.5	0	30	1331.3	0	100
22.4	0	4	190.2	0	30	1617.3	0	100
27.2	0	4	231.0	0	30	1964.7	0	100

Intensity

Sample ID	NL-G3 + ASF (Combined)
Date - Time	Jul 16, 2018 16:22:00
Operator ID	SF
Elapsed Time	00:05:00
Mean Diam.	656.4 nm
Rel. Var.	0.454
Skew	-0.249

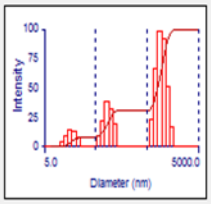

d(nm)	G(d)	C(d)	d(nm)	G(d)	C(d)	d(nm)	G(d)	C(d)
7.5	0	0	55.5	9	11	410.4	0	32
9.0	0	0	66.5	21	15	492.3	0	32
10.8	3	1	79.8	39	22	590.5	23	36
12.9	8	2	95.7	31	28	708.3	67	49
15.5	14	5	114.8	19	32	849.6	100	69
18.6	13	7	137.8	0	32	1019.1	93	87
22.3	8	9	165.3	0	32	1222.5	51	97
26.8	0	9	198.2	0	32	1466.4	16	100
32.1	0	9	237.8	0	32	1759.0	0	100
38.6	0	9	285.2	0	32	2109.9	0	100
46.2	0	9	342.1	0	32	2530.9	0	100

Intensity

Supplementary Figure 66. Size distribution of 10 µM NL-G3 plus 7 µM ASF. **a** Surface area- and **b** intensity-weighted. Data in columns are technical replicates of **a** or **b**.

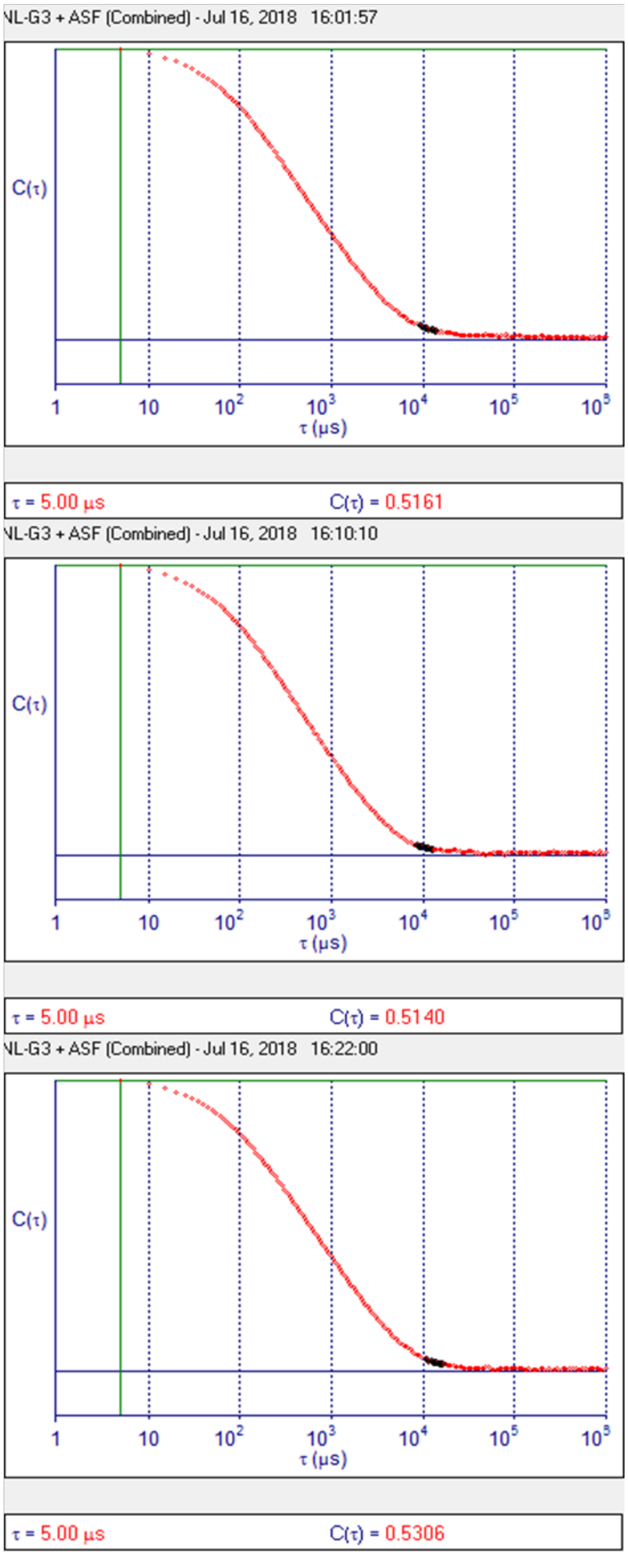

Supplementary Figure 67. Correlation functions for DLS measurements of 10 μM NL-G3 plus 7 μM ASF. Data are technical replicates.

a

Sample ID	NL-TT-G3 + ASF (Combined)
Date - Time	Jul 16, 2018 15:38:36
Operator ID	SF
Elapsed Time	00:05:00
Mean Diam.	294.2 nm
Rel. Var.	3.966
Skew	4.892

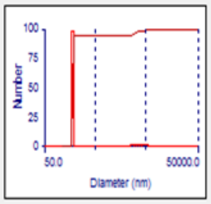

d(nm)	G(d)	C(d)	d(nm)	G(d)	C(d)	d(nm)	G(d)	C(d)
120.2	0	0	486.8	0	96	1970.6	0	96
136.5	0	0	552.8	0	96	2237.7	0	96
155.1	0	0	627.7	0	96	2541.0	1	97
176.1	100	96	712.8	0	96	2885.5	1	98
199.9	0	96	809.4	0	96	3276.6	1	99
227.0	0	96	919.1	0	96	3720.8	1	100
257.8	0	96	1043.7	0	96	4225.1	0	100
292.8	0	96	1185.2	0	96	4797.8	0	100
332.4	0	96	1345.8	0	96	5448.2	0	100
377.5	0	96	1528.2	0	96	6186.7	0	100
428.7	0	96	1735.4	0	96	7025.3	0	100

Number

Sample ID	NL-TT-G3 + ASF (Combined)
Date - Time	Jul 16, 2018 15:46:35
Operator ID	SF
Elapsed Time	00:05:00
Mean Diam.	769.8 nm
Rel. Var.	1.501
Skew	2.038

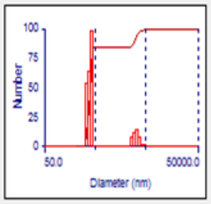

d(nm)	G(d)	C(d)	d(nm)	G(d)	C(d)	d(nm)	G(d)	C(d)
226.7	0	0	833.2	0	85	3062.5	14	97
255.2	0	0	937.9	0	85	3447.2	7	100
287.2	0	0	1055.7	0	85	3880.3	1	100
323.3	54	21	1188.3	0	85	4367.7	0	100
363.9	64	46	1337.6	0	85	4916.4	0	100
409.6	100	85	1505.6	0	85	5534.0	0	100
461.1	0	85	1694.8	0	85	6229.2	0	100
519.0	0	85	1907.7	0	85	7011.7	0	100
584.2	0	85	2147.3	0	85	7892.5	0	100
657.6	0	85	2417.1	7	87	8884.0	0	100
740.2	0	85	2720.7	11	91	10000.0	0	100

Number

Sample ID	NL-TT-G3 + ASF (Combined)
Date - Time	Jul 16, 2018 15:53:35
Operator ID	SF
Elapsed Time	00:05:00
Mean Diam.	328.0 nm
Rel. Var.	2.793
Skew	5.465

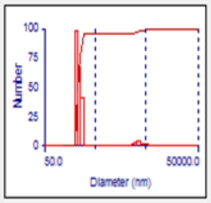

d(nm)	G(d)	C(d)	d(nm)	G(d)	C(d)	d(nm)	G(d)	C(d)
125.6	0	0	505.8	0	97	2037.2	0	97
142.6	0	0	574.1	0	97	2312.3	0	97
161.8	0	0	651.7	0	97	2624.5	1	97
183.7	0	0	739.6	0	97	2978.9	2	98
208.4	100	47	839.5	0	97	3381.1	3	99
236.6	68	78	952.9	0	97	3837.6	1	100
268.5	40	97	1081.5	0	97	4355.7	0	100
304.8	0	97	1227.5	0	97	4943.8	0	100
345.9	0	97	1393.3	0	97	5611.3	0	100
392.7	0	97	1581.4	0	97	6368.9	0	100
445.7	0	97	1794.9	0	97	7228.8	0	100

Number

b

Sample ID	NL-TT-G3 + ASF (Combined)
Date - Time	Jul 16, 2018 15:38:36
Operator ID	SF
Elapsed Time	00:05:00
Mean Diam.	3323.6 nm
Rel. Var.	0.023
Skew	-1.035

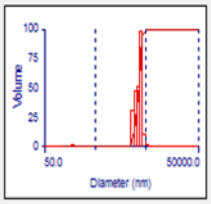

d(nm)	G(d)	C(d)	d(nm)	G(d)	C(d)	d(nm)	G(d)	C(d)
120.2	0	0	486.8	0	0	1970.6	0	0
136.5	0	0	552.8	0	0	2237.7	0	0
155.1	0	0	627.7	0	0	2541.0	30	13
176.1	1	0	712.8	0	0	2885.5	47	32
199.9	0	0	809.4	0	0	3276.6	52	54
227.0	0	0	919.1	0	0	3720.8	100	96
257.8	0	0	1043.7	0	0	4225.1	10	100
292.8	0	0	1185.2	0	0	4797.8	1	100
332.4	0	0	1345.8	0	0	5448.2	0	100
377.5	0	0	1528.2	0	0	6186.7	0	100
428.7	0	0	1735.4	0	0	7025.3	0	100

Volume

Sample ID	NL-TT-G3 + ASF (Combined)
Date - Time	Jul 16, 2018 15:46:35
Operator ID	SF
Elapsed Time	00:05:00
Mean Diam.	3090.6 nm
Rel. Var.	0.027
Skew	-1.166

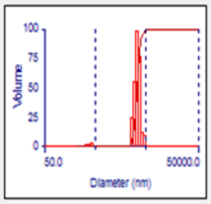

d(nm)	G(d)	C(d)	d(nm)	G(d)	C(d)	d(nm)	G(d)	C(d)
226.7	0	0	833.2	0	1	3062.5	100	65
255.2	0	0	937.9	0	1	3447.2	77	93
287.2	0	0	1055.7	0	1	3880.3	11	97
323.3	0	0	1188.3	0	1	4367.7	9	100
363.9	1	0	1337.6	0	1	4916.4	0	100
409.6	2	1	1505.6	0	1	5534.0	0	100
461.1	0	1	1694.8	0	1	6229.2	0	100
519.0	0	1	1907.7	0	1	7011.7	0	100
584.2	0	1	2147.3	0	1	7892.5	0	100
657.6	0	1	2417.1	24	10	8884.0	0	100
740.2	0	1	2720.7	55	29	10000.0	0	100

Volume

Sample ID	NL-TT-G3 + ASF (Combined)
Date - Time	Jul 16, 2018 15:53:35
Operator ID	SF
Elapsed Time	00:05:00
Mean Diam.	3445.9 nm
Rel. Var.	0.027
Skew	-1.331

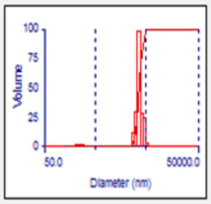

d(nm)	G(d)	C(d)	d(nm)	G(d)	C(d)	d(nm)	G(d)	C(d)
125.6	0	0	505.8	0	1	2037.2	0	1
142.6	0	0	574.1	0	1	2312.3	0	1
161.8	0	0	651.7	0	1	2624.5	11	7
183.7	0	0	739.6	0	1	2978.9	30	22
208.4	1	0	839.5	0	1	3381.1	100	73
236.6	1	1	952.9	0	1	3837.6	28	87
268.5	1	1	1081.5	0	1	4355.7	24	99
304.8	0	1	1227.5	0	1	4943.8	2	100
345.9	0	1	1393.3	0	1	5611.3	0	100
392.7	0	1	1581.4	0	1	6368.9	0	100
445.7	0	1	1794.9	0	1	7228.8	0	100

Volume

Supplementary Figure 68. Size distribution of 10 μM NL-TT-G3 plus 7 μM ASF. **a** Number- and **b** volume-weighted. Data in columns are technical replicates of **a** or **b**.

a

Sample ID	NL-TT-G3 + ASF (Combined)
Date - Time	Jul 16, 2018 15:38:36
Operator ID	SF
Elapsed Time	00:05:00
Mean Diam.	3052.9 nm
Rel. Var.	0.089
Skew	-2.009

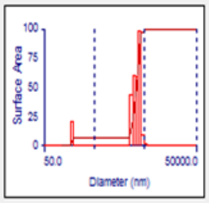

d(nm)	G(d)	C(d)	d(nm)	G(d)	C(d)	d(nm)	G(d)	C(d)
120.2	0	0	486.8	0	7	1970.6	0	7
136.5	0	0	552.8	0	7	2237.7	0	7
155.1	0	0	627.7	0	7	2541.0	44	22
176.1	20	7	712.8	0	7	2885.5	61	43
199.9	0	7	809.4	0	7	3276.6	59	63
227.0	0	7	919.1	0	7	3720.8	100	97
257.8	0	7	1043.7	0	7	4225.1	9	100
292.8	0	7	1185.2	0	7	4797.8	1	100
332.4	0	7	1345.8	0	7	5448.2	0	100
377.5	0	7	1528.2	0	7	6186.7	0	100
428.7	0	7	1735.4	0	7	7025.3	0	100

Surface Area

Sample ID	NL-TT-G3 + ASF (Combined)
Date - Time	Jul 16, 2018 15:46:35
Operator ID	SF
Elapsed Time	00:05:00
Mean Diam.	2847.7 nm
Rel. Var.	0.085
Skew	-1.854

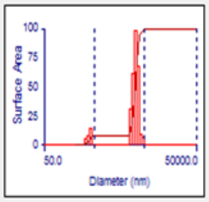

d(nm)	G(d)	C(d)	d(nm)	G(d)	C(d)	d(nm)	G(d)	C(d)
226.7	0	0	833.2	0	8	3062.5	100	72
255.2	0	0	937.9	0	8	3447.2	69	95
287.2	0	0	1055.7	0	8	3880.3	9	98
323.3	4	1	1188.3	0	8	4367.7	6	100
363.9	7	4	1337.6	0	8	4916.4	0	100
409.6	13	8	1505.6	0	8	5534.0	0	100
461.1	0	8	1694.8	0	8	6229.2	0	100
519.0	0	8	1907.7	0	8	7011.7	0	100
584.2	0	8	2147.3	0	8	7892.5	0	100
657.6	0	8	2417.1	31	18	8884.0	0	100
740.2	0	8	2720.7	62	39	10000.0	0	100

Surface Area

Sample ID	NL-TT-G3 + ASF (Combined)
Date - Time	Jul 16, 2018 15:53:35
Operator ID	SF
Elapsed Time	00:05:00
Mean Diam.	3017.1 nm
Rel. Var.	0.142
Skew	-1.625

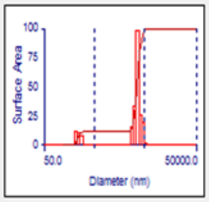

d(nm)	G(d)	C(d)	d(nm)	G(d)	C(d)	d(nm)	G(d)	C(d)
125.6	0	0	505.8	0	13	2037.2	0	13
142.6	0	0	574.1	0	13	2312.3	0	13
161.8	0	0	651.7	0	13	2624.5	14	19
183.7	0	0	739.6	0	13	2978.9	34	34
208.4	11	5	839.5	0	13	3381.1	100	80
236.6	10	9	952.9	0	13	3837.6	25	91
268.5	7	13	1081.5	0	13	4355.7	18	99
304.8	0	13	1227.5	0	13	4943.8	1	100
345.9	0	13	1393.3	0	13	5611.3	0	100
392.7	0	13	1581.4	0	13	6368.9	0	100
445.7	0	13	1794.9	0	13	7228.8	0	100

Surface Area

b

Sample ID	NL-TT-G3 + ASF (Combined)
Date - Time	Jul 16, 2018 15:38:36
Operator ID	SF
Elapsed Time	00:05:00
Mean Diam.	3321.4 nm
Rel. Var.	0.030
Skew	-0.942

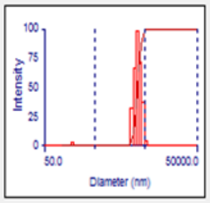

d(nm)	G(d)	C(d)	d(nm)	G(d)	C(d)	d(nm)	G(d)	C(d)
120.2	0	0	486.8	0	1	1970.6	0	1
136.5	0	0	552.8	0	1	2237.7	0	1
155.1	0	0	627.7	0	1	2541.0	32	11
176.1	2	1	712.8	0	1	2885.5	67	32
199.9	0	1	809.4	0	1	3276.6	100	64
227.0	0	1	919.1	0	1	3720.8	72	87
257.8	0	1	1043.7	0	1	4225.1	37	99
292.8	0	1	1185.2	0	1	4797.8	3	100
332.4	0	1	1345.8	0	1	5448.2	0	100
377.5	0	1	1528.2	0	1	6186.7	0	100
428.7	0	1	1735.4	0	1	7025.3	0	100

Intensity

Sample ID	NL-TT-G3 + ASF (Combined)
Date - Time	Jul 16, 2018 15:46:35
Operator ID	SF
Elapsed Time	00:05:00
Mean Diam.	3234.2 nm
Rel. Var.	0.044
Skew	-1.731

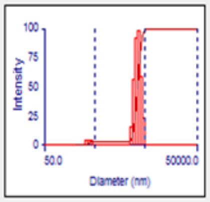

d(nm)	G(d)	C(d)	d(nm)	G(d)	C(d)	d(nm)	G(d)	C(d)
226.7	0	0	833.2	0	3	3062.5	93	49
255.2	0	0	937.9	0	3	3447.2	100	77
287.2	0	0	1055.7	0	3	3880.3	59	94
323.3	3	1	1188.3	0	3	4367.7	22	100
363.9	3	2	1337.6	0	3	4916.4	0	100
409.6	3	3	1505.6	0	3	5534.0	0	100
461.1	0	3	1694.8	0	3	6229.2	0	100
519.0	0	3	1907.7	0	3	7011.7	0	100
584.2	0	3	2147.3	0	3	7892.5	0	100
657.6	0	3	2417.1	15	7	8884.0	0	100
740.2	0	3	2720.7	57	23	10000.0	0	100

Intensity

Sample ID	NL-TT-G3 + ASF (Combined)
Date - Time	Jul 16, 2018 15:53:35
Operator ID	SF
Elapsed Time	00:05:00
Mean Diam.	3474.6 nm
Rel. Var.	0.054
Skew	-1.692

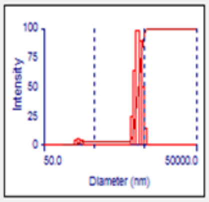

d(nm)	G(d)	C(d)	d(nm)	G(d)	C(d)	d(nm)	G(d)	C(d)
125.6	0	0	505.8	0	3	2037.2	0	3
142.6	0	0	574.1	0	3	2312.3	0	3
161.8	0	0	651.7	0	3	2624.5	24	10
183.7	0	0	739.6	0	3	2978.9	64	28
208.4	4	1	839.5	0	3	3381.1	100	56
236.6	4	2	952.9	0	3	3837.6	90	82
268.5	4	3	1081.5	0	3	4355.7	50	96
304.8	0	3	1227.5	0	3	4943.8	14	100
345.9	0	3	1393.3	0	3	5611.3	0	100
392.7	0	3	1581.4	0	3	6368.9	0	100
445.7	0	3	1794.9	0	3	7228.8	0	100

Intensity

Supplementary Figure 69. Size distribution of 10 µM NL-TT-G3 plus 7 µM ASF. **a** Surface area- and **b** intensity-weighted. Data in columns are technical replicates of **a** or **b**.

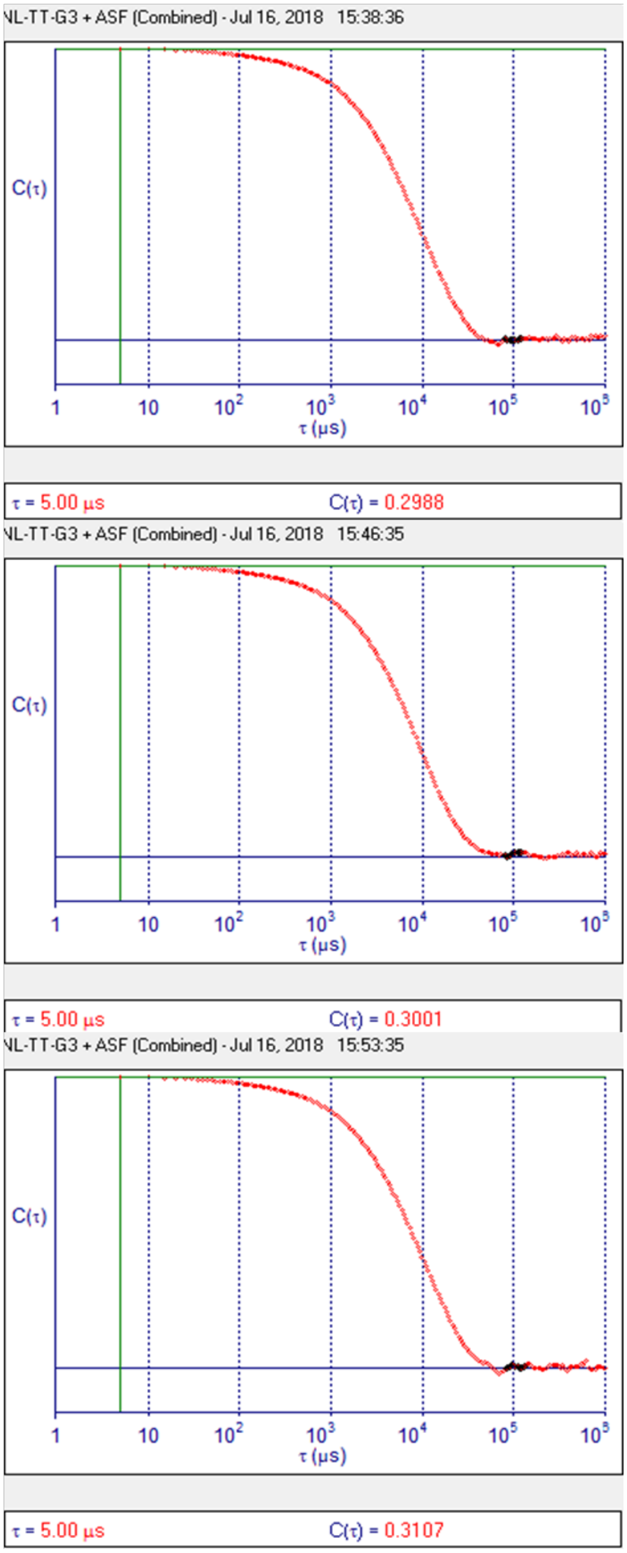

Supplementary Figure 70. Correlation functions for DLS measurements of 10 μM NL-TT-G3 plus 7 μM ASF. Data are technical replicates.

a **b**

Sample ID WT-G3 only (Combined)
Date - Time Jul 16, 2018 16:49:36
Operator ID SF
Elapsed Time 00:05:00
Mean Diam. 5.5 nm
Rel. Var. 0.008
Skew 4.113

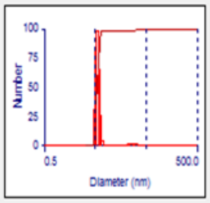

d(nm)	G(d)	C(d)	d(nm)	G(d)	C(d)	d(nm)	G(d)	C(d)
3.5	0	0	8.0	0	100	18.4	0	100
3.7	0	0	8.6	0	100	19.8	0	100
4.0	0	0	9.3	0	100	21.4	0	100
4.4	0	0	10.0	0	100	23.0	0	100
4.7	30	10	10.8	0	100	24.8	0	100
5.1	85	37	11.7	0	100	26.8	0	100
5.5	100	69	12.6	0	100	28.9	0	100
5.9	66	91	13.6	0	100	31.2	0	100
6.4	25	99	14.6	0	100	33.6	0	100
6.9	4	100	15.8	0	100	36.3	0	100
7.4	0	100	17.0	0	100	39.1	0	100

Number

Sample ID WT-G3 only (Combined)
Date - Time Jul 16, 2018 16:49:36
Operator ID SF
Elapsed Time 00:05:00
Mean Diam. 5.7 nm
Rel. Var. 0.074
Skew 11.017

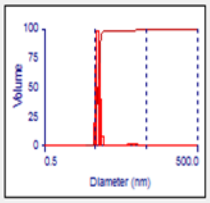

d(nm)	G(d)	C(d)	d(nm)	G(d)	C(d)	d(nm)	G(d)	C(d)
3.5	0	0	8.0	0	99	18.4	0	99
3.7	0	0	8.6	0	99	19.8	0	99
4.0	0	0	9.3	0	99	21.4	0	100
4.4	0	0	10.0	0	99	23.0	0	100
4.7	19	6	10.8	0	99	24.8	1	100
5.1	67	27	11.7	0	99	26.8	0	100
5.5	100	59	12.6	0	99	28.9	0	100
5.9	83	85	13.6	0	99	31.2	0	100
6.4	40	97	14.6	0	99	33.6	0	100
6.9	7	99	15.8	0	99	36.3	0	100
7.4	0	99	17.0	0	99	39.1	0	100

Volume

Sample ID WT-G3 only (Combined)
Date - Time Jul 16, 2018 16:56:35
Operator ID SF
Elapsed Time 00:05:00
Mean Diam. 7.2 nm
Rel. Var. 0.000
Skew 0.030

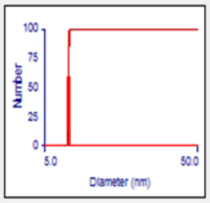

d(nm)	G(d)	C(d)	d(nm)	G(d)	C(d)	d(nm)	G(d)	C(d)
6.7	0	0	7.2	100	78	7.8	0	100
6.7	0	0	7.3	59	94	7.9	0	100
6.8	0	0	7.3	21	100	7.9	0	100
6.8	0	0	7.4	0	100	8.0	0	100
6.9	0	0	7.4	0	100	8.0	0	100
6.9	0	0	7.5	0	100	8.1	0	100
7.0	0	0	7.5	0	100	8.1	0	100
7.0	0	0	7.6	0	100	8.2	0	100
7.1	18	5	7.6	0	100	8.3	0	100
7.1	59	22	7.7	0	100	8.3	0	100
7.2	97	49	7.7	0	100	8.4	0	100

Number

Sample ID WT-G3 only (Combined)
Date - Time Jul 16, 2018 16:56:35
Operator ID SF
Elapsed Time 00:05:00
Mean Diam. 7.2 nm
Rel. Var. 0.000
Skew 0.014

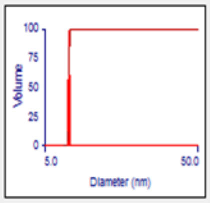

d(nm)	G(d)	C(d)	d(nm)	G(d)	C(d)	d(nm)	G(d)	C(d)
6.7	0	0	7.2	100	77	7.8	0	100
6.7	0	0	7.3	60	94	7.9	0	100
6.8	0	0	7.3	22	100	7.9	0	100
6.8	0	0	7.4	0	100	8.0	0	100
6.9	0	0	7.4	0	100	8.0	0	100
6.9	0	0	7.5	0	100	8.1	0	100
7.0	0	0	7.5	0	100	8.1	0	100
7.0	0	0	7.6	0	100	8.2	0	100
7.1	17	5	7.6	0	100	8.3	0	100
7.1	57	21	7.7	0	100	8.3	0	100
7.2	95	48	7.7	0	100	8.4	0	100

Volume

Sample ID WT-G3 only (Combined)
Date - Time Jul 16, 2018 17:08:55
Operator ID SF
Elapsed Time 00:05:00
Mean Diam. 6.0 nm
Rel. Var. 0.018
Skew 0.861

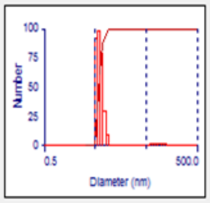

d(nm)	G(d)	C(d)	d(nm)	G(d)	C(d)	d(nm)	G(d)	C(d)
3.5	0	0	13.7	0	100	53.4	0	100
4.0	0	0	15.5	0	100	60.5	0	100
4.5	0	0	17.6	0	100	68.4	0	100
5.1	92	30	19.9	0	100	77.4	0	100
5.8	100	62	22.5	0	100	87.6	0	100
6.5	80	88	25.4	0	100	99.1	0	100
7.4	29	97	28.8	0	100	112.2	0	100
8.4	8	100	32.6	0	100	127.0	0	100
9.5	0	100	36.9	0	100	143.7	0	100
10.7	0	100	41.7	0	100	162.6	0	100
12.1	0	100	47.2	0	100	184.0	0	100

Number

Sample ID WT-G3 only (Combined)
Date - Time Jul 16, 2018 17:08:55
Operator ID SF
Elapsed Time 00:05:00
Mean Diam. 6.3 nm
Rel. Var. 0.039
Skew 30.962

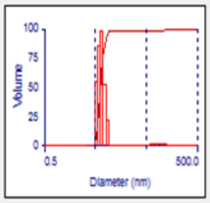

d(nm)	G(d)	C(d)	d(nm)	G(d)	C(d)	d(nm)	G(d)	C(d)
3.5	0	0	13.7	0	100	53.4	0	100
4.0	0	0	15.5	0	100	60.5	0	100
4.5	0	0	17.6	0	100	68.4	0	100
5.1	55	17	19.9	0	100	77.4	0	100
5.8	86	45	22.5	0	100	87.6	0	100
6.5	100	76	25.4	0	100	99.1	0	100
7.4	53	93	28.8	0	100	112.2	0	100
8.4	21	100	32.6	0	100	127.0	0	100
9.5	0	100	36.9	0	100	143.7	0	100
10.7	0	100	41.7	0	100	162.6	0	100
12.1	0	100	47.2	0	100	184.0	0	100

Volume

Supplementary Figure 71. Size distribution of 10 µM WT-G3. **A** Number- and **b** volume-weighted. Data in columns are technical replicates of **a** or **b**.

a

Sample ID	WT-G3 only (Combined)
Date - Time	Jul 16, 2018 16:49:36
Operator ID	SF
Elapsed Time	00:05:00
Mean Diam.	5.6 nm
Rel. Var.	0.024
Skew	14.984

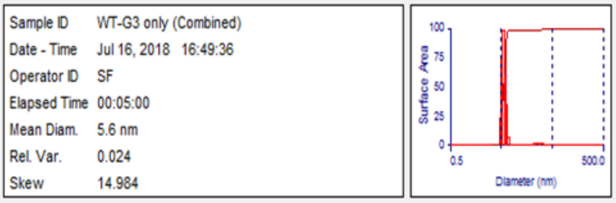

d(nm)	G(d)	C(d)	d(nm)	G(d)	C(d)	d(nm)	G(d)	C(d)
3.5	0	0	8.0	0	100	18.4	0	100
3.7	0	0	8.6	0	100	19.8	0	100
4.0	0	0	9.3	0	100	21.4	0	100
4.4	0	0	10.0	0	100	23.0	0	100
4.7	22	7	10.8	0	100	24.8	0	100
5.1	73	30	11.7	0	100	26.8	0	100
5.5	100	62	12.6	0	100	28.9	0	100
5.9	77	87	13.6	0	100	31.2	0	100
6.4	34	98	14.6	0	100	33.6	0	100
6.9	6	100	15.8	0	100	36.3	0	100
7.4	0	100	17.0	0	100	39.1	0	100

Surface Area

Sample ID	WT-G3 only (Combined)
Date - Time	Jul 16, 2018 16:56:35
Operator ID	SF
Elapsed Time	00:05:00
Mean Diam.	7.2 nm
Rel. Var.	0.000
Skew	0.019

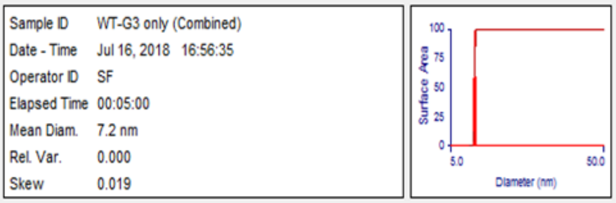

d(nm)	G(d)	C(d)	d(nm)	G(d)	C(d)	d(nm)	G(d)	C(d)
6.7	0	0	7.2	100	77	7.8	0	100
6.7	0	0	7.3	59	94	7.9	0	100
6.8	0	0	7.3	21	100	7.9	0	100
6.8	0	0	7.4	0	100	8.0	0	100
6.9	0	0	7.4	0	100	8.0	0	100
6.9	0	0	7.5	0	100	8.1	0	100
7.0	0	0	7.5	0	100	8.1	0	100
7.0	0	0	7.6	0	100	8.2	0	100
7.1	17	5	7.6	0	100	8.3	0	100
7.1	58	21	7.7	0	100	8.3	0	100
7.2	96	49	7.7	0	100	8.4	0	100

Surface Area

Sample ID	WT-G3 only (Combined)
Date - Time	Jul 16, 2018 17:08:55
Operator ID	SF
Elapsed Time	00:05:00
Mean Diam.	6.2 nm
Rel. Var.	0.022
Skew	6.307

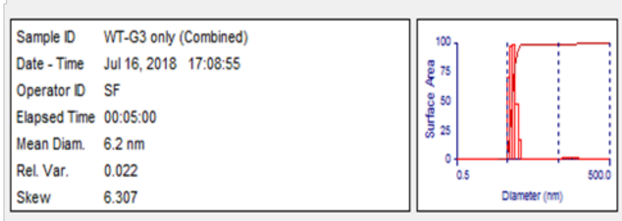

d(nm)	G(d)	C(d)	d(nm)	G(d)	C(d)	d(nm)	G(d)	C(d)
3.5	0	0	13.7	0	100	53.4	0	100
4.0	0	0	15.5	0	100	60.5	0	100
4.5	0	0	17.6	0	100	68.4	0	100
5.1	70	21	19.9	0	100	77.4	0	100
5.8	97	51	22.5	0	100	87.6	0	100
6.5	100	81	25.4	0	100	99.1	0	100
7.4	47	95	28.8	0	100	112.2	0	100
8.4	16	100	32.6	0	100	127.0	0	100
9.5	0	100	36.9	0	100	143.7	0	100
10.7	0	100	41.7	0	100	162.6	0	100
12.1	0	100	47.2	0	100	184.0	0	100

Surface Area

b

Sample ID	WT-G3 only (Combined)
Date - Time	Jul 16, 2018 16:49:36
Operator ID	SF
Elapsed Time	00:05:00
Mean Diam.	12.2 nm
Rel. Var.	0.572
Skew	0.771

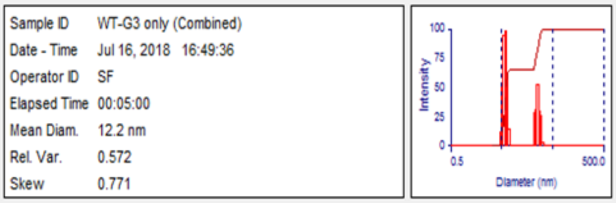

d(nm)	G(d)	C(d)	d(nm)	G(d)	C(d)	d(nm)	G(d)	C(d)
3.5	0	0	8.0	0	66	18.4	0	66
3.7	0	0	8.6	0	66	19.8	0	66
4.0	0	0	9.3	0	66	21.4	28	72
4.4	0	0	10.0	0	66	23.0	30	78
4.7	12	2	10.8	0	66	24.8	53	89
5.1	52	13	11.7	0	66	26.8	30	95
5.5	96	32	12.6	0	66	28.9	25	100
5.9	100	52	13.6	0	66	31.2	2	100
6.4	60	64	14.6	0	66	33.6	0	100
6.9	13	66	15.8	0	66	36.3	0	100
7.4	0	66	17.0	0	66	39.1	0	100

Intensity

Sample ID	WT-G3 only (Combined)
Date - Time	Jul 16, 2018 16:56:35
Operator ID	SF
Elapsed Time	00:05:00
Mean Diam.	7.2 nm
Rel. Var.	0.000
Skew	-0.001

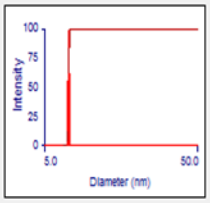

d(nm)	G(d)	C(d)	d(nm)	G(d)	C(d)	d(nm)	G(d)	C(d)
6.7	0	0	7.2	100	76	7.8	0	100
6.7	0	0	7.3	61	94	7.9	0	100
6.8	0	0	7.3	22	100	7.9	0	100
6.8	0	0	7.4	0	100	8.0	0	100
6.9	0	0	7.4	0	100	8.0	0	100
6.9	0	0	7.5	0	100	8.1	0	100
7.0	0	0	7.5	0	100	8.1	0	100
7.0	0	0	7.6	0	100	8.2	0	100
7.1	16	5	7.6	0	100	8.3	0	100
7.1	55	20	7.7	0	100	8.3	0	100
7.2	93	47	7.7	0	100	8.4	0	100

Intensity

Sample ID	WT-G3 only (Combined)
Date - Time	Jul 16, 2018 17:08:55
Operator ID	SF
Elapsed Time	00:05:00
Mean Diam.	23.2 nm
Rel. Var.	2.113
Skew	1.659

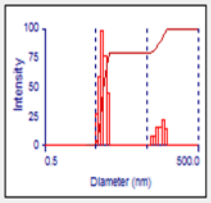

d(nm)	G(d)	C(d)	d(nm)	G(d)	C(d)	d(nm)	G(d)	C(d)
3.5	0	0	13.7	0	80	53.4	0	80
4.0	0	0	15.5	0	80	60.5	7	82
4.5	0	0	17.6	0	80	68.4	7	83
5.1	26	7	19.9	0	80	77.4	14	87
5.8	59	22	22.5	0	80	87.6	15	91
6.5	100	48	25.4	0	80	99.1	21	97
7.4	77	68	28.8	0	80	112.2	13	100
8.4	44	80	32.6	0	80	127.0	0	100
9.5	0	80	36.9	0	80	143.7	0	100
10.7	0	80	41.7	0	80	162.6	0	100
12.1	0	80	47.2	0	80	184.0	0	100

Intensity

Supplementary Figure 72. Size distribution of 10 μM WT-G3. **a** Surface area- and **b** intensity-weighted. Data in columns are technical replicates of **a** or **b**.

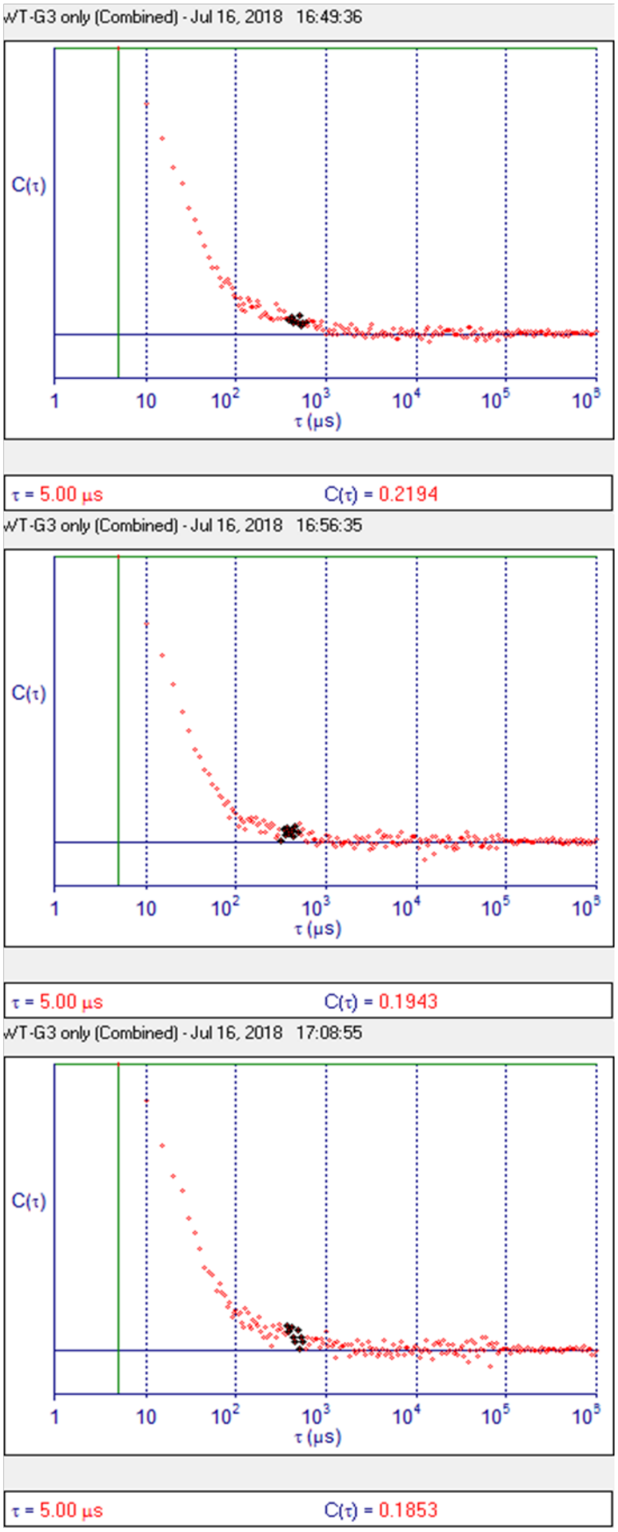

Supplementary Figure 73. Correlation functions for DLS measurements of 10 µM WT-G3. Data are technical replicates.

a

Sample ID	ASF only (Combined)
Date - Time	Jul 16, 2018 17:16:56
Operator ID	SF
Elapsed Time	00:05:00
Mean Diam.	12.2 nm
Rel. Var.	0.013
Skew	4.506

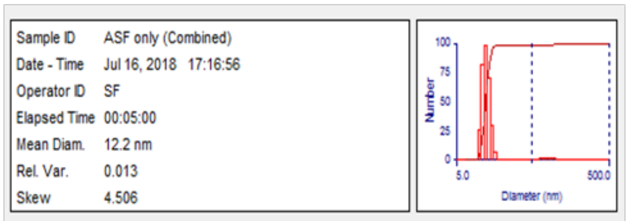

d(nm)	G(d)	C(d)	d(nm)	G(d)	C(d)	d(nm)	G(d)	C(d)
6.9	0	0	19.2	0	100	53.2	0	100
7.6	0	0	21.1	0	100	58.3	0	100
8.4	0	0	23.1	0	100	64.0	0	100
9.2	0	0	25.4	0	100	70.2	0	100
10.0	26	8	27.8	0	100	77.0	0	100
11.0	82	35	30.5	0	100	84.5	0	100
12.1	100	66	33.5	0	100	92.6	0	100
13.3	71	89	36.7	0	100	101.6	0	100
14.6	29	98	40.3	0	100	111.5	0	100
16.0	6	100	44.2	0	100	122.3	0	100
17.5	0	100	48.5	0	100	134.2	0	100

Number

Sample ID	ASF only (Combined)
Date - Time	Jul 16, 2018 17:24:01
Operator ID	SF
Elapsed Time	00:05:00
Mean Diam.	1.9 nm
Rel. Var.	0.021
Skew	6.022

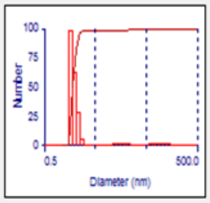

d(nm)	G(d)	C(d)	d(nm)	G(d)	C(d)	d(nm)	G(d)	C(d)
1.2	0	0	7.1	0	100	41.7	0	100
1.4	0	0	8.3	0	100	49.0	0	100
1.7	100	51	9.8	0	100	57.6	0	100
1.9	63	83	11.5	0	100	67.6	0	100
2.3	27	97	13.5	0	100	79.5	0	100
2.7	5	100	15.9	0	100	93.4	0	100
3.2	0	100	18.6	0	100	109.7	0	100
3.7	0	100	21.9	0	100	128.9	0	100
4.4	0	100	25.7	0	100	151.4	0	100
5.1	0	100	30.2	0	100	177.9	0	100
6.0	0	100	35.5	0	100	209.1	0	100

Number

Sample ID	ASF only (Combined)
Date - Time	Jul 16, 2018 17:30:55
Operator ID	SF
Elapsed Time	00:05:00
Mean Diam.	1.2 nm
Rel. Var.	0.026
Skew	3.062

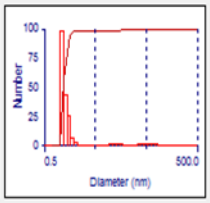

d(nm)	G(d)	C(d)	d(nm)	G(d)	C(d)	d(nm)	G(d)	C(d)
0.8	0	0	4.5	0	100	25.2	0	100
0.9	0	0	5.2	0	100	29.5	0	100
1.1	100	57	6.1	0	100	34.5	0	100
1.3	43	81	7.2	0	100	40.4	0	100
1.5	25	96	8.4	0	100	47.3	0	100
1.7	5	99	9.8	0	100	55.3	0	100
2.0	2	100	11.5	0	100	64.8	0	100
2.4	0	100	13.4	0	100	75.8	0	100
2.8	0	100	15.7	0	100	88.7	0	100
3.3	0	100	18.4	0	100	103.8	0	100
3.8	0	100	21.5	0	100	121.5	0	100

Number

b

Sample ID	ASF only (Combined)
Date - Time	Jul 16, 2018 17:16:56
Operator ID	SF
Elapsed Time	00:05:00
Mean Diam.	13.3 nm
Rel. Var.	0.231
Skew	9.316

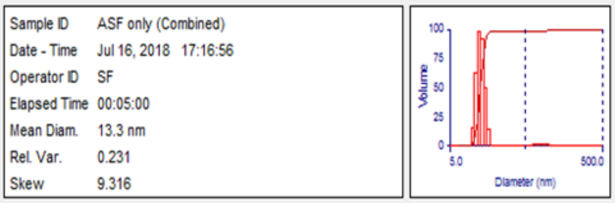

d(nm)	G(d)	C(d)	d(nm)	G(d)	C(d)	d(nm)	G(d)	C(d)
6.9	0	0	19.2	0	99	53.2	0	99
7.6	0	0	21.1	0	99	58.3	0	99
8.4	0	0	23.1	0	99	64.0	1	99
9.2	0	0	25.4	0	99	70.2	1	99
10.0	15	4	27.8	0	99	77.0	1	100
11.0	62	23	30.5	0	99	84.5	1	100
12.1	100	52	33.5	0	99	92.6	0	100
13.3	93	80	36.7	0	99	101.6	0	100
14.6	50	95	40.3	0	99	111.5	0	100
16.0	14	99	44.2	0	99	122.3	0	100
17.5	0	99	48.5	0	99	134.2	0	100

Volume

Sample ID	ASF only (Combined)
Date - Time	Jul 16, 2018 17:24:01
Operator ID	SF
Elapsed Time	00:05:00
Mean Diam.	2.3 nm
Rel. Var.	1.069
Skew	14.223

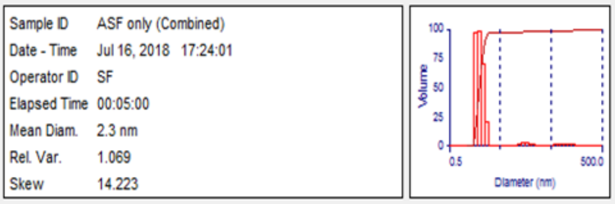

d(nm)	G(d)	C(d)	d(nm)	G(d)	C(d)	d(nm)	G(d)	C(d)
1.2	0	0	7.1	0	98	41.7	0	100
1.4	0	0	8.3	0	98	49.0	0	100
1.7	98	33	9.8	0	98	57.6	0	100
1.9	100	67	11.5	1	98	67.6	0	100
2.3	70	91	13.5	2	99	79.5	0	100
2.7	21	98	15.9	2	100	93.4	0	100
3.2	0	98	18.6	1	100	109.7	0	100
3.7	0	98	21.9	0	100	128.9	0	100
4.4	0	98	25.7	0	100	151.4	0	100
5.1	0	98	30.2	0	100	177.9	0	100
6.0	0	98	35.5	0	100	209.1	0	100

Volume

Sample ID	ASF only (Combined)
Date - Time	Jul 16, 2018 17:30:55
Operator ID	SF
Elapsed Time	00:05:00
Mean Diam.	1.4 nm
Rel. Var.	0.753
Skew	21.965

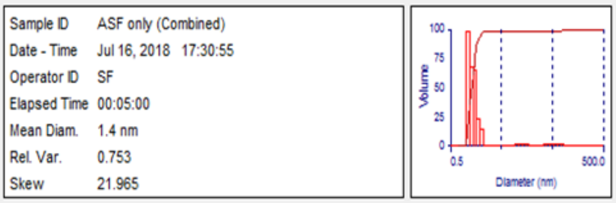

d(nm)	G(d)	C(d)	d(nm)	G(d)	C(d)	d(nm)	G(d)	C(d)
0.8	0	0	4.5	0	99	25.2	0	100
0.9	0	0	5.2	0	99	29.5	0	100
1.1	100	37	6.1	0	99	34.5	0	100
1.3	69	62	7.2	0	99	40.4	0	100
1.5	65	86	8.4	0	99	47.3	0	100
1.7	22	94	9.8	1	99	55.3	0	100
2.0	14	99	11.5	1	100	64.8	0	100
2.4	0	99	13.4	1	100	75.8	0	100
2.8	0	99	15.7	0	100	88.7	0	100
3.3	0	99	18.4	0	100	103.8	0	100
3.8	0	99	21.5	0	100	121.5	0	100

Volume

Supplementary Figure 74. Size distribution of 7 μM ASF. **a** Number- and **b** volume-weighted. Data in columns are technical replicates of **a** or **b**.

a

Sample ID ASF only (Combined)
Date - Time Jul 16, 2018 17:16:56
Operator ID SF
Elapsed Time 00:05:00
Mean Diam. 12.6 nm
Rel. Var. 0.053
Skew 16.712

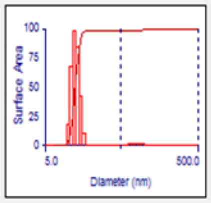

d(nm)	G(d)	C(d)	d(nm)	G(d)	C(d)	d(nm)	G(d)	C(d)
6.9	0	0	19.2	0	100	53.2	0	100
7.6	0	0	21.1	0	100	58.3	0	100
8.4	0	0	23.1	0	100	64.0	0	100
9.2	0	0	25.4	0	100	70.2	0	100
10.0	18	6	27.8	0	100	77.0	0	100
11.0	68	27	30.5	0	100	84.5	0	100
12.1	100	58	33.5	0	100	92.6	0	100
13.3	85	84	36.7	0	100	101.6	0	100
14.6	42	97	40.3	0	100	111.5	0	100
16.0	10	100	44.2	0	100	122.3	0	100
17.5	0	100	48.5	0	100	134.2	0	100

Surface Area

Sample ID ASF only (Combined)
Date - Time Jul 16, 2018 17:24:01
Operator ID SF
Elapsed Time 00:05:00
Mean Diam. 2.0 nm
Rel. Var. 0.156
Skew 21.044

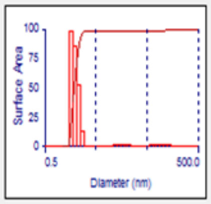

d(nm)	G(d)	C(d)	d(nm)	G(d)	C(d)	d(nm)	G(d)	C(d)
1.2	0	0	7.1	0	100	41.7	0	100
1.4	0	0	8.3	0	100	49.0	0	100
1.7	100	40	9.8	0	100	57.6	0	100
1.9	87	74	11.5	0	100	67.6	0	100
2.3	52	95	13.5	0	100	79.5	0	100
2.7	13	100	15.9	0	100	93.4	0	100
3.2	0	100	18.6	0	100	109.7	0	100
3.7	0	100	21.9	0	100	128.9	0	100
4.4	0	100	25.7	0	100	151.4	0	100
5.1	0	100	30.2	0	100	177.9	0	100
6.0	0	100	35.5	0	100	209.1	0	100

Surface Area

Sample ID ASF only (Combined)
Date - Time Jul 16, 2018 17:30:55
Operator ID SF
Elapsed Time 00:05:00
Mean Diam. 1.3 nm
Rel. Var. 0.096
Skew 27.341

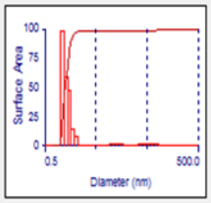

d(nm)	G(d)	C(d)	d(nm)	G(d)	C(d)	d(nm)	G(d)	C(d)
0.8	0	0	4.5	0	100	25.2	0	100
0.9	0	0	5.2	0	100	29.5	0	100
1.1	100	44	6.1	0	100	34.5	0	100
1.3	59	70	7.2	0	100	40.4	0	100
1.5	48	91	8.4	0	100	47.3	0	100
1.7	14	97	9.8	0	100	55.3	0	100
2.0	7	100	11.5	0	100	64.8	0	100
2.4	0	100	13.4	0	100	75.8	0	100
2.8	0	100	15.7	0	100	88.7	0	100
3.3	0	100	18.4	0	100	103.8	0	100
3.8	0	100	21.5	0	100	121.5	0	100

Surface Area

b

Sample ID ASF only (Combined)
Date - Time Jul 16, 2018 17:16:56
Operator ID SF
Elapsed Time 00:05:00
Mean Diam. 54.7 nm
Rel. Var. 0.322
Skew -0.497

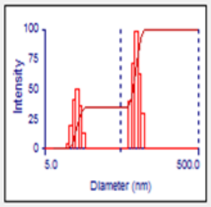

d(nm)	G(d)	C(d)	d(nm)	G(d)	C(d)	d(nm)	G(d)	C(d)
6.9	0	0	19.2	0	35	53.2	0	35
7.6	0	0	21.1	0	35	58.3	0	35
8.4	0	0	23.1	0	35	64.0	39	43
9.2	0	0	25.4	0	35	70.2	71	59
10.0	3	1	27.8	0	35	77.0	100	80
11.0	19	5	30.5	0	35	84.5	62	94
12.1	41	14	33.5	0	35	92.6	30	100
13.3	50	24	36.7	0	35	101.6	0	100
14.6	35	32	40.3	0	35	111.5	0	100
16.0	13	35	44.2	0	35	122.3	0	100
17.5	0	35	48.5	0	35	134.2	0	100

Intensity

Sample ID ASF only (Combined)
Date - Time Jul 16, 2018 17:24:01
Operator ID SF
Elapsed Time 00:05:00
Mean Diam. 54.7 nm
Rel. Var. 0.384
Skew -0.086

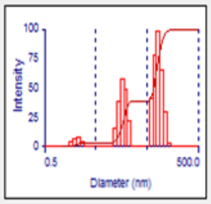

d(nm)	G(d)	C(d)	d(nm)	G(d)	C(d)	d(nm)	G(d)	C(d)
1.2	0	0	7.1	0	4	41.7	0	39
1.4	0	0	8.3	0	4	49.0	0	39
1.7	4	1	9.8	0	4	57.6	41	47
1.9	6	2	11.5	14	7	67.6	78	62
2.3	7	3	13.5	38	14	79.5	100	81
2.7	3	4	15.9	58	25	93.4	65	94
3.2	0	4	18.6	49	35	109.7	30	100
3.7	0	4	21.9	22	39	128.9	2	100
4.4	0	4	25.7	0	39	151.4	0	100
5.1	0	4	30.2	0	39	177.9	0	100
6.0	0	4	35.5	0	39	209.1	0	100

Intensity

Sample ID ASF only (Combined)
Date - Time Jul 16, 2018 17:30:55
Operator ID SF
Elapsed Time 00:05:00
Mean Diam. 39.9 nm
Rel. Var. 0.287
Skew -0.409

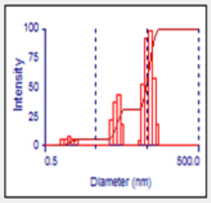

d(nm)	G(d)	C(d)	d(nm)	G(d)	C(d)	d(nm)	G(d)	C(d)
0.8	0	0	4.5	0	5	25.2	0	31
0.9	0	0	5.2	0	5	29.5	0	31
1.1	5	1	6.1	0	5	34.5	4	32
1.3	5	2	7.2	0	5	40.4	52	43
1.5	8	4	8.4	0	5	47.3	93	63
1.7	4	5	9.8	22	10	55.3	100	84
2.0	4	5	11.5	37	18	64.8	58	96
2.4	0	5	13.4	44	27	75.8	17	100
2.8	0	5	15.7	17	31	88.7	0	100
3.3	0	5	18.4	0	31	103.8	0	100
3.8	0	5	21.5	0	31	121.5	0	100

Intensity

Supplementary Figure 75. Size distribution of 7 µM ASF. **a** Surface area- and **b** intensity-weighted. Data in columns are technical replicates of **a** or **b**.

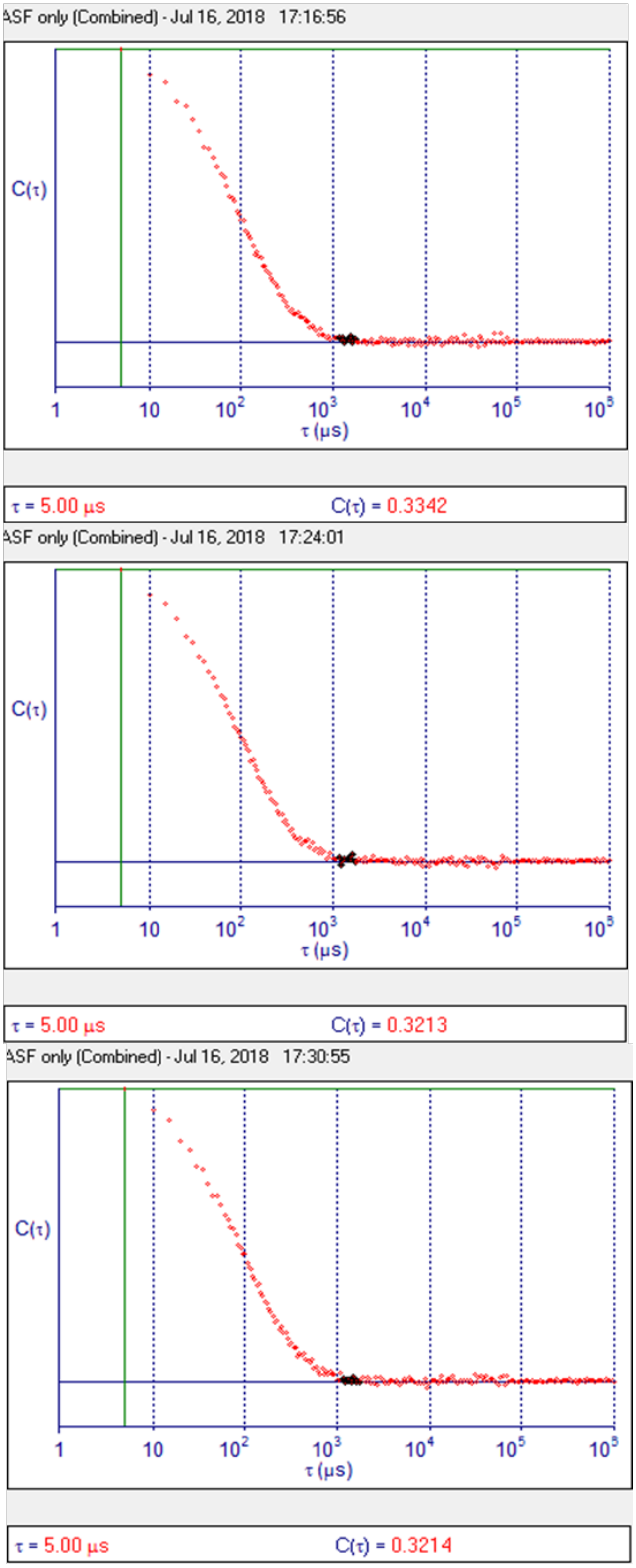

Supplementary Figure 76. Correlation functions for DLS measurements of 7 µM ASF. Data are technical replicates.

Supplementary Note 1. Genetic sequences

NL-TT-G3 genetic sequence:

CC**ATG**GCGGTCTTCACACTCGAAGATTTCGTTGGGGACTGGCGACAGACAGCCGGCTACAACCTGGACCAAGTCCT
TGAACAGGGAGGTGTGTCCAGTTTGTTTCAGAATCTCGGGGTGTCCGTAACTCCGATCCAAAGGATTGTCCTGAGC
GGTGAAAATGGGCTGAAGATCGACATCCATGTCATCATCCCGTATGAAGGTCTGAGCGGCGACCAAATGGGCCAGA
TCGAAAAAATTTTTAAGGTGGTGTACCCTGTGGATGATCATCACTTTAAGGTGATCCTGCACTATGGCACACTGGT
AATCGACGGGGTTACGCCGAACATGATCGACTATTTCGGACGGCCGTATGAAGGCATCGCCGTGTTCGACGGCAAA
AAGATCACTGTAACAGGGACCCTGTGGAACGGCAACAAAATTATCGACGAGCGCCTGATCAACCCCGACGGCTCCC
TGCTGTTCCGAGTAACCATCAACGGAGTGACCGGCTGGCGGCTGTGCGAACGCATTCTGGCGGGATCCGGCGGCGG
CAGCGGCGGCAGCGGCGGCAGCGGCGGCATGGCGCGCATGAAACAGCTGGAAGATAAAGTGGAAGAACTGCTGAGC
AAAAACTATCATCTGGAAAACCGCGTGGCGCGCCTGGAAAAACTGGTGGGCGAACGCGGCGGCGGCAGCGGCGGCA
GCGGCGGCGGCGGCAGCGGCGGCAGCGGCGAATTCGCAGACAATTTTTCGCTCCATGATGCGTTATCTGGGTCTGG
AAACCCAAACCCTCAAGGATGGCCTGGCGCATGGGGGAACCAGCCTGCTGGGGCAGGGGGCTACCCAGGGGCTTCC
TATCCTGGGGCCTACCCCGGGCAGGCACCCCCAGGGGCTTATCCTGGACAGGCACCTCCAGGCGCCTACCCTGGAGC
ACCTGGAGCTTATCCCGGAGCACCTGCACCTGGAGTCTACCCAGGGCCACCCAGCGGCCCTGGGGCCTACCCATCTT
CTGGACAGCCAAGTGCCCCCGGAGCCTACCCTGCCACTGGCCCCTATGGCGCCCCTGCTGGGCCACTGATTGTGCCT
TATAACCTGCCTTTGCCTGGGGGAGTGGTGCCTCGCATGCTGATAACAATTCTGGGCACGGTGAAGCCCAATGCAA
ACAGAATTGCTTTAGATTTCCAAAGAGGGAATGATGTTGCCTTCCACTTTAACCCACGCTTCAATGAGAACAACAG
GAGAGTCATTGTTTGCAATACAAAGCTGGATAATAACTGGGGAAGGGAAGAAAGACAGTCGGTTTTCCCATTTGA
AAGTGGGAAACCATTCAAAATACAAGTACTGGTTGAACCTGACCACTTCAAGGTTGCAGTGAATGATGCTCACTTG
TTGCAGTACAATCATCGGGTTAAAAAACTCAATGAAATCAGCAAACTGGGAATTTCTGGTGACATAGACCTCACCA
GTGCTTCATATAACATGATACTCGAGCACCACCACCACCACCAC**TGA**

NL-G3 genetic sequence:

CC**ATG**GCGGTCTTCACACTCGAAGATTTCGTTGGGGACTGGCGACAGACAGCCGGCTACAACCTGGACCAAGTCCT
TGAACAGGGAGGTGTGTCCAGTTTGTTTCAGAATCTCGGGGTGTCCGTAACTCCGATCCAAAGGATTGTCCTGAGC
GGTGAAAATGGGCTGAAGATCGACATCCATGTCATCATCCCGTATGAAGGTCTGAGCGGCGACCAAATGGGCCAGA
TCGAAAAAATTTTTAAGGTGGTGTACCCTGTGGATGATCATCACTTTAAGGTGATCCTGCACTATGGCACACTGGT
AATCGACGGGGTTACGCCGAACATGATCGACTATTTCGGACGGCCGTATGAAGGCATCGCCGTGTTCGACGGCAAA
AAGATCACTGTAACAGGGACCCTGTGGAACGGCAACAAAATTATCGACGAGCGCCTGATCAACCCCGACGGCTCCC
TGCTGTTCCGAGTAACCATCAACGGAGTGACCGGCTGGCGGCTGTGCGAACGCATTCTGGCGGGATCCGGCGGCGG
CAGCGGCGGCAGCGGCGGCAGCGGCGGCGAATTCGCAGACAATTTTTCGCTCCATGATGCGTTATCTGGGTCTGGA
AACCCAAACCCTCAAGGATGGCCTGGCGCATGGGGGAACCAGCCTGCTGGGGCAGGGGGCTACCCAGGGGCTTCCT
ATCCTGGGGCCTACCCCGGGCAGGCACCCCCAGGGGCTTATCCTGGACAGGCACCTCCAGGCGCCTACCCTGGAGCA
CCTGGAGCTTATCCCGGAGCACCTGCACCTGGAGTCTACCCAGGGCCACCCAGCGGCCCTGGGGCCTACCCATCTTC
TGGACAGCCAAGTGCCCCCGGAGCCTACCCTGCCACTGGCCCCTATGGCGCCCCTGCTGGGCCACTGATTGTGCCTT
ATAACCTGCCTTTGCCTGGGGGAGTGGTGCCTCGCATGCTGATAACAATTCTGGGCACGGTGAAGCCCAATGCAAA
CAGAATTGCTTTAGATTTCCAAAGAGGGAATGATGTTGCCTTCCACTTTAACCCACGCTTCAATGAGAACAACAGG
AGAGTCATTGTTTGCAATACAAAGCTGGATAATAACTGGGGAAGGGAAGAAAGACAGTCGGTTTTCCCATTTGAA
AGTGGGAAACCATTCAAAATACAAGTACTGGTTGAACCTGACCACTTCAAGGTTGCAGTGAATGATGCTCACTTGT
TGCAGTACAATCATCGGGTTAAAAAACTCAATGAAATCAGCAAACTGGGAATTTCTGGTGACATAGACCTCACCAG
TGCTTCATATAACATGATACTCGAGCACCACCACCACCACCAC**TGA**

WT-NL genetic sequence:

CC**ATG**GCGGTCTTCACACTCGAAGATTTCGTTGGGGACTGGCGACAGACAGCCGGCTACAACCTGGACCAAGTCCT
TGAACAGGGAGGTGTGTCCAGTTTGTTTCAGAATCTCGGGGTGTCCGTAACTCCGATCCAAAGGATTGTCCTGAGC
GGTGAAAATGGGCTGAAGATCGACATCCATGTCATCATCCCGTATGAAGGTCTGAGCGGCGACCAAATGGGCCAGA

TCGAAAAAATTTTTAAGGTGGTGTACCCTGTGGATGATCATCACTTTAAGGTGATCCTGCACTATGGCACACTGGT
AATCGACGGGGTTACGCCGAACATGATCGACTATTTCGGACGGCCGTATGAAGGCATCGCCGTGTTCGACGGCAAA
AAGATCACTGTAACAGGGACCCTGTGGAACGGCAACAAAATTATCGACGAGCGCCTGATCAACCCCGACGGCTCCC
TGCTGTTCCGAGTAACCATCAACGGAGTGACCGGCTGGCGGCTGTGCGAACGCATTCTGGCGGGATCCCACCACCAC
CACCACCAC**TGA**

GFP-TT-G3 genetic sequence:
CC**ATG**TCCAAAGGAGAAGAGCTGTTCACTGGAGTGGTACCAATACTTGTGGAGTTGGACGGAGATGTGAACGGAC
ACAAATTTTCAGTCCGCGGGGAGGGGGAAGGGGATGCTACTATTGGCAAGCTGACGCTCAAATTCATCTGTACCAC
CGGAAAACTCCCTGTACCCTGGCCCACACTGGTGACAACTCTGACTTACGGCGTGCAATGTTTTAGCCGATACCCAG
ACCACATGAAGAGGCACGACTTTTTCAAAAGCGCAATGCCTGAAGGATACGTACAGGAAAGGACCATTTCTTTTAA
AGACGACGGGAAGTACAAAACCCGGGCAGTGGTGAAGTTTGAGGGCGATACCCTCGTCAATAGGATCGAATTGAAG
GGAACTGACTTCAAAGAAGATGGCAACATCCTGGGTCACAAGCTTGAGTATAACTTTAACTCCCACAACGTGTATA
TTACAGCCGACAAACAGAAGAATGGAATTAAGGCTAACTTCACTGTCAGACACAATGTCGAAGATGGCTCCGTGCA
GCTCGCCGATCACTATCAACAGAATACTCCTATCGGGGACGGCCCAGTCCTGCTGCCCGACAACCACTACCTGAGTA
CCCAGACTGTTCTGAGCAAAGATCCGAACGAGAAGCGCGACCACATGGTGCTGCATGAGTATGTCAACGCTGCGGG
AATTACCCTCGGCATGGACGAGCTGTACAAGGGATCCGGCGGCGGCAGCGGCGGCAGCGGCGGCAGCGGCGGCATG
GCGCGCATGAAACAGCTGGAAGATAAAGTGGAAGAACTGCTGAGCAAAAACTATCATCTGGAAAACCGCGTGGCGC
GCCTGGAAAAACTGGTGGGCGAACGCGGCGGCGGCAGCGGCGGCAGCGGCGGCGGCGGCAGCGGCGGCAGCGGCGA
ATTCGCAGACAATTTTTCGCTCCATGATGCGTTATCTGGGTCTGGAAACCCAAACCCTCAAGGATGGCCTGGCGCA
TGGGGGAACCAGCCTGCTGGGGCAGGGGGCTACCCAGGGGCTTCCTATCCTGGGGCCTACCCCGGGCAGGCACCCCC
AGGGGCTTATCCTGGACAGGCACCTCCAGGCGCCTACCCTGGAGCACCTGGAGCTTATCCCGGAGCACCTGCACCTG
GAGTCTACCCAGGGCCACCCAGCGGCCCTGGGGCCTACCCATCTTCTGGACAGCCAAGTGCCCCCGGAGCCTACCCT
GCCACTGGCCCCTATGGCGCCCCTGCTGGGCCACTGATTGTGCCTTATAACCTGCCTTTGCCTGGGGGAGTGGTGCC
TCGCATGCTGATAACAATTCTGGGCACGGTGAAGCCCAATGCAAACAGAATTGCTTTAGATTTCCAAAGAGGGAAT
GATGTTGCCTTCCACTTTAACCCACGCTTCAATGAGAACAACAGGAGAGTCATTGTTTGCAATACAAAGCTGGATA
ATAACTGGGGAAGGGAAGAAAGACAGTCGGTTTTCCCATTTGAAAGTGGGAAACCATTCAAAATACAAGTACTGG
TTGAACCTGACCACTTCAAGGTTGCAGTGAATGATGCTCACTTGTTGCAGTACAATCATCGGGTTAAAAAACTCAA
TGAAATCAGCAAACTGGGAATTTCTGGTGACATAGACCTCACCAGTGCTTCATATAACATGATACTCGAGCACCAC
CACCACCACCAC**TGA**

GFP-G3 genetic sequence:
CC**ATG**TCCAAAGGAGAAGAGCTGTTCACTGGAGTGGTACCAATACTTGTGGAGTTGGACGGAGATGTGAACGGAC
ACAAATTTTCAGTCCGCGGGGAGGGGGAAGGGGATGCTACTATTGGCAAGCTGACGCTCAAATTCATCTGTACCAC
CGGAAAACTCCCTGTACCCTGGCCCACACTGGTGACAACTCTGACTTACGGCGTGCAATGTTTTAGCCGATACCCAG
ACCACATGAAGAGGCACGACTTTTTCAAAAGCGCAATGCCTGAAGGATACGTACAGGAAAGGACCATTTCTTTTAA
AGACGACGGGAAGTACAAAACCCGGGCAGTGGTGAAGTTTGAGGGCGATACCCTCGTCAATAGGATCGAATTGAAG
GGAACTGACTTCAAAGAAGATGGCAACATCCTGGGTCACAAGCTTGAGTATAACTTTAACTCCCACAACGTGTATA
TTACAGCCGACAAACAGAAGAATGGAATTAAGGCTAACTTCACTGTCAGACACAATGTCGAAGATGGCTCCGTGCA
GCTCGCCGATCACTATCAACAGAATACTCCTATCGGGGACGGCCCAGTCCTGCTGCCCGACAACCACTACCTGAGTA
CCCAGACTGTTCTGAGCAAAGATCCGAACGAGAAGCGCGACCACATGGTGCTGCATGAGTATGTCAACGCTGCGGG
AATTACCCTCGGCATGGACGAGCTGTACAAGGGATCCGGCGGCGGCAGCGGCGGCAGCGGCGGCAGCGGCGGCGAA
TTCGCAGACAATTTTTCGCTCCATGATGCGTTATCTGGGTCTGGAAACCCAAACCCTCAAGGATGGCCTGGCGCAT
GGGGGAACCAGCCTGCTGGGGCAGGGGGCTACCCAGGGGCTTCCTATCCTGGGGCCTACCCCGGGCAGGCACCCCCA
GGGGCTTATCCTGGACAGGCACCTCCAGGCGCCTACCCTGGAGCACCTGGAGCTTATCCCGGAGCACCTGCACCTGG
AGTCTACCCAGGGCCACCCAGCGGCCCTGGGGCCTACCCATCTTCTGGACAGCCAAGTGCCCCCGGAGCCTACCCTG
CCACTGGCCCCTATGGCGCCCCTGCTGGGCCACTGATTGTGCCTTATAACCTGCCTTTGCCTGGGGGAGTGGTGCCT
CGCATGCTGATAACAATTCTGGGCACGGTGAAGCCCAATGCAAACAGAATTGCTTTAGATTTCCAAAGAGGGAATG

ATGTTGCCTTCCACTTTAACCCACGCTTCAATGAGAACAACAGGAGAGTCATTGTTTGCAATACAAAGCTGGATAA
TAACTGGGGAAGGGAAGAAAGACAGTCGGTTTTCCCATTTGAAAGTGGGAAACCATTCAAAATACAAGTACTGGT
TGAACCTGACCACTTCAAGGTTGCAGTGAATGATGCTCACTTGTTGCAGTACAATCATCGGGTTAAAAAACTCAAT
GAAATCAGCAAACTGGGAATTTCTGGTGACATAGACCTCACCAGTGCTTCATATAACATGATACTCGAGCACCACC
ACCACCACCAC**TGA**

<u>ChABC-TT-G3 genetic sequence:</u>

CC**ATG**GCCACATCGAATCCGGCTTTCGACCCCAAAAACTTAATGCAGAGTGAAATCTATCATTTTGCCCAGAATAA
CCCATTGGCAGACTTTTCAAGCGATAAAAACTCCATTCTGACGTTATCTGACAAGCGTTCCATTATGGGAAATCAA
TCCTTACTTTGGAAATGGAAAGGCGGCTCCAGTTTTACGTTACATAAAAAATTAATCGTGCCGACCGATAAGGAAG
CATCTAAAGCATGGGGACGCAGTAGTACGCCGGTGTTCTCCTTCTGGCTGTACAACGAGAAACCTATTGATGGGTA
CTTGACCATCGACTTCGGCGAAAAATTGATTTCAACTAGCGAAGCACAAGCTGGCTTTAAGGTTAAATTGGACTTC
ACCGGGTGGCGTACGGTCGGAGTCAGCCTTAACAATGACTTAGAGAATCGCGAAATGACCTTGAATGCTACGAATA
CGTCCAGCGATGGAACCCAGGATAGCATCGGTCGTAGTCTGGGAGCAAAGGTGGACAGTATCCGCTTCAAAGCTCC
GAGCAACGTTTCACAGGGGGAAATTTATATCGACCGTATTATGTTCAGTGTCGACGACGCCCGTTATCAATGGAGT
GACTATCAAGTAAAGACCCGTCTGTCAGAACCTGAGATCCAGTTTCATAATGTGAAGCCGCAGTTGCCCGTAACGC
CCGAAAATTTGGCAGCTATTGATCTTATCCGCCAGCGCCTGATCAATGAGTTTGTCGGCGGGGAGAAAGAAACCAA
CCTGGCGCTGGAAGAAAATATCTCAAAACTGAAAAGTGACTTTGATGCACTTAATATCCACACGCTGGCGAACGGG
GGAACCCAAGGACGTCACTTAATTACTGACAAGCAAATTATCATCTATCAACCCGAGAATCTTAACTCACAAGATA
AACAGTTATTTGACAATTATGTCATCTTAGGTAACTACACGACCTTGATGTTTAACATCTCTCGCGCCTATGTTTT
GGAAAAAGACCCCACTCAAAAGGCACAATTAAAGCAGATGTACCTTCTGATGACCAAACATCTTTTGGATCAGGGA
TTCGTGAAAGGTAGTGCGTTAGTAACAACACACCATTGGGGGTACAGTTCGCGCTGGTGGTATATCAGTACTTTGT
TAATGTCTGATGCATTGAAGGAGGCGAATCTTCAGACGCAGGTTTACGATTCCCTGCTGTGGTATTCGCGTGAGTT
CAAATCTTCGTTCGATATGAAGGTCAGCGCTGACTCTTCGGATTTGGATTACTTTAATACCCTTTCGCGCCAGCAC
TTGGCCTTGTTACTGCTGGAGCCAGACGACCAGAAACGCATCAACCTTGTCAACACGTTTAGTCACTATATCACAG
GAGCGCTTACTCAAGTACCGCCGGGGGGAAAAGATGGTCTTCGTCCAGATGGTACGGCCTGGCGCCATGAGGGTAA
CTATCCGGGCTATTCCTTTCCGGCATTTAAGAATGCGTCCCAACTGATTTACCTTCTGCGTGATACCCCATTTTCAG
TGGGAGAATCTGGATGGAACAACTTGAAGAAAGCGATGGTCTCTGCTTGGATTTACTCTAACCCAGAAGTTGGATT
ACCATTAGCAGGCCGTCACCCATTCAATAGTCCCTCCCTTAAAAGTGTCGCCCAGGGGTATTATTGGTTAGCTATG
TCTGCAAAGTCGAGTCCTGACAAAACCCTGGCGTCTATCTACTTGGCCATCTCCGATAAAACTCAAAACGAATCAA
CCGCCATTTTTGGCGAAACCATCACGCCGGCTTCTTTGCCGCAGGGGTTCTACGCCTTTAATGGAGGCGCCTTCGGC
ATTCATCGTTGGCAGGATAAGATGGTTACATTGAAAGCGTACAATACCAACGTATGGAGTTCCGAGATCTATAACA
AGGATAATCGCTATGGCCGTTATCAGTCTCACGGGGTGGCTCAAATTGTCTCTAACGGTAGTCAATTAAGTCAAGG
TTATCAACAGGAGGGATGGGACTGGAACCGCATGCCTGGGGCAACAACTATCCATCTGCCGCTGAAGGATTTAGAT
AGCCCTAAGCCACATACACTGATGCAGCGTGGTGAACGTGGCTTCTCGGGTACTAGTTCACTGGAGGGGCAATACG
GGATGATGGCATTTGACTTAATCTATCCAGCTAATTTAGAGCGCTTCGACCCCAATTTTACTGCTAAGAAGAGCGT
CCTGGCAGCGGATAACCACCTGATCTTTATTGGAAGCAATATTAATAGCTCGGACAAGAACAAGAATGTTGAGACG
ACACTTTTTCAGCATGCCATCACTCCAACTTTGAATACGCTGTGGATCAATGGACAAAAGATCGAAAATATGCCAT
ACCAGACAACACTTCAGCAGGGTGACTGGTTGATTGACTCGAATGGGAACGGGTACTTAATTACACAAGCCGAAAA
AGTGAATGTCTCGCGCCAGCACCAAGTTTCGGCGGAGAATAAAAACCGTCAACCTACTGAGGGCAACTTCAGCTCT
GCCTGGATTGATCACAGTACTCGCCCAAAAGACGCATCATATGAGTACATGGTATTCTTGGATGCTACGCCTGAGA
AAATGGGAGAGATGGCTCAAAAGTTCCGTGAGAACAACGGATTGTACCAAGTTCTGCGCAAGGATAAAGATGTGC
ATATCATTTTAGATAAACTTTCCAATGTAACGGGGTATGCCTTTTATCAACCCGCCAGTATTGAGGATAAATGGAT
CAAGAAAGTGAATAAGCCCGCCATCGTAATGACTCATCGCCAGAAAGACACTCTTATTGTCTCGGCGGTAACACCC
GATCTTAATATGACTCGCCAGAAAGCAGCTACTCCCGTAACAATCAACGTTACGATCAATGGGAAGTGGCAAAGTG
CTGATAAGAACAGTGAGGTTAAATACCAGGTGTCCGGCGATAATACGGAGTTGACGTTTACATCTTACTTCGGCAT
CCCACAAGAAATTAAGTTGTCCCCTTTGCCGGGATCCGGCGGCGGCAGCGGCGGCAGCGGCGGCAGCGGCGGCATG
GCGCGCATGAAACAGCTGGAAGATAAAGTGGAAGAACTGCTGAGCAAAAACTATCATCTGGAAAACCGCGTGGCGC
GCCTGGAAAAACTGGTGGGCGAACGCGGCGGCGGCAGCGGCGGCAGCGGCGGCGGCGGCAGCGGCGGCAGCGGCGA

ATTCGCAGACAATTTTTCGCTCCATGATGCGTTATCTGGGTCTGGAAACCCAAACCCTCAAGGATGGCCTGGCGCA
TGGGGGAACCAGCCTGCTGGGGCAGGGGGCTACCCAGGGGCTTCCTATCCTGGGGCCTACCCCGGGCAGGCACCCCC
AGGGGCTTATCCTGGACAGGCACCTCCAGGCGCCTACCCTGGAGCACCTGGAGCTTATCCCGGAGCACCTGCACCTG
GAGTCTACCCAGGGCCACCCAGCGGCCCTGGGGCCTACCCATCTTCTGGACAGCCAAGTGCCCCCGGAGCCTACCCT
GCCACTGGCCCCTATGGCGCCCCTGCTGGGCCACTGATTGTGCCTTATAACCTGCCTTTGCCTGGGGGAGTGGTGCC
TCGCATGCTGATAACAATTCTGGGCACGGTGAAGCCCAATGCAAACAGAATTGCTTTAGATTTCCAAAGAGGGAAT
GATGTTGCCTTCCACTTTAACCCACGCTTCAATGAGAACAACAGGAGAGTCATTGTTTGCAATACAAAGCTGGATA
ATAACTGGGGAAGGGAAGAAAGACAGTCGGTTTTCCCATTTGAAAGTGGGAAACCATTCAAAATACAAGTACTGG
TTGAACCTGACCACTTCAAGGTTGCAGTGAATGATGCTCACTTGTTGCAGTACAATCATCGGGTTAAAAAACTCAA
TGAAATCAGCAAACTGGGAATTTCTGGTGACATAGACCTCACCAGTGCTTCATATAACATGATACTCGAGCACCAC
CACCACCACCAC**TGA**

<u>ChABC-G3 genetic sequence:</u>

CC**ATG**GCCACATCGAATCCGGCTTTCGACCCCAAAAACTTAATGCAGAGTGAAATCTATCATTTTGCCCAGAATAA
CCCATTGGCAGACTTTTCAAGCGATAAAAACTCCATTCTGACGTTATCTGACAAGCGTTCCATTATGGGAAATCAA
TCCTTACTTTGGAAATGGAAAGGCGGCTCCAGTTTTACGTTACATAAAAAATTAATCGTGCCGACCGATAAGGAAG
CATCTAAAGCATGGGGACGCAGTAGTACGCCGGTGTTCTCCTTCTGGCTGTACAACGAGAAACCTATTGATGGGTA
CTTGACCATCGACTTCGGCGAAAAATTGATTTCAACTAGCGAAGCACAAGCTGGCTTTAAGGTTAAATTGGACTTC
ACCGGGTGGCGTACGGTCGGAGTCAGCCTTAACAATGACTTAGAGAATCGCGAAATGACCTTGAATGCTACGAATA
CGTCCAGCGATGGAACCCAGGATAGCATCGGTCGTAGTCTGGGAGCAAAGGTGGACAGTATCCGCTTCAAAGCTCC
GAGCAACGTTTCACAGGGGGAAATTTATATCGACCGTATTATGTTCAGTGTCGACGACGCCCGTTATCAATGGAGT
GACTATCAAGTAAAGACCCGTCTGTCAGAACCTGAGATCCAGTTTCATAATGTGAAGCCGCAGTTGCCCGTAACGC
CCGAAAATTTGGCAGCTATTGATCTTATCCGCCAGCGCCTGATCAATGAGTTTGTCGGCGGGGAGAAAGAAACCAA
CCTGGCGCTGGAAGAAAATATCTCAAAACTGAAAAGTGACTTTGATGCACTTAATATCCACACGCTGGCGAACGGG
GGAACCCAAGGACGTCACTTAATTACTGACAAGCAAATTATCATCTATCAACCCGAGAATCTTAACTCACAAGATA
AACAGTTATTTGACAATTATGTCATCTTAGGTAACTACACGACCTTGATGTTTAACATCTCTCGCGCCTATGTTTT
GGAAAAAGACCCCACTCAAAAGGCACAATTAAAGCAGATGTACCTTCTGATGACCAAACATCTTTTGGATCAGGGA
TTCGTGAAAGGTAGTGCGTTAGTAACAACACACCATTGGGGGTACAGTTCGCGCTGGTGGTATATCAGTACTTTGT
TAATGTCTGATGCATTGAAGGAGGCGAATCTTCAGACGCAGGTTTACGATTCCCTGCTGTGGTATTCGCGTGAGTT
CAAATCTTCGTTCGATATGAAGGTCAGCGCTGACTCTTCGGATTTGGATTACTTTAATACCCTTTCGCGCCAGCAC
TTGGCCTTGTTACTGCTGGAGCCAGACGACCAGAAACGCATCAACCTTGTCAACACGTTTAGTCACTATATCACAG
GAGCGCTTACTCAAGTACCGCCGGGGGGAAAAGATGGTCTTCGTCCAGATGGTACGGCCTGGCGCCATGAGGGTAA
CTATCCGGGCTATTCCTTTCCGGCATTTAAGAATGCGTCCCAACTGATTTACCTTCTGCGTGATACCCCATTTTCAG
TGGGAGAATCTGGATGGAACAACTTGAAGAAAGCGATGGTCTCTGCTTGGATTTACTCTAACCCAGAAGTTGGATT
ACCATTAGCAGGCCGTCACCCATTCAATAGTCCCTCCCTTAAAAGTGTCGCCCAGGGGTATTATTGGTTAGCTATG
TCTGCAAAGTCGAGTCCTGACAAAACCCTGGCGTCTATCTACTTGGCCATCTCCGATAAAACTCAAAACGAATCAA
CCGCCATTTTTGGCGAAACCATCACGCCGGCTTCTTTGCCGCAGGGGTTCTACGCCTTTAATGGAGGCGCCTTCGGC
ATTCATCGTTGGCAGGATAAGATGGTTACATTGAAAGCGTACAATACCAACGTATGGAGTTCCGAGATCTATAACA
AGGATAATCGCTATGGCCGTTATCAGTCTCACGGGGTGGCTCAAATTGTCTCTAACGGTAGTCAATTAAGTCAAGG
TTATCAACAGGAGGGATGGGACTGGAACCGCATGCCTGGGGCAACAACTATCCATCTGCCGCTGAAGGATTTAGAT
AGCCCTAAGCCACATACACTGATGCAGCGTGGTGAACGTGGCTTCTCGGGTACTAGTTCACTGGAGGGGCAATACG
GGATGATGGCATTTGACTTAATCTATCCAGCTAATTTAGAGCGCTTCGACCCCAATTTTACTGCTAAGAAGAGCGT
CCTGGCAGCGGATAACCACCTGATCTTTATTGGAAGCAATATTAATAGCTCGGACAAGAACAAGAATGTTGAGACG
ACACTTTTTCAGCATGCCATCACTCCAACTTTGAATACGCTGTGGATCAATGGACAAAAGATCGAAAATATGCCAT
ACCAGACAACACTTCAGCAGGGTGACTGGTTGATTGACTCGAATGGGAACGGGTACTTAATTACACAAGCCGAAAA
AGTGAATGTCTCGCGCCAGCACCAAGTTTCGGCGGAGAATAAAAACCGTCAACCTACTGAGGGCAACTTCAGCTCT
GCCTGGATTGATCACAGTACTCGCCCAAAAGACGCATCATATGAGTACATGGTATTCTTGGATGCTACGCCTGAGA
AAATGGGAGAGATGGCTCAAAAGTTCCGTGAGAACAACGGATTGTACCAAGTTCTGCGCAAGGATAAAGATGTGC
ATATCATTTTAGATAAACTTTCCAATGTAACGGGGTATGCCTTTTATCAACCCGCCAGTATTGAGGATAAATGGAT

CAAGAAAGTGAATAAGCCCGCCATCGTAATGACTCATCGCCAGAAAGACACTCTTATTGTCTCGGCGGTAACACCC
GATCTTAATATGACTCGCCAGAAAGCAGCTACTCCCGTAACAATCAACGTTACGATCAATGGGAAGTGGCAAAGTG
CTGATAAGAACAGTGAGGTTAAATACCAGGTGTCCGGCGATAATACGGAGTTGACGTTTACATCTTACTTCGGCAT
CCCACAAGAAATTAAGTTGTCCCCTTTGCCGGGATCCGGTGGCGGCTCAGGAGGCAGCGGAGGTTCAGGTGGGGAA
TTCATGGCTGACAACTTCAGTTTGCATGATGCTTTAAGTGGCTCCGGGAACCCGAACCCTCAAGGCTGGCCAGGGG
CATGGGGTAACCAACCGGCGGGAGCAGGAGGTTATCCCGGAGCGAGCTACCCTGGAGCCTACCCAGGACAGGCTCC
GCCTGGCGCGTATCCTGGGCAGGCTCCACCAGGTGCCTACCCGGGGGCACCCGGGGCATATCCGGGAGCTCCTGCTC
CCGGAGTTTATCCGGGTCCTCCTTCAGGACCGGGAGCGTATCCATCGAGCGGCCAGCCCAGTGCAACGGGTGCGTAC
CCAGCCACGGGCCCCTATGGAGCCCCAGCGGGACCATTAATTGTACCCTATAATCTTCCGCTTCCAGGCGGCGTAGT
TCCTCGTATGTTAATTACCATTTTAGGAACAGTAAAACCAAATGCTAATCGTATTGCATTAGACTTTCAACGTGGC
AACGACGTAGCTTTTCATTTTAACCCACGTTTTAACGAAAACAATCGTCGTGTCATTGTATGCAATACAAAACTGG
ATAATAATTGGGGACGCGAGGAGCGCCAGAGTGTGTTCCCATTCGAGTCGGGCAAACCATTCAAAATTCAGGTATT
GGTCGAGCCAGACCATTTCAAGGTCGCGGTCAATGACGCTCACTTATTACAGTACAATCATCGCGTAAAGAAACTG
AATGAGATCAGTAAGTTAGGAATCTCCGGAGACATTGATCTTACAAGTGCGAGTTACACGATGATTCACCACCACC
ACCACCAC**TGA**AAGCTTCTCGAG

TT-GFP genetic sequence:
CC**ATG**GCGCGCATGAAACAGCTGGAAGATAAAGTGGAAGAACTGCTGAGCAAAAACTATCATCTGGAAAACCGCG
TGGCGCGCCTGGAAAAACTGGTGGGCGAACGCGGATCCGGCGGCGGCAGCGGCGGCAGCGGCGGCGGCGGCAGCGG
CGGCAGCGGCGAATTCTCCAAAGGAGAAGAGCTGTTCACTGGAGTGGTACCAATACTTGTGGAGTTGGACGGAGAT
GTGAACGGACACAAATTTTCAGTCCGCGGGGAGGGGGAAGGGGATGCTACTATTGGCAAGCTGACGCTCAAATTCA
TCTGTACCACCGGAAAACTCCCTGTACCCTGGCCCACACTGGTGACAACTCTGACTTACGGCGTGCAATGTTTTAGC
CGATACCCAGACCACATGAAGAGGCACGACTTTTTCAAAAGCGCAATGCCTGAAGGATACGTACAGGAAAGGACCA
TTTCTTTTAAAGACGACGGGAAGTACAAAACCCGGGCAGTGGTGAAGTTTGAGGGCGATACCCTCGTCAATAGGAT
CGAATTGAAGGGAACTGACTTCAAAGAAGATGGCAACATCCTGGGTCACAAGCTTGAGTATAACTTTAACTCCCAC
AACGTGTATATTACAGCCGACAAACAGAAGAATGGAATTAAGGCTAACTTCACTGTCAGACACAATGTCGAAGAT
GGCTCCGTGCAGCTCGCCGATCACTATCAACAGAATACTCCTATCGGGGACGGCCCAGTCCTGCTGCCCGACAACCA
CTACCTGAGTACCCAGACTGTTCTGAGCAAAGATCCGAACGAGAAGCGCGACCACATGGTGCTGCATGAGTATGTC
AACGCTGCGGGAATTACCCTCGGCATGGACGAGCTGTACAAGCTCGAGCACCACCACCACCACCAC**TGA**

Supplementary Note 2. Amino acid sequences

NL-TT-G3 amino acid sequence:
MAVFTLEDFVGDWRQTAGYNLDQVLEQGGVSSLFQNLGVSVTPIQRIVLSGENGLKIDIHVIIPYEGLSGDQMGQIEKI FKVVYPVDDHHFKVILHYGTLVIDGVTPNMIDYFGRPYEGIAVFDGKKITVTGTLWNGNKIIDERLINPDGSLLFRVTI NGVTGWRLCERILAGSGGGSGGSGGSGGMARMKQLEDKVEELLSKNYHLENRVARLEKLVGERGGGSGGSGGGGSG GSGEFADNFSLHDALSGSGNPNPQGWPGAWGNQPAGAGGYPGASYPGAYPGQAPPGAYPGQAPPGAYPGAPGAYP GAPAPGVYPGPPSGPGAYPSSGQPSAPGAYPATGPYGAPAGPLIVPYNLPLPGGVVPRMLITILGTVKPNANRIALDFQ RGNDVAFHFNPRFNENNRRVIVCNTKLDNNWGREERQSVFPFESGKPFKIQVLVEPDHFKVAVNDAHLLQYNHRV KKLNEISKLGISGDIDLTSASYNMILEHHHHHH **Stop**

NL-G3 amino acid sequence:
MAVFTLEDFVGDWRQTAGYNLDQVLEQGGVSSLFQNLGVSVTPIQRIVLSGENGLKIDIHVIIPYEGLSGDQMGQIEKI FKVVYPVDDHHFKVILHYGTLVIDGVTPNMIDYFGRPYEGIAVFDGKKITVTGTLWNGNKIIDERLINPDGSLLFRVTI NGVTGWRLCERILAGSGGGSGGSGGSGGEFADNFSLHDALSGSGNPNPQGWPGAWGNQPAGAGGYPGASYPGAYP GQAPPGAYPGQAPPGAYPGAPGAYPGAPAPGVYPGPPSGPGAYPSSGQPSAPGAYPATGPYGAPAGPLIVPYNLPLPG GVVPRMLITILGTVKPNANRIALDFQRGNDVAFHFNPRFNENNRRVIVCNTKLDNNWGREERQSVFPFESGKPFKIQ VLVEPDHFKVAVNDAHLLQYNHRVKKLNEISKLGISGDIDLTSASYNMILEHHHHHH **Stop**

WT-NL amino acid sequence:
MAVFTLEDFVGDWRQTAGYNLDQVLEQGGVSSLFQNLGVSVTPIQRIVLSGENGLKIDIHVIIPYEGLSGDQMGQIEKI FKVVYPVDDHHFKVILHYGTLVIDGVTPNMIDYFGRPYEGIAVFDGKKITVTGTLWNGNKIIDERLINPDGSLLFRVTI NGVTGWRLCERILAGSHHHHHH **Stop**

GFP-TT-G3 amino acid sequence:
MSKGEELFTGVVPILVELDGDVNGHKFSVRGEGEGDATIGKLTLKFICTTGKLPVPWPTLVTTLTYGVQCFSRYPDH MKRHDFFKSAMPEGYVQERTISFKDDGKYKTRAVVKFEGDTLVNRIELKGTDFKEDGNILGHKLEYNFNSHNVYIT ADKQKNGIKANFTVRHNVEDGSVQLADHYQQNTPIGDGPVLLPDNHYLSTQTVLSKDPNEKRDHMVLHEYVNAAG ITLGMDELYKGSGGGSGGSGGSGGMARMKQLEDKVEELLSKNYHLENRVARLEKLVGERGGGSGGSGGGGSGGSGEF ADNFSLHDALSGSGNPNPQGWPGAWGNQPAGAGGYPGASYPGAYPGQAPPGAYPGQAPPGAYPGAPGAYPGAPAP GVYPGPPSGPGAYPSSGQPSAPGAYPATGPYGAPAGPLIVPYNLPLPGGVVPRMLITILGTVKPNANRIALDFQRGND VAFHFNPRFNENNRRVIVCNTKLDNNWGREERQSVFPFESGKPFKIQVLVEPDHFKVAVNDAHLLQYNHRVKKLN EISKLGISGDIDLTSASYNMILEHHHHHH **Stop**

GFP-G3 amino acid sequence:
MSKGEELFTGVVPILVELDGDVNGHKFSVRGEGEGDATIGKLTLKFICTTGKLPVPWPTLVTTLTYGVQCFSRYPDH MKRHDFFKSAMPEGYVQERTISFKDDGKYKTRAVVKFEGDTLVNRIELKGTDFKEDGNILGHKLEYNFNSHNVYIT ADKQKNGIKANFTVRHNVEDGSVQLADHYQQNTPIGDGPVLLPDNHYLSTQTVLSKDPNEKRDHMVLHEYVNAAG ITLGMDELYKGSGGGSGGSGGSGGEFADNFSLHDALSGSGNPNPQGWPGAWGNQPAGAGGYPGASYPGAYPGQAPP GAYPGQAPPGAYPGAPGAYPGAPAPGVYPGPPSGPGAYPSSGQPSAPGAYPATGPYGAPAGPLIVPYNLPLPGGVVPR MLITILGTVKPNANRIALDFQRGNDVAFHFNPRFNENNRRVIVCNTKLDNNWGREERQSVFPFESGKPFKIQVLVEP DHFKVAVNDAHLLQYNHRVKKLNEISKLGISGDIDLTSASYNMILEHHHHHH **Stop**

<u>TT-GFP amino acid sequence:</u>
MARMKQLEDKVEELLSKNYHLENRVARLEKLVGERGSGGGSGGSGGGGSGGSGEFSKGEELFTGVVPILVELDGDVN
GHKFSVRGEGEGDATIGKLTLKFICTTGKLPVPWPTLVTTLTYGVQCFSRYPDHMKRHDFFKSAMPEGYVQERTISF
KDDGKYKTRAVVKFEGDTLVNRIELKGTDFKEDGNILGHKLEYNFNSHNVYITADKQKNGIKANFTVRHNVEDGSV
QLADHYQQNTPIGDGPVLLPDNHYLSTQTVLSKDPNEKRDHMVLHEYVNAAGITLGMDELYKLEHHHHHH **Stop**

<u>ChABC-TT-G3 amino acid sequence:</u>
MATSNPAFDPKNLMQSEIYHFAQNNPLADFSSDKNSILTLSDKRSIMGNQSLLWKWKGGSSFTLHKKLIVPTDKEAS
KAWGRSSTPVFSFWLYNEKPIDGYLTIDFGEKLISTSEAQAGFKVKLDFTGWRTVGVSLNNDLENREMTLNATNTS
SDGTQDSIGRSLGAKVDSIRFKAPSNVSQGEIYIDRIMFSVDDARYQWSDYQVKTRLSEPEIQFHNVKPQLPVTPENL
AAIDLIRQRLINEFVGGEKETNLALEENISKLKSDFDALNIHTLANGGTQGRHLITDKQIIIYQPENLNSQDKQLFDNY
VILGNYTTLMFNISRAYVLEKDPTQKAQLKQMYLLMTKHLLDQGFVKGSALVTTHHWGYSSRWWYISTLLMSDAL
KEANLQTQVYDSLLWYSREFKSSFDMKVSADSSDLDYFNTLSRQHLALLLLEPDDQKRINLVNTFSHYITGALTQVP
PGGKDGLRPDGTAWRHEGNYPGYSFPAFKNASQLIYLLRDTPFSVGESGWNNLKKAMVSAWIYSNPEVGLPLAGRH
PFNSPSLKSVAQGYYWLAMSAKSSPDKTLASIYLAISDKTQNESTAIFGETITPASLPQGFYAFNGGAFGIHRWQDKM
VTLKAYNTNVWSSEIYNKDNRYGRYQSHGVAQIVSNGSQLSQGYQQEGWDWNRMPGATTIHLPLKDLDSPKPHTL
MQRGERGFSGTSSLEGQYGMMAFDLIYPANLERFDPNFTAKKSVLAADNHLIFIGSNINSSDKNKNVETTLFQHAIT
PTLNTLWINGQKIENMPYQTTLQQGDWLIDSNGNGYLITQAEKVNVSRQHQVSAENKNRQPTEGNFSSAWIDHST
RPKDASYEYMVFLDATPEKMGEMAQKFRENNGLYQVLRKDKDVHIILDKLSNVTGYAFYQPASIEDKWIKKVNKPA
IVMTHRQKDTLIVSAVTPDLNMTRQKAATPVTINVTINGKWQSADKNSEVKYQVSGDNTELTFTSYFGIPQEIKLSP
LPGSGGGSGGSGGSGGMARMKQLEDKVEELLSKNYHLENRVARLEKLVGERGGGSGGSGGGGSGGSGEFADNFSLH
DALSGSGNPNPQGWPGAWGNQPAGAGGYPGASYPGAYPGQAPPGAYPGQAPPGAYPGAPGAYPGAPAPGVYPGPP
SGPGAYPSSGQPSAPGAYPATGPYGAPAGPLIVPYNLPLPGGVVPRMLITILGTVKPNANRIALDFQRGNDVAFHFNP
RFNENNRRVIVCNTKLDNNWGREERQSVFPFESGKPFKIQVLVEPDHFKVAVNDAHLLQYNHRVKKLNEISKLGISG
DIDLTSASYNMILEHHHHHH **Stop**

<u>ChABC-G3 amino acid sequence:</u>
MATSNPAFDPKNLMQSEIYHFAQNNPLADFSSDKNSILTLSDKRSIMGNQSLLWKWKGGSSFTLHKKLIVPTDKEAS
KAWGRSSTPVFSFWLYNEKPIDGYLTIDFGEKLISTSEAQAGFKVKLDFTGWRTVGVSLNNDLENREMTLNATNTS
SDGTQDSIGRSLGAKVDSIRFKAPSNVSQGEIYIDRIMFSVDDARYQWSDYQVKTRLSEPEIQFHNVKPQLPVTPENL
AAIDLIRQRLINEFVGGEKETNLALEENISKLKSDFDALNIHTLANGGTQGRHLITDKQIIIYQPENLNSQDKQLFDNY
VILGNYTTLMFNISRAYVLEKDPTQKAQLKQMYLLMTKHLLDQGFVKGSALVTTHHWGYSSRWWYISTLLMSDAL
KEANLQTQVYDSLLWYSREFKSSFDMKVSADSSDLDYFNTLSRQHLALLLLEPDDQKRINLVNTFSHYITGALTQVP
PGGKDGLRPDGTAWRHEGNYPGYSFPAFKNASQLIYLLRDTPFSVGESGWNNLKKAMVSAWIYSNPEVGLPLAGRH
PFNSPSLKSVAQGYYWLAMSAKSSPDKTLASIYLAISDKTQNESTAIFGETITPASLPQGFYAFNGGAFGIHRWQDKM
VTLKAYNTNVWSSEIYNKDNRYGRYQSHGVAQIVSNGSQLSQGYQQEGWDWNRMPGATTIHLPLKDLDSPKPHTL
MQRGERGFSGTSSLEGQYGMMAFDLIYPANLERFDPNFTAKKSVLAADNHLIFIGSNINSSDKNKNVETTLFQHAIT
PTLNTLWINGQKIENMPYQTTLQQGDWLIDSNGNGYLITQAEKVNVSRQHQVSAENKNRQPTEGNFSSAWIDHST
RPKDASYEYMVFLDATPEKMGEMAQKFRENNGLYQVLRKDKDVHIILDKLSNVTGYAFYQPASIEDKWIKKVNKPA
IVMTHRQKDTLIVSAVTPDLNMTRQKAATPVTINVTINGKWQSADKNSEVKYQVSGDNTELTFTSYFGIPQEIKLSP
LPGSGGGSGGSGGSGGEFMADNFSLHDALSGSGNPNPQGWPGAWGNQPAGAGGYPGASYPGAYPGQAPPGAYPGQ
APPGAYPGAPGAYPGAPAPGVYPGPPSGPGAYPSSGQPSATGAYPATGPYGAPAGPLIVPYNLPLPGGVVPRMLITILG
TVKPNANRIALDFQRGNDVAFHFNPRFNENNRRVIVCNTKLDNNWGREERQSVFPFESGKPFKIQVLVEPDHFKVA
VNDAHLLQYNHRVKKLNEISKLGISGDIDLTSASYTMIHHHHHH **Stop**
